# Single-nucleus atlas of cell-type specific genetic regulation in the human brain

**DOI:** 10.1038/s41588-026-02733-5

**Published:** 2026-09-23

**Authors:** Biao Zeng, Hui Yang, Prashant N. M, Sanan Venkatesh, Deepika Mathur, Pavan Auluck, David A. Bennett, Stefano Marenco, Vahram Haroutunian, PsychAD Consortium, Biao Zeng, Hui Yang, Prashant N. M, Sanan Venkatesh, Deepika Mathur, Stefano Marenco, Aram Hong, Athan Z. Li, Chenfeng He, Chirag Gupta, Christian Dillard, Christian Porras, Clara Casey, Colleen A. McClung, Collin Spencer, Daifeng Wang, David A. Bennett, David Burstein, Fotios Tsetsos, Gabriel E. Hoffman, Gennadi Ryan, Jennifer Monteiro Fortes, Jerome J. Choi, Kalpana H. Arachchilage, Karen Therrien, Lars J. Jensen, Lisa L. Barnes, Logan C. Dumitrescu, Lyra Sheu, Madeline R. Scott, Marcela Alvia, Marios Anyfantakis, Maxim Signaevsky, Mikaela Koutrouli, Milos Pjanic, Monika Ahirwar, Nicolas Y. Masse, Noah Cohen Kalafut, Pavan K. Auluck, Pavel Katsel, Pengfei Dong, Pramod B. Chandrashekar, Rachel Bercovitch, Roman Kosoy, Saniya Khullar, Sarah R. Murphy, Sayali A. Alatkar, Seon Kinrot, Stathis Argyriou, Steven Finkbeiner, Steven P. Kleopoulos, Tereza Clarence, Timothy J. Hohman, Ting Jin, Vahram Haroutunian, Vivek G. Ramaswamy, Xiang Huang, Xinyi Wang, Zhenyi Wu, Zhiping Shao, Georgios Voloudakis, Donghoon Lee, John F. Fullard, Jaroslav Bendl, Kiran Girdhar, Panos Roussos, Georgios Voloudakis, Donghoon Lee, John F. Fullard, Jaroslav Bendl, Kiran Girdhar, Gabriel E. Hoffman, Panos Roussos

**Affiliations:** 1 Center for Disease Neurogenomics, Icahn School of Medicine at Mount Sinai, New York, NY USA; 2 Friedman Brain Institute, Icahn School of Medicine at Mount Sinai, New York, NY USA; 3 Department of Psychiatry, Icahn School of Medicine at Mount Sinai, New York, NY USA; 4 Department of Genetics and Genomic Sciences, Icahn School of Medicine at Mount Sinai, New York, NY USA; 5 Human Brain Collection Core, National Institute of Mental Health–Intramural Research Program, Bethesda, MD USA; 6 Rush Alzheimer’s Disease Center, Rush University Medical Center, Chicago, IL USA; 7 Mental Illness Research, Education and Clinical Center VISN2, James J. Peters VA Medical Center, Bronx, NY USA; 8 Center for Precision Medicine and Translational Therapeutics, James J. Peters VA Medical Center, Bronx, NY USA; 9 Department of Artificial Intelligence and Human Health, Icahn School of Medicine at Mount Sinai, New York, NY USA; 10 Department of Computer Sciences, University of Wisconsin–Madison, Madison, WI USA; 11 Waisman Center, University of Wisconsin–Madison, Madison, WI USA; 12 Department of Biostatistics and Medical Informatics, University of Wisconsin–Madison, Madison, WI USA; 13 Department of Population Health Sciences, University of Wisconsin–Madison, Madison, WI USA; 14 Department of Psychiatry, University of Pittsburgh School of Medicine, Pittsburgh, PA USA; 15 Rush Alzheimer’s Disease Center and Department of Neurological Sciences, Rush University Medical Center, Chicago, IL USA; 16 Center for Systems and Therapeutics, Gladstone Institutes, San Francisco, CA USA; 17 Taube/Koret Center for Neurodegenerative Disease Research, Gladstone Institutes, San Francisco, CA USA; 18 Novo Nordisk Foundation Center for Protein Research, Faculty of Health and Medical Sciences, University of Copenhagen, Copenhagen, Denmark; 19 Vanderbilt Genetics Institute, Vanderbilt University Medical Center, Nashville, TN USA; 20 Vanderbilt Memory & Alzheimer’s Center, Vanderbilt University Medical Center, Nashville, TN USA; 21 Department of Neurology, University of California San Francisco, San Francisco, CA USA; 22 Department of Physiology, University of California San Francisco, San Francisco, CA USA; 23 Neuroscience and Biomedical Sciences Graduate Programs, University of California San Francisco, San Francisco, CA USA

**Keywords:** Gene expression, Genomics

## Abstract

Genetic risk variants for common diseases are predominantly located in non-coding regulatory regions and modulate gene expression. Although bulk tissue studies have elucidated shared mechanisms of regulatory and disease-associated genetics, the cellular specificity of these mechanisms remains largely unexplored. Here we present a comprehensive, single-nucleus multi-ancestry atlas of genetic regulation of gene expression in the human prefrontal cortex, comprising 5.6 million nuclei from 1,384 donors of diverse ancestries. Through multi-resolution analyses spanning eight major cell classes and 27 subclasses, we identify genetic regulation for 14,258 genes, with 981 showing cell type-specific regulatory effects at the class level and 857 at the subclass level. Colocalization of genetic variants associated with gene regulation and disease traits uncovers novel cell type-specific genes implicated in Alzheimer’s disease, schizophrenia and other disorders that were not detectable in bulk tissue analyses. Analysis of dynamic genetic regulation at the single-nucleus level identifies 2,073 genes with regulatory effects that vary across developmental trajectories, inferred from a broad age range of donors. We also uncover 1,655 genes with *trans*-regulatory effects, revealing distal regulation of gene expression. This high-resolution atlas provides insight into the cell type-specific regulatory architecture of the human brain, and offers novel mechanistic targets for understanding the genetic basis of neuropsychiatric and neurodegenerative diseases.

## Abstract (web summary)

Multi-resolution genetic regulatory atlas of the human brain from single-nucleus transcriptomics of 1,384 donors of diverse ancestries reveals cell type-specific regulation underlying genetic risk of neuropsychiatric and neurodegenerative diseases.

## Main

The human brain is composed of an array of cell types with a range of biological functions, cellular interactions and unique contributions to the genetic risk for neurodegenerative and neuropsychiatric traits^1–3^. Genetic risk variants identified by large-scale genome-wide association studies (GWAS) are predominantly located in non-coding regions and have a regulatory role in modifying gene expression^4–9^. Understanding the role of shared regulatory and disease risk effects in bulk tissue has yielded novel insight into the genes and molecular mechanisms underlying disease biology^7,10–12^. Yet analysis of bulk tissue cannot examine genetic regulatory effects that vary across the array of cell types, and it overlooks the distinct role of these cell types in disease biology^13–15^. Recent efforts to increase the cell-type resolution of genetic regulatory atlases using cell sorting or gene expression imputation based on single-cell reference panels have offered some improvement, especially for common cell types^8,9,16–18^.

Advances in single-cell and single-nucleus RNA sequencing (RNA-seq) have enabled the collection of transcriptional profiles of diverse cell types and offer an unbiased strategy to study genetic regulatory variants affecting gene expression in each cell type^19,20^. Recent analyses of genetic regulation in single-nucleus transcriptome data from postmortem human brain tissue have identified genetic regulatory effects for broad cell categories, as well as genes, cell types and molecular processes implicated in the biology of brain-related traits^21–23^. However, constructing a genetic regulatory atlas with a larger sample size, greater genetic diversity, a larger number of nuclei and more RNA-seq reads can increase statistical power, cellular resolution and coverage of rare cell types with key roles in disease biology.

In this work, we collected 5.6 million nuclei from the human dorsolateral prefrontal cortex (DLPFC) of a genetically diverse set of 1,384 donors from the full PsychAD dataset, 35.6% of whom are of non-European ancestry^24^. We performed genetic regulatory analyses at two cellular resolutions, with nuclei annotated into eight cell classes and 27 subclasses. Integrating our regulatory atlas with disease risk variants using colocalization analyses identifies cell types and genes underlying the disease biology of brain-related traits. Analysis of *trans*-regulatory effects and dynamic regulatory effects that change over the course of a developmental trajectory offers further insight into the complexity of the genetic architecture of gene expression. This multi-resolution genetic regulatory atlas of gene expression in the human brain improves our understanding of the molecular mechanisms affecting gene expression and disease risk.

## Results

### Genetic regulation of gene expression in the human brain

**Fig. 1:**
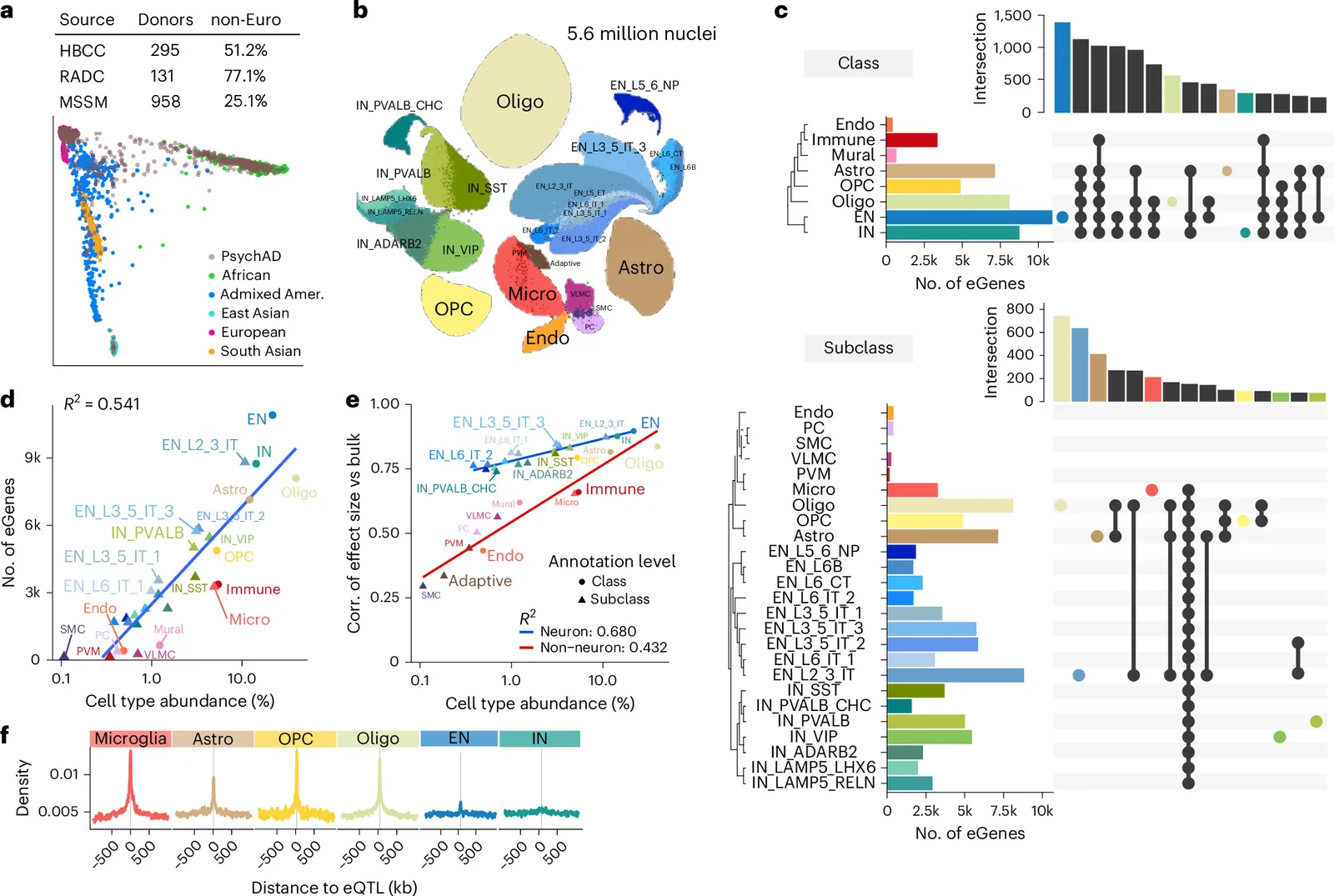
Genetic regulation of gene expression at the cell-type level. **a**, Genetic ancestry of 1,384 donors with genotype data by brain bank. Tissue specimens were obtained from the Human Brain Collection Core (HBCC), Rush Alzheimer’s Disease Center (RADC) and Mount Sinai School of Medicine (MSSM). Principal component analysis of donors from the PsychAD cohort and 1000 Genomes Project shows diverse genetic ancestry (bottom). **b**, UMAP plot of 5.6 million nuclei annotated for 27 cell subclasses. Oligo, oligodendrocytes; Astro, astrocytes; Micro, microglia; Endo, endothelial cells. **c**, Upset plot for the number of eGenes detected for each class (top) and subclass (bottom) along with counts of eGenes detected in multiple classes or subclasses. EN, excitatory neurons; IN, inhibitory neurons; PC, pericytes; SMC, smooth muscle cells; VLMC, vascular and leptomeningeal cells; PVM, perivascular macrophages. **d**, The number of eGenes detected in each class or subclass shown as a function of cell type abundance on a log_10_ scale. Blue line indicates least-squares fit. Squared Pearson correlation is shown. **e**, For each class or subclass, the correlation in estimated genetic effect sizes from bulk-level analysis aggregating all nuclei as a function of cell type abundance on a log_10_ scale. Least-squares fits are shown for neurons (blue) and non-neurons (red). Annotation level is indicated by a circle for class and a triangle for subclass. **f**, Enrichment of OCRs from six matching cell-type populations from single-cell ATAC-seq data^26^ around lead eQTL variants. Vertical line indicates the position of the lead eQTL variant, and the *x* axis shows the distance from this variant. OCR enrichment decays with distance to the lead eQTL variant.

To characterize the genetic regulation of gene expression across cell types in the human brain, we obtained postmortem DLPFC tissue specimens from three brain banks to create a genetically diverse set of 1,384 donors with genotype data from the full PsychAD dataset^24,25^, of which 493 (35.6%) are of non-European ancestry (Fig. 1a and Supplementary Fig. 1). Single-nucleus RNA-seq (snRNA-seq) was performed on postmortem tissue, yielding 5.6 million nuclei after quality control, and nuclei were annotated to eight cell classes and 27 subclasses (Fig. 1b). Analysis of genetic variants within 1 Mb of the transcription start site identified expression quantitative trait loci (eQTLs) at the class and subclass level (Fig. 1c). Genome-wide, eQTL results were highly concordant with results from two single-nucleus datasets and had the highest concordance for matching cell types, with π_1_ ranging from 0.73 to 0.82 for the Bryois dataset^21^, and from 0.84 to 0.91 for the Fujita dataset^22^ (Supplementary Fig. 2). The number of genes with statistically significant eQTLs (that is, eGenes) varied widely, with 10,913 eGenes detected in excitatory neurons, but only 414 in endothelial cells at the class level. Similarly, at the subclass level, 8,812 eGenes were detected in layer 2/3 intratelencephalic excitatory neurons (EN_L2_3_IT) but only 1,683 in layer 6b excitatory neurons (EN_L6B). Analysis at the subclass level increases the cellular resolution, while sacrificing some statistical power to detect effects shared across many subclasses (that is, excitatory neurons). In addition to differences in the genetic regulatory landscape across cell types, statistical power to detect genetic regulatory effects is heavily influenced by other factors. Indeed, the number of detected eGenes increases with cell type abundance (Fig. 1d) and the average read count per cell type (Supplementary Figs. 3 and 4). Furthermore, we evaluated the concordance of genetic effect sizes at the class and subclass level compared to bulk-level analysis aggregating all nuclei. Concordance increased substantially with cell type abundance, with neuronal classes and subclasses showing markedly higher concordance than non-neurons (Fig. 1e), consistent with the higher RNA production and sequencing depth in neurons (Supplementary Figs. 3 and 4). We then conducted fine-mapping to refine the set of candidate causal variants, focusing on *cis*-eQTL loci. Across all significant loci, the median 95% credible set size ranged from ten to 22 variants at the class level and ten to 30 variants at the subclass level (Supplementary Table 1).

Regulatory variants identified in each cell class captured biology specific to that lineage. Cell type-specific open chromatin regions (OCRs) were enriched around lead eQTL variants from the corresponding cell types^26^ (Fig. 1f and Supplementary Fig. 5). This finding is consistent with cell type-specific regulatory programs, especially for glia, with lower enrichment for neurons as seen previously^9,21^. Integrating with a massively parallel reporter assay in human induced pluripotent stem cell-derived NGN2 excitatory neurons^9^, we observed that among the cell types tested, fine-mapped regulatory variants in excitatory neurons showed the strongest association with allelic effect size (Supplementary Fig. 6).

### Cell-type and trait-specific insights into neuropsychiatric and neurodegenerative disorders

**Fig. 2:**
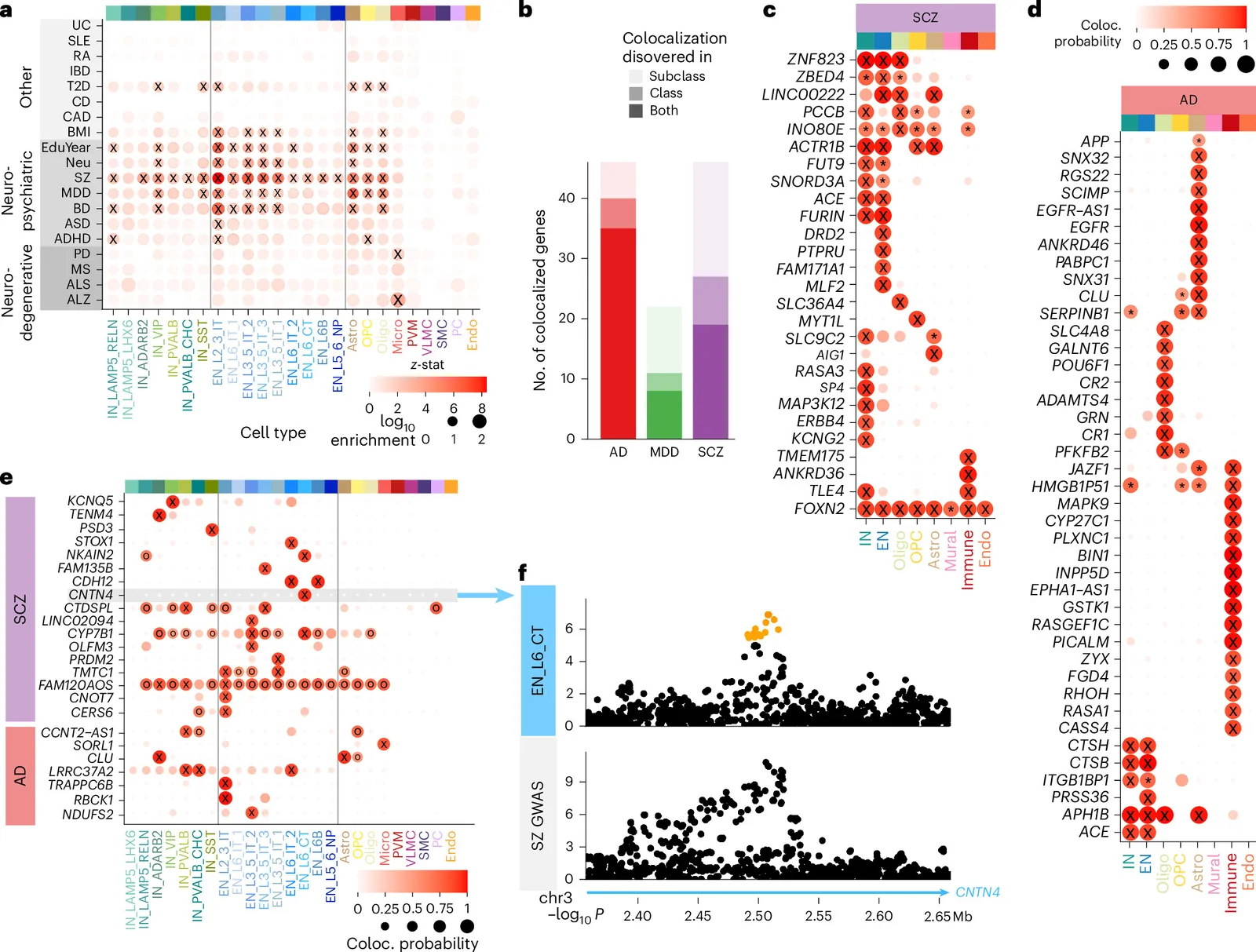
Integrative analysis of variants associated with gene expression and disease risk. **a**, Enrichment of genetic regulatory variants in the 95% credible set from statistical fine-mapping for heritability of genetic traits. Color indicates the *z*-statistic of the null hypothesis of no enrichment; point size indicates log_10_ enrichment. Tests with FDR < 5% are indicated by ‘X’. **b**, Number of genes for which signals for genetic regulation of gene expression and disease risk colocalize with a posterior probability of >0.8. Numbers are shown for cell type class, subclass and intersection. **c**,**d**, Colocalization signal at the class level for SCZ (**c**) and AD (**d**). Posterior probabilities of >0.8 are indicated by ‘X’, and >0.5 are indicated by ‘*’. **e**, Colocalization signals at the subclass level for SCZ and AD that are not found at the class level. Posterior probabilities of >0.8 are indicated by ‘X’, and >0.5 are indicated by ‘o’. **f**, Manhattan plot of colocalization between regulation of *CNTN4* expression in astrocytes and genetic risk for SCZ. Color indicates posterior inclusion probability from statistical fine-mapping.

Integrating this catalog of genetic regulatory variation with genetic risk for complex traits can identify genes and cell types underlying disease biology. Pairs of cell types and traits for which regulatory variants from statistical fine-mapping were enriched for trait heritability were identified using stratified linkage disequilibrium score regression (S-LDSC) after accounting for baseline annotations^3^ (Fig. 2a and Supplementary Fig. 7). Neuronal regulatory variants are enriched for heritability for neuropsychiatric traits, with schizophrenia (SCZ) showing the broadest enrichment, followed by bipolar disorder and major depressive disorder (MDD). Yet these traits are also enriched in astrocytes and oligodendrocytes, and SCZ and MDD also show enrichment in oligodendrocyte precursor cells (OPCs). The neurodegenerative traits Alzheimer’s disease (AD) and Parkinson’s disease show enrichment in microglia but not in neuronal subclasses. In analysis at the class level, where there is increased power to detect effects shared across many subclasses, Parkinson’s disease, multiple sclerosis and amyotrophic lateral sclerosis show enrichment in neurons (Supplementary Fig. 7). Other complex traits examined do not show enrichment for brain regulatory variants, with the exceptions of type 2 diabetes and body mass index, which are metabolic traits with a behavioral component. In addition, heritability mediation analysis^27^ finds that regulatory variants in specific cell types also mediate a significant fraction of the heritability for complex traits, with notable signals for SCZ in neurons and AD in microglia (Supplementary Fig. 8).

Colocalization analysis of genetic signals shared between regulatory and risk variants identified genes involved in the molecular etiology of disease, including 46 genes in AD^28^, 22 in MDD^29^ and 46 in SCZ^30^ (Fig. 2b). Notably, up to 18 of these genes were exclusively detected in snRNA-seq analyses and were not captured by previous bulk RNA-seq studies (Supplementary Fig. 9). Evaluating these colocalization signals in an independent dataset showed similar replication rates for signals at the class and subclass levels (Supplementary Fig. 10). Although some genes are shared across class-level and subclass-level analyses, many are only identified at one level, highlighting the importance of multi-resolution analyses. At the class level, many of the colocalized genes for SCZ are identified only in excitatory and inhibitory neurons, such as *FUT9*, *SNORD3A*, *ACE* and *FURIN*, whereas others, like *ACTR1B* and *ZNF832*, are also shared with other cell types (Fig. 2c). *DRD2*, *PTPRU*, *MLF2* and *FAM171A1* are only identified in excitatory neurons, while *RASA3*, *SP4*, *MAP3K12*, *ERBB4* and *KCNG2* are only identified in inhibitory neurons.

Colocalization analysis for AD identifies key cell types sharing regulatory and disease risk signals. The role of microglia in genetic mechanisms of AD biology is well established, with 16 genes showing a colocalization signal in immune cells, driven largely by microglia (Fig. 2d). However, nine genes are identified in oligodendrocytes, 12 in astrocytes and six in neurons at the class level, with very limited overlap between cell types. This highlights molecular mechanisms in addition to those involving microglia in AD etiology. Microglial genes were enriched for pathways involved in amyloid-β formation and APP processing, consistent with the established role of microglia in plaque biology. Excitatory and inhibitory neurons were enriched for thyroid hormone response and peptide catabolic processes, suggesting neuron-intrinsic regulatory mechanisms influencing amyloid metabolism. Oligodendrocytes were enriched for complement activation and humoral immune response pathways, highlighting a potential immune-regulatory role for oligodendrocytes in AD pathogenesis (Supplementary Fig. 11).

Analysis at the higher-resolution subclass level identifies additional genes that are not found at the class level (Fig. 2e and Supplementary Fig. 12). These genes tend to show colocalization signals in subsets of neurons, given that they were missed by collapsing diverse neuronal subclasses into only excitatory and inhibitory neuron classes. For example, *CNTN4* coding for the contactin 4 protein involved in cell adhesion only colocalized with SCZ risk in layer 6 corticothalamic excitatory neurons (EN_L6_CT) (Fig. 2f). Similarly, *SORL1* coding sortilin related receptor 1 colocalized with AD risk in microglia but not perivascular macrophages at the subclass level or the lower resolution ‘immune’ cell type at the class level (Supplementary Fig. 13). We also note eight loci at the class level and 11 at the subclass level that contain multiple genes with colocalization signals within 1 Mb (Supplementary Table 2).

### Cell type specificity of genetic regulatory effects reveals distinct mechanisms in neurodegenerative disease risk

Diverse cell types have key roles in health and disease, and characterizing differences in genetic regulatory effects at higher resolution can offer insight into the distinct functions of these cell types. Although the cell type specificity of regulatory and disease biology is widely appreciated, identifying a cell type-specific regulatory effect of a genetic variant in a statistically rigorous manner is challenging. Simply detecting a significant association between a genetic variant and the expression of a gene in one cell type but not another does not mean that the biological effect is specific to the first cell type. This dilemma is common when statistical power is limited, or when there is a substantial difference in power between cell types. Indeed, widely used frequentist statistics are inadequate to address this important question^31–33^.

**Fig. 3:**
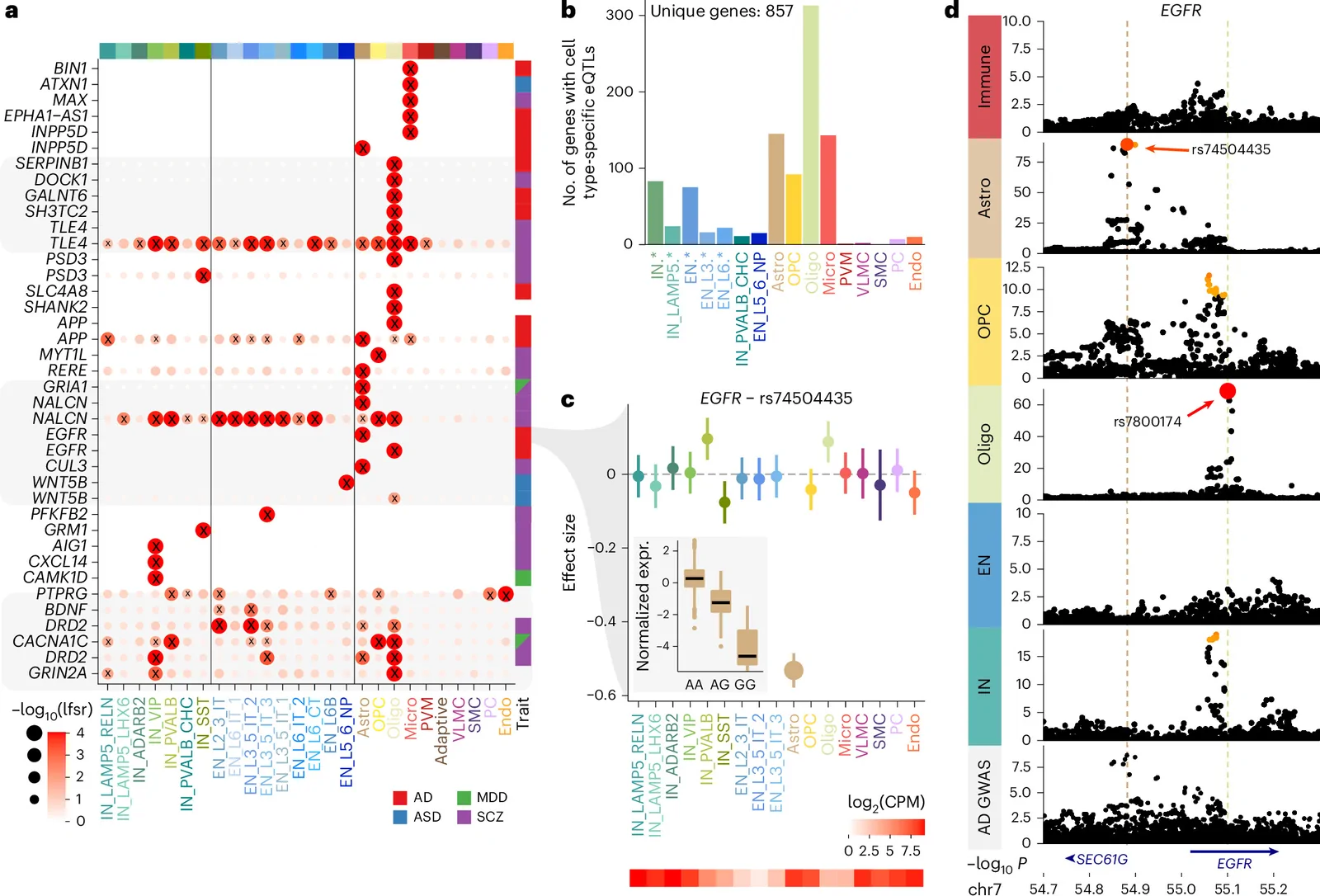
Cell type specificity of genetic regulatory effects. **a**, Cell type-specific and shared genetic effects for a set of genes across each subclass. The posterior probability that the effect of the lead genetic variant is non-zero for each gene is indicated by the −log_10_ local false sign rate (lfsr). Genes shown twice have independent regulatory signals in different cell types. Genes were selected based on cell type specificity, except that the bottom six genes were selected based on biological interest. The right column indicates genetic colocalization between eQTL and GWAS for the specified trait. For example, *EGFR* has independent signals in astrocytes and oligodendrocytes, while *INPP5D* has independent signals in astrocytes and microglia. ASD, autism spectrum disorder. **b**, Number of genes with cell type-specific effects detected in each subclass or set of subclasses. Entries such as “IN.*” indicate a set of all subclasses with that prefix, and the count corresponds to the number of genes with a cell type-specific effect in at least one member of the set. **c**, Estimated effect size for variant rs74504435 on EGFR expression. Error bars indicate 95% confidence intervals. Heatmap (bottom) shows expression level in each subclass. CPM, counts per million. Inset graph shows expression stratified by this variant in astrocytes. Boxes indicate mean, 25th and 75th percentile, and bars indicate 1.5× the interquartile range. **d**, Manhattan plot for *EGFR* showing cell type specificity for two genetic variants. Only the signal in astrocytes colocalized with genetic risk for AD. Color indicates posterior inclusion probability from statistical fine-mapping. *P* values for QTL analysis are from a two-sided *z*-test from a linear mixed model.

We apply a multivariate Bayesian meta-analysis to produce posterior estimates of the eQTL effect size and the posterior probability that each genetic effect is non-zero^33^. This approach shrinks effect size estimates across genes and cell types to be more robust to differences in statistical power. Examining genes with eQTLs detected in a single cell type by this Bayesian approach and intersecting with colocalization results highlights specific regulatory genetics and their role in disease biology (Fig. 3a). For example, examining colocalization with AD shows genetic regulatory variants for *BIN1* and *EPHA1-AS1* are only detected in microglia, and for *SERPINB1* and *GALNT6*, only in oligodendrocytes. The key AD gene *APP*, encoding the amyloid precursor protein, has a genetic regulatory signal specific to oligodendrocytes as well as another signal in astrocytes shared with other cell types. Other genes have separate eQTL signals detected in distinct cell types. *INPP5D* has an eQTL signal detected only in microglia and a separate signal detected only in astrocytes, while *EGFR*, *PSD3*, *NALCN*, *TLE4* and *WNT5B* each have separate eQTL signals detected in distinct cell types (Supplementary Fig. 14).

Using a novel composite hypothesis approach, we can directly estimate the posterior probability that a genetic regulatory effect is specific to a given cell type. Analysis at the subclass level identified 857 unique genes with cell type-specific effects at posterior probability > 0.5, with 981 at the class level (Fig. 3b and Supplementary Fig. 15). At the subclass level, oligodendrocytes have the most cell type-specific genetic regulatory effects with 313 genes, followed by astrocytes with 145 and microglia with 143. Using stricter posterior probability cutoffs at 0.8 retains cell type-specific effects: 630 at the class level and 644 at the subclass level (Supplementary Fig. 15). Although cell types with the most cell type-specific findings are biologically distinct from other subclasses, neurons comprise multiple similar subclasses and show less cell type specificity. We performed additional composite testing to identify genetic effects present in at least one constituent cell type.

The *EGFR* gene encoding the epidermal growth factor receptor has a complex pattern of cell type-specific genetic regulation and colocalization with disease risk. The gene has at least two separate eQTL signals in astrocytes and oligodendrocytes. The lead variant for the astrocyte signal is rs74504435, which has a composite posterior probability of 0.946 that it is associated only with *EGFR* expression in astrocytes (Fig. 3c). This genetic regulatory signal in astrocytes colocalizes with risk for AD, whereas the regulatory signal in oligodendrocytes is not associated with AD risk, highlighting the cell type-specific role of disease biology (Fig. 3d).

### Dynamic genetic regulation across neurodevelopment identifies shifting eQTL effects and links to disease risk

**Fig. 4:**
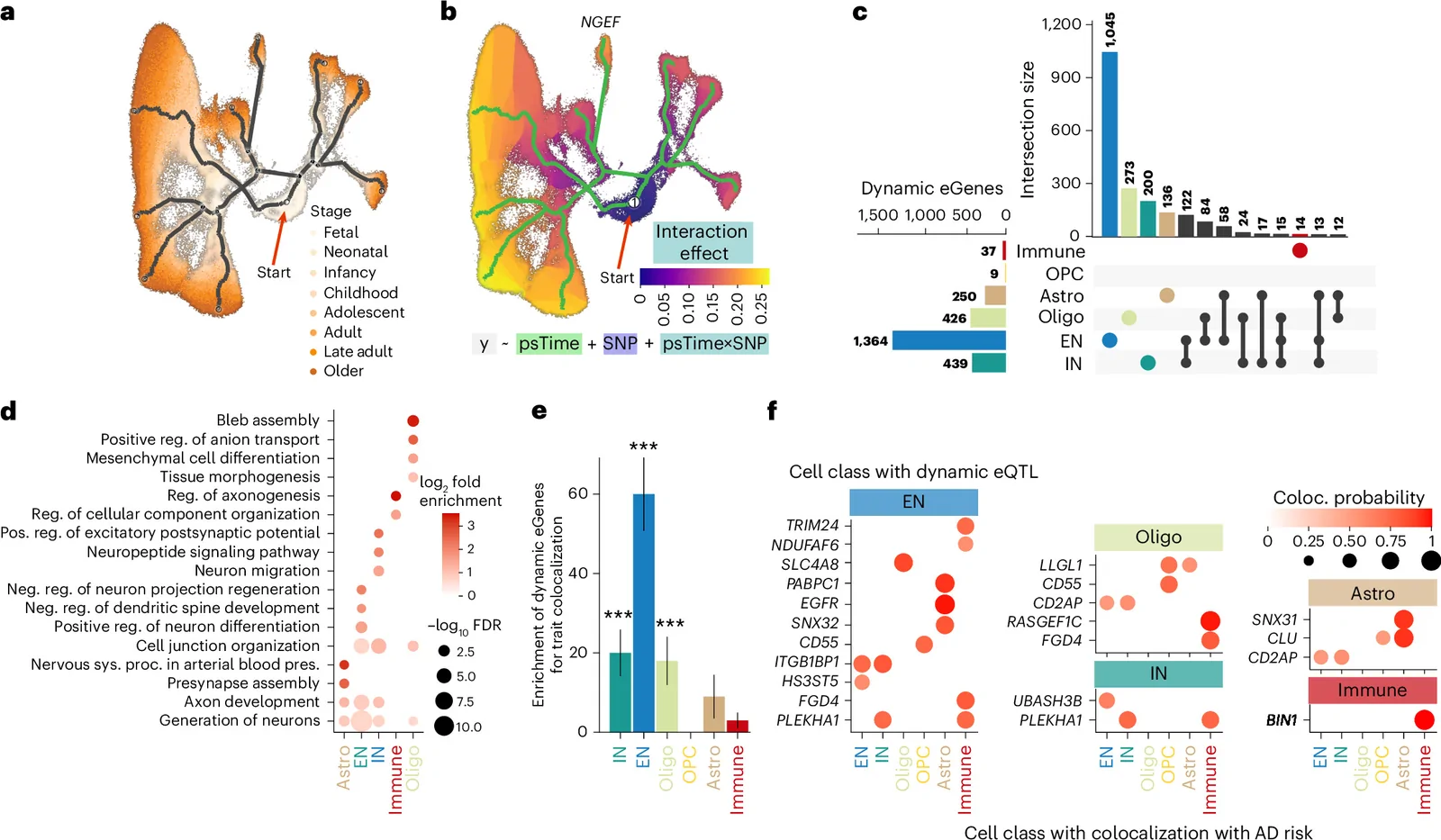
Dynamic genetic regulatory effects vary over an aging trajectory. **a**, Supervised aging trajectory in excitatory neurons, with nuclei colored by developmental stage. Gray lines indicate the pseudotime trajectory, and the red arrow indicates the beginning of pseudotime. **b**, Supervised aging trajectory in neurons from **a**, with cells colored by the dynamic genetic effect for *NGEF* changing over the pseudotime trajectory. The equation presents a regression model of gene expression (*y*), pseudotime (psTime), genetic variant (SNP) and the interaction term (psTime × SNP), capturing how the genetic effect changes with pseudotime. **c**, Number of dynamic eQTLs detected in each cell class, along with intersection across classes. **d**, Gene set enrichment for genes with dynamic eQTLs detected in each cell class. **e**, Dynamic eGenes are enriched for genes colocalized with complex traits. Bars indicate estimated enrichment, and error bars indicate 95% confidence intervals. ‘***’ indicates *P* values that pass the multiple-testing cutoff of 5%. **f**, Genes with a dynamic eQTL (shown in the inset of each cell class) that have a colocalization signal for AD genetic risk (shown on the *x* axis). For example, the gene *EGFR* has a dynamic eQTL detected in EN, and regulatory variants for this gene colocalize with AD risk in astrocytes. The color and size of the circle indicate posterior probability from colocalization analysis. A colocalization cutoff of PP4 > 0.5 is used, so some genes that were filtered out above were included here.

Neurodevelopment is a key biological process in the etiology of brain-related traits, and gene expression in some cell types changes substantially over developmental time. We reasoned that genetic regulatory effects on gene expression also change over developmental time. Subsetting the full PsychAD dataset^24^, a previous study^34^ extracted a neurotypical aging cohort of 284 postmortem donors aged 0–97 years, comprising 1.3 million nuclei, and constructed a pseudotime trajectory for each cell type using a supervised method incorporating donor age^35^. The trajectory for each cell type is anchored at early development and extends toward adulthood, with each nucleus assigned a continuous pseudotime value (Fig. 4a). Dynamic eQTL effects for each cell type were detected by testing whether the effect size of a genetic variant on the expression of a given gene changes along this trajectory. Analysis was performed using a negative binomial mixed model to consider multiple nuclei from each donor and account for overdispersion of observed counts. For example, in excitatory neurons, the genetic effect of rs1878289 on the expression of *NGEF*, encoding a neuronal guanine nucleotide exchange factor, increases substantially during cellular maturation (Fig. 4b). A total of 2,073 unique genes with dynamic eQTLs were detected at a false discovery rate (FDR) of 5%, with the number varying widely from 1,364 in excitatory neurons to only nine in OPCs, with the highest overlap between excitatory and inhibitory neurons (Fig. 4c). This finding is consistent with the extensive developmental dynamics and cellular diversity of excitatory neurons compared to the relative homogeneity of OPCs^36^. Genes with dynamic eQTLs are enriched for developmental processes such as the generation of neurons and cell junction organization across multiple cell types (Fig. 4d). Astrocytes show enrichment for nervous system processes related to arterial blood pressure, consistent with their key role in angiotensin production^37^. Excitatory neurons show enrichment for neuronal development and differentiation, while inhibitory neurons show enrichment for neuron migration and microglia show enrichment for axonogenesis. Oligodendrocytes are enriched for genes involved in bleb assembly, an important morphological and migratory process^38^.

Genes with dynamic regulatory signals detected in excitatory neurons, inhibitory neurons and oligodendrocytes are enriched for genes with disease colocalization signals identified above, underscoring the importance of regulatory dynamics in disease biology (Fig. 4e). Genes often have a dynamic regulatory signal detected in one cell type and a disease colocalization signal detected in a different cell type. We observe this for AD (Fig. 4f), SCZ, MDD and autism spectrum disorder (Supplementary Figs. 16 and 17). This effect can be attributed to differences between the regulatory architecture driving dynamic changes in gene expression over aging and genetics affecting steady-state gene expression, differences in aging trajectories across cell types and differences in statistical power across cell types. Meanwhile, six genes have a dynamic regulatory signal and disease colocalization signal detected in the same cell class (Supplementary Fig. 18). *CLU*and *SNX31* have a dynamic regulatory signal and colocalization signal with AD in astrocytes, *ACTRB* and *FAM171A1* with SCZ in excitatory neurons, *BIN1* with AD in immune cells and *NEGR1* with MDD in oligodendrocytes.

### *Trans*-eQTL mapping identifies brain cell type-specific genetic regulatory hubs and links to disease risk

**Fig. 5:**
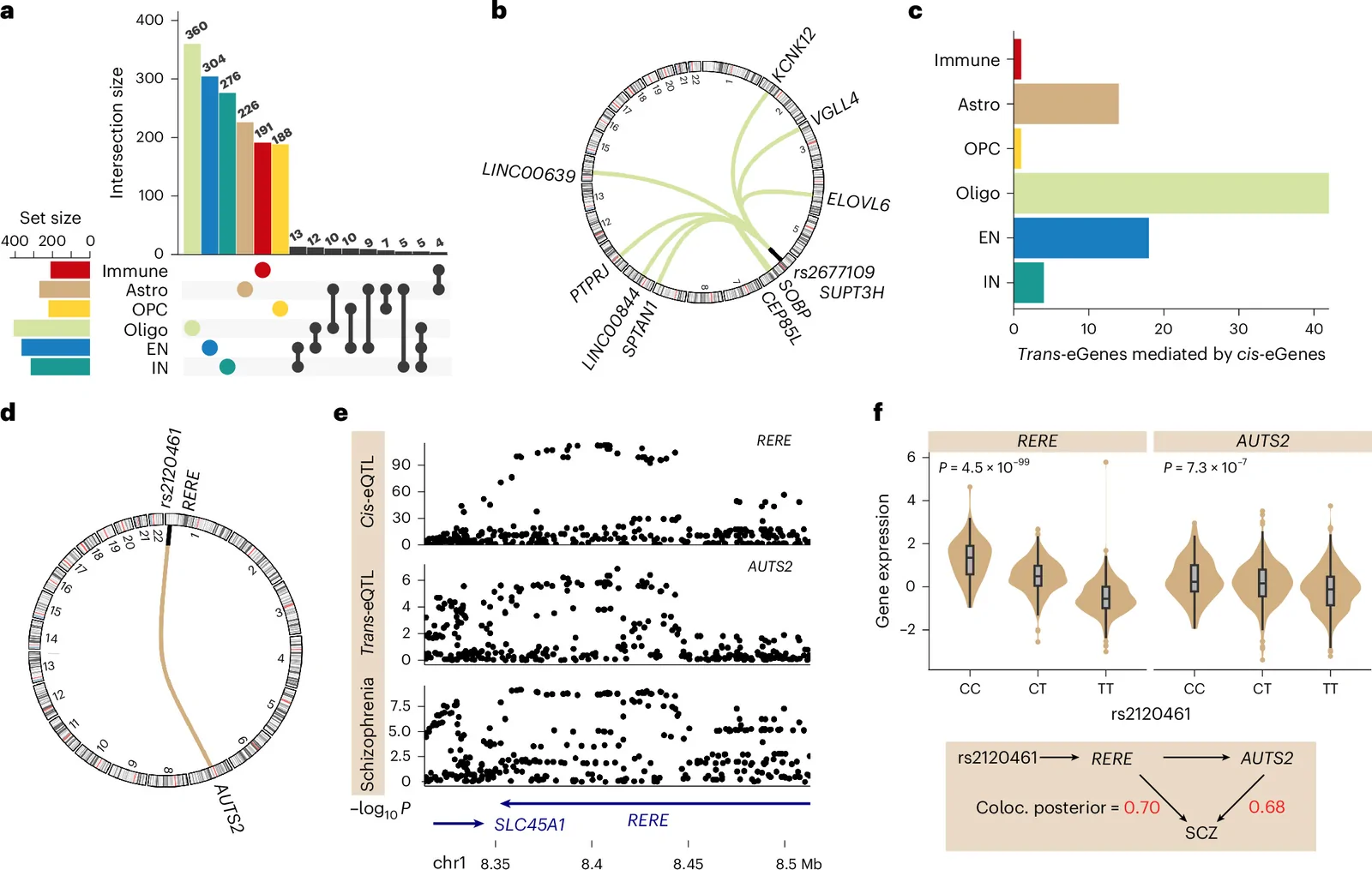
Analysis of *trans-*regulatory signals. **a**, Number of genes with *trans*-eQTLs detected in each cell class, and the overlap between classes. **b**, Circos plot of *trans*-eQTL hub showing variant rs2677109 on chr6 associated with *cis*-gene *SUPT3H* and with nine *trans*-genes. **c**, Number of genes for which statistical mediation analysis supports the *cis*-eQTL mediating the *trans*-regulatory signal at an FDR of 5%. **d**, Circos plot of *cis* association of rs2120461 and expression of *RERE*, and *trans* association with *AUTS2*. **e**, Manhattan plot showing shared genetic signal from *cis*-regulation of RERE, *trans*-regulation of AUTS2 and genetic risk for SCZ. *P* values for QTL analysis are from a two-sided *z-*test from a linear mixed model. **f**, Expression of *RERE* and *AUTS2* in astrocytes stratified by rs2120461, with *P* values from eQTL regression analysis (top). Boxes indicate the mean, 25th and 75th percentile, and bars indicate 1.5× the interquartile range. Proposed regulatory model in which a genetic regulatory variant in linkage disequilibrium with rs2120461 drives expression variation in *RERE*, *AUTS2* in astrocytes and SCZ risk (bottom). Colocalization posterior probability with SCZ risk is indicated in red. *P* values for QTL analysis are from a two-sided *z*-test from a linear mixed model.

Genetic variants located outside the local *cis*-regulatory window of a gene can exert significant influence on gene expression through *trans*-regulatory mechanisms. Analysis of *trans*-regulatory signals in each cell class identifies 1,655 unique genes with *trans*-eQTL signals >5 Mb from the gene body at a study-wide FDR of 5%. The number of *trans*-eGenes varied by cell type, ranging from 407 in oligodendrocytes to 210 in immune cells, with limited overlap across cell types (Fig. 5a). Analysis of these *trans*-eQTL discoveries in the independent Fujita^22^ cohort showed estimated replication rates from 66% to 95%, depending on cell type (Supplementary Fig. 19). Analysis of genetic variants associated with multiple *trans*-eGenes identified four *trans*-regulatory hubs centered at three transcriptional regulators (SUPT3H, RUNX2 and ZNF160), each influencing at least three target genes, with the largest hub regulating nine downstream targets in oligodendrocytes (Fig. 5b and Supplementary Fig. 20). Intersecting *trans*-eGenes with genes exhibiting *cis*-regulatory signals that colocalize with disease risk, we identify 12 genes with colocalization probabilities of >0.8 and 32 with colocalization probabilities of >0.5 (Supplementary Fig. 21 and Supplementary Table 3).

To further examine the regulatory mechanisms underlying *trans*-eQTL signals, we performed genetic mediation analysis to statistically test whether the *trans*-eQTL effect is indeed mediated by regulation of a *cis*-gene. Although analysis was underpowered to detect a large number of *cis*-mediators at the study-wide significance threshold of a 5% FDR, using Storey’s π_1_ method^39^, we estimate that 43% of *trans* signals are mediated by *cis-*genes, with detection primarily limited by statistical power. Nonetheless, we identified 42 *trans-*eQTLs mediated by *cis*-genes in oligodendrocytes, with fewer detected in other cell classes (Fig. 5c).

In astrocytes, mediation analysis supports the hypothesis that the *trans*-eQTL signal between rs2120461 on chromosome 1 and expression of *AUTS2* on chromosome 7 is mediated by a *cis*-regulatory effect on expression of *RERE* (FDR = 1.5 × 10^−3^) (Fig. 5d). This *trans*-eQTL and mediation are replicated in the independent Fujita^22^ dataset (Supplementary Fig. 22). Both *RERE* and *AUTS2* are implicated in neurodevelopmental and neuropsychiatric disorders^40,41^. *RERE* is a transcriptional regulator involved in retinoic acid signaling in early development^42^, and *AUTS2* has a role in neuronal differentiation^43^. Moreover, colocalization analysis revealed that the *cis* regulation of *RERE* and the *trans* regulation of *AUTS2* colocalize, albeit weakly, with the genetic risk for SCZ (Fig. 5e). This observation suggests a mechanistic model in which the genetic regulation of *RERE* and its downstream effect on *AUTS2* in astrocytes contribute to SCZ susceptibility (Fig. 5f).

## Discussion

The brain is composed of a diverse array of cell types with distinct biology, gene expression patterns, genetic regulatory architecture and roles in development and disease. Genetic risk variants for complex traits largely act by modifying gene expression; however, our understanding of cell type-specific regulatory variation and its role in disease has been limited by sample size and cell type resolution. Here, we present a multi-resolution atlas of genetic regulation in the human brain, spanning eight cell classes and 27 subclasses, generated from 5.6 million single nuclei obtained from 1,384 donors of diverse ancestry. We identify *cis*-eQTLs for 14,258 genes and observe wide variation in the number of *cis*-QTLs detected for each cell class and subclass owing to differences in cell type abundance. This finding highlights the importance of increasing sample size to study the regulatory architecture of rarer cell types. The regulatory signals are distinct across cell types and, for non-neuronal cells, reflect chromatin accessibility in each cell type.

Integration with GWAS for brain-related traits identifies cell types mediating genetic risk for each trait. Colocalization analysis further identifies specific genes and cell types mediating genetic risk that add insight into disease biology. The role of neurons in SCZ is well established^30^, but the role of neuron subtypes is not well understood. As a step towards a higher resolution understanding, we identified multiple genes colocalizing with SCZ risk in specific neuronal subtypes. Although the role of genetic regulation of *CNTN4* expression in SCZ was first identified from bulk gene expression profiling^12^, we find a shared regulatory and SCZ risk signal only in layer 6 corticothalamic excitatory neurons.

Recent work in AD has uncovered the unique role of microglia, brain-resident myeloid cells, in genetic risk and molecular etiology^28,44^. In addition to identifying genetic regulatory signals shared with AD risk in microglia, we also identify genes that are not detected in microglia. Among others, these include well-studied genes such as *APP*, *SNX31*, *SNX32*, *EGFR* and *CLU* in astrocytes; *CR1*and *CR2* in oligodendrocytes; and *CTSB*, *CTSH* and *ACE* in neurons.

Identifying cell type-specific regulatory effects, in which a genetic effect is non-zero in only a specified cell type, is challenging with existing frequentist methods. Building on Bayesian multivariate meta-analysis, we prioritize genes based on their specific versus shared regulatory signals and examine the complex regulatory architecture that is active in specific cell types. We highlight the example of *EGFR*, which has at least two distinct regulatory programs; one active in astrocytes and another in oligodendrocytes. Notably, only the regulatory signal in astrocytes colocalizes with the genetic risk of AD, highlighting the importance of cell type-specific regulatory programs in disease biology.

Early developmental processes have a key role in neurodevelopmental and neuropsychiatric disease^45^. Yet studying the genetic regulatory architecture at cell-type resolution at this key stage is especially challenging. Here, we leverage the wide age span of donors in this dataset and integrate with a neurotypical aging cohort from the PsychAD to construct a pseudotime trajectory within each cell class. We identify dynamic eQTLs by testing for genetic effects that vary over developmental time. Dynamic genetic effects are most prevalent in excitatory and inhibitory neurons, and dynamic eGenes in these classes are enriched for colocalization with brain-related traits.

The unique scale of this dataset enabled the discovery of *trans*-eQTLs for the most abundant cell classes. We find limited overlap between cell classes, and we identify *trans*-eGenes that also have a *cis*-regulatory signal colocalizing with disease risk. In astrocytes, we identify a *cis*-eQTL for *RERE* as a *trans*-eQTL for *AUTS2*, and both regulatory signals colocalized with genetic risk for SCZ, underscoring the role of complex *trans*-regulatory architecture in disease biology.

Although single-cell and single-nucleus transcriptomics enable high-resolution mapping of cell type-specific expression, the power to detect eQTLs is strongly influenced by cell-type abundance and, to a lesser extent, by sequencing depth. Owing to the wide variability in cell-type proportions, major cell classes, such as inhibitory neurons, excitatory neurons, oligodendrocytes and astrocytes, exhibit a higher number of detectable eQTLs than rarer populations, including immune, endothelial and mural cells. Further stratification into 27 cell subclasses enhances cellular resolution but often reduces statistical power relative to class-level analyses because of decreased sample sizes within each subclass. This trade-off between cellular granularity and statistical power represents a critical consideration when designing and interpreting single-cell eQTL studies.

Our findings provide key insights into the cell type-specific genetic regulation underlying neuropsychiatric and neurodegenerative diseases. This study underscores the importance of expanding sample sizes and increasing single-cell resolution to capture rarer cell types, paving the way for a deeper understanding of disease mechanisms. As we move forward, integrating multi-omic data and ensuring representation of diverse ancestries will be critical to advancing precision medicine and developing targeted therapeutic strategies for brain disorders.

## Methods

All procedures and research protocols were approved by the Institutional Review Board (IRBs) of Rush University Medical Center and the Mount Sinai and Mount Sinai/James J. Peters VA Medical Center. The autopsied brain specimens originated from brain donation programs at Rush University Medical Center/Rush Alzheimer’s Disease Center and the Mount Sinai Brain Bank, including samples collected from the James J. Peters VA Medical Center National Institutes of Health (NIH) Brain and Tissue Repository. All research conformed to the principles of the Declaration of Helsinki. Participants did not receive compensation. Sample numbers were determined by the availability of fresh brain autopsies. No statistical methods were used to predetermine sample size

### Sample selection and preprocessing

Brain tissue from the DLPFC was obtained from 1,494 donors by the PsychAD Consortium^24,25^. The dataset comprised donors from three sources. The Rush Alzheimer’s Disease Center repository of tissue from the Religious Orders Study or Rush Memory and Aging Project^46^ provided 152 specimens; the Human Brain Collection Core provided 300; and the Mount Sinai Brain Bank (Mount Sinai School of Medicine) provided 1,042 specimens. The cohort includes similar numbers of males and females and spans the entire postnatal age range of 0 to 108 years. See previously published work^24,25^ for additional details about the donors and data processing.

Paired-end reads from snRNA-seq libraries were aligned to the hg38 reference genome using STAR solo^47^, and sample pools were demultiplexed through genotype matching with Vireo (v0.5.8)^48^. Following the generation of per-library count matrices, downstream processing was conducted using Pegasus (v.1.7.0)^49^ and scanpy (v.1.9.1)^50^.

We implemented a stringent quality control process to eliminate ambient RNA and preserve high-quality nuclei for further analysis. As part of our rigorous quality control pipeline, we initially tested for potential contamination using CellBender (v0.4.0)^51^, a deep generative model specifically designed to remove counts owing to ambient RNA molecules and random barcode swapping. In our initial assessment, however, we did not observe significant ambient RNA contamination across our datasets. In addition, to ensure the highest fidelity of the final processed object and to robustly confirm that our cell-type specificity results were not driven by background noise, we additionally performed an analysis using SoupX (1.6.2)^52^. This secondary validation confirmed that our data are free of significant ambient RNA effects.

Genomic DNA was extracted from frozen brain tissue using the QIAamp DNA Mini Kit (Qiagen), following the manufacturer’s instructions. The samples were genotyped using the Infinium Psych Chip Array (Illumina) at the Mount Sinai Sequencing Core. Pre-imputation processing involved running the quality control script HRC-1000G-check-bim.pl from the McCarthy Lab Group, using the Trans-Omics for Precision Medicine (TOPMed) program. Genotypes were phased and imputed on the TOPMed Imputation Server (https://imputation.biodatacatalyst.nhlbi.nih.gov). Samples were excluded if there was a mismatch between self-reported and genetically inferred sex, suspected sex chromosome aneuploidies, high relatedness (KING^53^ kinship coefficient of >0.177) or outlier heterozygosity (±3 s.d. from the mean). Additionally, samples with a sample-level missingness of >0.05 were excluded, as calculated within a subset of high-quality variants (variant-level missingness of ≤0.02). A total of 1,384 donors with genotype data and snRNA-seq data passing quality control were analyzed in this study.

#### Cell annotation

Cell annotations of the PsychAD dataset at the class and subclass level are provided in a companion paper^24^. Cellular taxonomy was defined using a divide-and-conquer strategy. From the full PsychAD dataset containing over six million nuclei, eight major cell classes were defined using the following steps: 6,000 highly variable genes (HVGs) were selected from mean and dispersion trends^54^ using default parameters (min_mean = 0.0125, max_mean = 3, min_disp = 0.5) and brain source as a batch variable after manually excluding sex and mitochondrial chromosomes. We used the *k*-nearest neighbor (kNN) graph calculated based on a harmony-corrected principal component analysis embedding space to cluster nuclei of the same cell type using the Leiden^55^ clustering algorithm. We used uniform manifold approximation and projection (UMAP)^56^ to visualize the resulting clusters. From the class-level clusters, we subsetted the data by each class. Re-calculating HVGs among cells in the same class allowed us to re-focus on a feature space that is more relevant for the same class of cells. A kNN graph was then calculated based on the harmony-corrected principal component analysis of the selected HVGs. Leiden clustering was used to annotate 27 subclass-level annotations. We iterated the same HVG–kNN–Leiden clustering for all 27 subclasses, yielding 67 subtypes of human brain cells. After obtaining annotations at three levels of hierarchy, the resulting clusters were aggregated into pseudobulk profiles, and cluster-wise Pearson correlation coefficients were calculated using existing human DLPFC^57^ and M1 (ref. ^58^) annotations. We matched the annotations based on both high correlation and specificity of the cell type.

#### Normalization of gene expression

Pseudobulk read counts were calculated by summing reads from the same individual using the dreamlet workflow^59^. As done in the companion paper^24^, we performed variance partitioning analysis to identify variables correlated with gene expression. This identified the proportion of mitochondrial expression as an important variable; it was regressed out to mitigate technical variability linked to batch effects, cell quality and potential stress-related artifacts. In addition, the effects of the sample pool and the influence of disease status were also controlled for by regression. Finally, the residuals were divided by the predicted standard deviation to produce Pearson residuals, excluding the impact of varying sequencing depths among the scRNA-seq libraries.

We applied the PEER package^60^ to detect hidden unobserved covariates. To find an optimal number of PEER factors to remove, we performed eQTL detection on the input expression matrix, normalized by pre-selected biological and technical covariates, varying the number of PEER factors from ten to 98 in increments of four. The setting with the most genome-wide significant eQTLs detected was used as the final result for downstream analysis (Supplementary Fig. 23).

#### Analysis of genetic regulatory variants at the pseudobulk level

Analysis was performed at the pseudobulk level. For each cell type, donors with at least five nuclei were retained in order to ensure stable donor-level expression estimates and to reduce noise from sparse sampling. Genes were filtered within each cell type to retain only those that were robustly expressed. This filtering procedure was implemented using edgeR::filterByExpr() and required that at least 40% of donors have a minimum count of five reads per gene. After quality control and filtering, the number of donors retained for each cohort varied (Supplementary Table 4).

The regression residuals used in the eQTL analysis were generated as follows. Expression for each cell type and each individual was computed as the log_2_ counts per million with a pseudocount of 0.25 after aggregating all corresponding nuclei. A precision-weighted regression model was fit for each expressed gene in each cell type with the dreamlet package (v1.4.1)^59^ using covariates for age, sex, postmortem interval, mitochondrial rate, ribosomal rate and disease status. From these models, Pearson residuals (that is, residuals divided by their standard errors) were computed for each expressed gene and cell type and used in downstream QTL analysis. Using the Pearson residuals identified 3.9–10.5% more genome-wide significant eGenes than using raw residuals (Supplementary Fig. 24).

In each cell class and subclass, eQTL analysis was performed for variants within 1 Mb of the transcription start site of each expressed gene. Imputed variants were filtered based on genotype completeness (95%), minor allele frequency (1%) and standard quality metrics (imputation INFO of >0.3) to ensure high-confidence association testing. Donors were required to pass quality control in both genotype and expression datasets. Given the diverse genetic ancestry of the individuals in this dataset, we used a linear mixed model to account for population structure and avoid false-positive findings resulting from genetic confounding^61^. We implemented this process using the mmQTL software (v1.5.0)^7^ to model a genetic relatedness matrix between all pairs of individuals as a random effect. *Cis*-eQTL analyses were performed exclusively on autosomal chromosomes. Variants and genes located on the X chromosome were excluded. Each cohort was analyzed separately, and eQTL results were combined using a fixed-effects meta-analysis.

Multiple-testing correction was performed using a two-step Benjamini–Hochberg FDR control framework, as done previously^7^. First, for each gene, all tested variants within the *cis* window were adjusted using the Benjamini–Hochberg procedure to control the FDR across variants tested for that gene. Second, we applied an across-gene correction by applying the Benjamini–Hochberg procedure across genes to control the genome-wide FDR.

#### Replication in independent cohorts

For the ROSMAP cohort of 424 donors and 1.5 million nuclei from the DLPFC, raw snRNA-seq counts were obtained from the Fujita^22^ dataset. Alignment, quality control, cell type annotation and eQTL analysis were performed as for the PsychAD data. The cell type composition is similar to the PsychAD cohort (Supplementary Fig. 22).

The Bryois dataset^21^ comprised three cohorts including 192 donors and 750,000 nuclei from the prefrontal cortex, temporal cortex and white matter across eight annotated cell types. They provided eQTL summary statistics that we used in our replication analysis.

#### Evaluating replication of genetic regulatory variants across datasets

We used the R package qvalue to estimate eQTL replication rates using Storey’s π_1_ statistic^39^. For a pair of datasets, we first extracted the most significant variant for genes with an eQTL in the discovery data. The *P* values from the replication dataset were then used to estimate Storey’s π_1_ value, which indicates the fraction of hypothesis tests for which the null is rejected. Thus, π_1_ is the estimated fraction of eQTLs that replicate in the second dataset. This metric of replication is useful because it does not depend on hard cutoffs for *P* values for FDR, and it has been widely adopted. The PsychAD cohort from the current study was used for discovery, and replication rates were evaluated using independent snRNA-seq data of human postmortem brain tissue from the Bryois^21^ and Fujita^22^ datasets.

#### Enrichment of OCRs around detected regulatory variants

To determine whether cell type-specific regulatory elements were enriched around eQTLs, we used the fdensity function in QTLtools (v1.3.1)^62^ to compute the number of functional elements that overlap each 10 kb bin in a 2 Mb window around the *cis*-eQTL. The OCR annotations were obtained from single-cell ATAC-seq data from human brain tissue^26^, and cell type-specific OCRs were defined as those found in only one cell type.

#### Fine-mapping of *cis*-eQTLs

We conducted fine-mapping with CAVIAR^63^ (v.2.0.0), which implements a probabilistic model to estimate posterior inclusion probabilities while accounting for local LD structure derived from study genotypes and assuming a single causal variant. We set the other parameters to their default values and output variants from 95% credible sets by ranking them by posterior inclusion probability until the cumulative posterior probability reached 0.95. We report the distribution of credible set sizes across loci, providing a quantitative summary of fine-mapping resolution.

#### Partitioning heritability based on statistical fine-mapping

S-LDSC was used to test whether custom variant annotations from statistical fine-mapping of eQTL signals were enriched for heritability attributable to genetic risk for complex traits^3,64^. Partitioned heritability analyses were conducted using only autosomal variants, consistent with standard S-LDSC analyses. For each eGene, statistical fine-mapping was used to compute a posterior inclusion probability for each *cis*-variant, retaining variants in the 95% credible set for each gene. Each variant in the genome is annotated with a probability value from this analysis. Variants not in a 95% credible set receive a value of zero, and variants evaluated for multiple genes receive the maximum probability value for the variant across these genes. This approach is termed ‘MaxCPP’ in a previous publication^64^. S-LDSC was then used to partition trait heritability using the constructed functional annotations, using the 1000 Genomes Phase 3 European ancestry reference panel. The estimated enrichment was used to measure the importance of each eQTL category to human complex traits or diseases. To rule out potential influences of correlation among eQTL categories, we aggregated the baselineLD model, which includes a set of 75 functional annotations from a previous publication^65^, to create functional annotations for the eQTL category, then ran S-LDSC jointly and assessed significance using the enrichment *P* value.

#### Proportion of disease heritability mediated by regulatory variants

Mediated expression score regression (MESC) estimates the proportion of disease heritability mediated by regulatory variants for a specific set of molecular traits^27^. We applied this approach to estimate the contribution of regulatory variants across cell classes and subclasses to the heritability of complex traits. MESC was then used to calculate mediated heritability with default settings. To estimate the joint contribution of subtypes from one cell class, we also ran meta-analyzed MESC using meta_analyze_weights.py. Following the package’s instructions, analysis was performed on all expressed genes. No additional filtering was performed based on *cis*-eQTL or *trans*-eQTL results, fine-mapping or other criteria.

#### Colocalization of genetic signals from regulatory and disease risk variants

To evaluate the relationship between molecular QTLs, we used the coloc R package to conduct colocalization analysis^66^. The summary results from meta-analysis were used as input for coloc. Colocalization was performed within overlapped regions between eQTL and GWAS summary statistics centered around the gene body. Consistent with previous work by our group^8^ and others^67,68^, no *P* value thresholds were used, so it is possible to identify colocalization with a locus that does not reach genome-wide significance. The phenotypic variance was set to 1 because we had normalized the summary results before meta-analysis; otherwise, parameters were set to their default values. We also applied an extension, moloc^69^, that applies this framework to identify the colocalization of three signals. For coloc analyses, we considered signals between two traits to be colocalized at a posterior probability of ≥0.8 (that is, PP4 ≥ 0.8).

#### Identifying shared and cell type-specific genetic regulatory effects

To determine how eQTL effects are shared between different cell types, we applied a multivariate Bayesian meta-analysis approach using the mashr software (v0.2.79)^33^. This software uses a Bayesian approach to shrink effect sizes across genes and cell types to estimate the posterior effect sizes and posterior probability that an effect has the correct sign. Following guidelines in the mashr documentation, we estimated the prior effect size distribution using a collection of genes expressed in all cell types and learned the empirical correlation structure using 600,000 randomly selected variant–trait pairings. For all genes with a genome-wide significant eQTL in at least one cell type, the variant with the smallest *P* value was selected, and the coefficient estimate and standard error were used in analysis with mashr. For genes not analyzed in a particular cell type owing to insufficient expression, values of zero were used for the coefficient, and 1 × 10^6^ was used for the standard error.

We extend this approach to develop a formal statistical test to identify cell type-specific genetic regulatory effects. Although the mashr analysis integrates results across cell types, the software characterizes the regulatory effect of a variant in one cell type at a time. Mashr tests whether a genetic effect is present in a given cell type. By contrast, we directly test whether a genetic effect is cell type-specific using a composite test that assesses whether the effect is non-zero in a given cell type while also being zero in all other cell types.

Here, we describe the math of the composite test. For a gene *j* and cell type *i*, mashr reports the local false sign rate defined as ${\mathrm{lfsr}}_{j,i}=\min \left[p({\beta }_{i,j}\ge 0|\hat{\beta },\ldots ),p({\beta }_{i,\,j}\le 0|\hat{\beta },\ldots )\right]$, where $({\beta }_{i,\,j}\ge 0|\hat{\beta },\ldots )$ is the probability that the true value of the regression coefficient is greater than zero given the estimated coefficient and other model parameters^70^. Thus, ${\mathrm{lfsr}}_{i,\,j}$ is the posterior probability that the sign of the estimated coefficient does not agree with the sign of the true coefficient value, and it is more conservative than the local false discovery rate^70^. Then let ${p}_{i,\,j}=1-{\mathrm{lfsr}}_{i,\,j}$ be the probability that the signs agree. For a given gene, this set of posterior probabilities can be used to estimate the probability of any combination of eQTL effects across cell types. Therefore, ${p}_{1,j}$ is taken as a conservative estimate of the probability that a genetic variant has a non-zero effect size in cell type 1, and $1-{p}_{2,\,j}$ is taken as a conservative estimate that the effect is zero in cell type 2. Combining these estimates, the probability of a non-zero effect in cell type 1 and a zero effect in cell type 2 is ${p}_{1,j}\left(1-{p}_{2,j}\right)$, assuming the probabilities are independent. In general, the probability of an arrangement with non-zero effects in set 1 and all zero effects in set 2 is $\left[{\varPi }_{i\in \mathrm{set}1}{p}_{i,\,j}\right]\left[{\varPi }_{i\in \mathrm{set}2}\left(1-{p}_{i,\,j}\right)\right]$.

Owing to limited statistical power to detect eQTLs in high-resolution cell types, it is often too restrictive to ask, for example, whether an eQTL effect is non-zero in all excitatory neuron subtypes. Instead, we can ask whether there is a non-zero effect in at least one subtype by evaluating $1-\left[{\varPi }_{i\in \mathrm{set}1}\left(1-{p}_{i,\,j}\right)\right]$.

#### Aging-related dynamic eQTL detection

As part of the PsychAD Consortium, an analysis of the transcriptional dynamics of normal aging across the human lifespan was performed in a companion paper^34^. Using the PsychAD dataset, the authors extracted data from 284 neurotypical postmortem donors aged 0–97 years, comprising 1.3 million nuclei, and divided donors into six developmental groups: neonatal (0–1 year old, *n* = 9), childhood (2–11 years, *n* = 11), adolescence (12–19 years, *n* = 33) and young (20–39 years, *n* = 54), middle (40–59 years, *n* = 95) and late adulthood (≥60 years, *n* = 82). The authors^34^ then constructed a pseudotime trajectory within each cell type, using a supervised method incorporating donor age by applying the UMAP of MATuration (UMAT) method. This approach constrains the projection to a low-dimensional space by restricting UMAP neighbor selection to nuclei from adjacent developmental stages^35^. This constraint produces a pseudotime trajectory that is consistent with the known developmental ordering of the donors based on age. Nuclei from the full PsychAD dataset were then projected onto this UMAT space, and each nucleus was assigned a pseudotime score corresponding to its cell type.

Dynamic QTL analysis was then performed at the single-nucleus level for each cell type by testing whether the estimated genetic effect of a given variant on a gene expression trait changed along the pseudotime trajectory. For a given cell type, every nucleus was included in a regression model that tested an interaction effect between genetic variant and pseudotime. Raw count data for gene expression were analyzed using a negative binomial mixed model (NBMM), with donor as a random effect. We found this NBMM to be critical to controlling the false-positive rate in our dataset. Covariates for library size, age, sex and mitochondrial rate were included as fixed effects. Analyses were implemented using the glmer.nb() function in the lme4 R package^71^:


glmer.nb(y~pseudotime+SNP+pseudotime*SNP+covariates,…)


and *P* values were computed from a Wald test.

NBMM analysis at the single-nucleus level is very demanding, requiring approximately 1 h of processing time per regression because of the large number of nuclei included in each analysis (astrocytes, 763,000; excitatory neurons, 1.45 million; immune cells, 331,000; inhibitory neurons, 973,000; oligodendrocytes, 2.28 million; and OPCs, 363,000). Yet we found this NBMM to be critical to controlling the false-positive rate in our dataset. Owing to the high computational time, we followed the approach of previous work^13,23^ and analyzed one variant per gene for each cell type, selecting the top variant from the standard pseudobulk *cis*-eQTL analysis for each cell type.

The enrichment of genes with dynamic regulatory signals for colocalization with disease traits was evaluated as follows. For a cell class *i* with ${d}_{i}$ dynamic eGenes, the number of genes that also have a colocalization signal was computed. The null distribution of this count was evaluated by randomly sampling ${d}_{i}$ genes and evaluating the overlap with genes with a colocalization signal. The standard error of the overlap for each cell type was evaluated using 100 rounds of random sampling.

#### *Trans*-eQTL detection

Performing *trans*-eQTL analysis on a large number of single-nucleotide polymorphisms (SNPs) is computationally expensive and incurs a substantial multiple-testing burden. Following previous work^13^, we addressed both of these issues by selecting lead variants, including 56,204 eSNPs from the *cis*-eQTL analysis and 45,088 genome-wide significant variants from eight brain disease GWAS studies (that is, variants with *P* < 5 × 10^−8^ in AD, binge eating disorder, bipolar disorder, MDD, Parkinson’s disease, SCZ, attention deficit–hyperactivity disorder and autism spectrum disorder). Analysis was performed across all expressed genes in each cell class and restricted to autosomes, and excluded the X chromosome because of the increased statistical and biological complexity associated with modeling long-range regulatory effects involving sex chromosomes, yielding 8.74 billion tests. SNP genotype was included as the dependent variable in all gene–variant pairings in the linear regression model we tested. We defined *trans*-variants as variants located more than 5 Mb from target genes, and focused on autosomal chromosomes, excluding any signal within major histocompatibility complex regions. Genes with a mappability score of <0.8 were excluded to avoid false-positive *trans*-eQTL findings caused by reads mapping to multiple locations in the genome^10,72^.

We applied multiple-testing corrections at two levels following the approach of widely used methods for *cis*-eQTL analysis^62,73^. For a given gene, the lead eQTL variant is defined as the variant with the smallest *P* value from all variants tested for that gene. Other software programs use permutation analysis to compute gene-level *P* values that correct for this multiple testing^62,73^. Instead of performing computationally expensive permutations, we applied a Šidák correction to report a gene-level *P* value corrected for testing *k* variants, using $k=1\times {10}^{5}$. Letting ${P}_{\min }$ be the smallest *P* value observed for a given gene, the Šidák-corrected *P* value for the gene is ${P}_{S{idak}}=1-{\left(1-{P}_{\min }\right)}^{k}$. Given that we performed *trans*-eQTL analysis for six cell classes, gene-level *P* values were computed for each cell class. A second round of multiple-testing correction was applied across all genes and cell types by using the Benjamini–Hochberg method^74^ on these gene-level *P* values. Study-wide significant *trans*-eQTLs were identified at an FDR of 5%.

#### Mediation analysis of *cis*-mediated *trans*-eQTLs

To identify *trans*-eQTLs with evidence of mediation, we limited our exploration to *trans*-eSNPs with high LD (*r*^2^ ≥ 0.75) with at least one peak eQTL variant within 1 Mb. We applied the strategy developed in a previous publication^75^ to calculate the indirect impact of *trans*-SNPs on *trans*-eGenes. Multiple-testing correction using the Benjamini–Hochberg approach was applied to control the study-wide FDR at 5%.

#### Analysis of the X chromosome

We performed analysis on the X chromosome at the class and subclass level following the approach used by GTEx^76^. For variants located in the non-pseudoautosomal regions of the X chromosome, we accounted for sex-specific genotype dosage. Given that males are hemizygous for the X chromosome, their genotypes were coded as 0 (hemizygous reference) or 2 (hemizygous alternative), effectively doubling the allele dosage to match the diploid scale used for females. Genotypes in females were treated the same as autosomal variants (0, 1 or 2). For variants located in the pseudoautosomal regions of the X chromosome, genotypes were treated as autosomal because these regions are present on both the X and Y chromosomes and behave as diploid in both sexes. We then applied the same analysis pipeline as for autosomal chromosomes to run eQTL detection. Analysis was performed on each brain bank separately, and the results were combined using a fixed-effect meta-analysis. Effect size estimates showed high concordance across cell types, with sign concordance rates ranging from 84.8% to 97.8% (Supplementary Fig. 26). This analysis revealed between eight and 301 eGenes at the class level and between one and 213 eGenes at the subclass level (Supplementary Fig. 27).

### Reporting summary

Further information on research design is available in the Nature Portfolio Reporting Summary linked to this article.

## Online content

Any methods, additional references, Nature Portfolio reporting summaries, source data, extended data, supplementary information, acknowledgements, peer review information; details of author contributions and competing interests; and statements of data and code availability are available at https://doi.org/10.1038/s41588-026-02733-5.

## Supplementary information

Supplementary Information Supplementary Figs. 1–27.

Reporting Summary

Peer Review File

Supplementary Table 1 Size of credible sets from statistical fine-mapping.

Supplementary Table 2 Loci with eQTL–GWAS colocalization for multiple genes.

Supplementary Table 3 Intersection of *trans*-eGenes and *cis*-eGenes with disease colocalization.

Supplementary Table 4 Sample size for each cell class and subclass for eQTL analysis after filtering.

## Data Availability

Results from this analysis are openly available from https://www.synapse.org/Synapse:syn61929918/wiki/629719. Raw and processed data are described in a previous publication^25^ and are available at https://www.synapse.org/Synapse:syn60084804/wiki/628473, with approval required.

## Code availability

All source code used in this study is available at https://github.com/DiseaseNeuroGenomics/nps_ad and https://doi.org/10.5281/zenodo.21226934 (ref. ^77^).
