# Supplementary material for: Single-nucleus atlas of cell-type specific genetic regulation in the human brain
Source: Nat Genet. 2026 Sep 23;58(10):2525–34. doi: 10.1038/s41588-026-02733-5 (PMC13649572; doi:10.1038/s41588-026-02733-5)
Supplement: Supplementary file 1 [file 41588_2026_2733_MOESM1_ESM.pdf]

nature genetics

Supplementary information https://doi.org/10.1038/s41588-026-02733-5

# Single-nucleus atlas of cell-type specific genetic regulation in the human brain

In the format provided by the authors and unedited

# Contents

**1 Supplementary Figures** **3**

# 1 Supplementary Figures

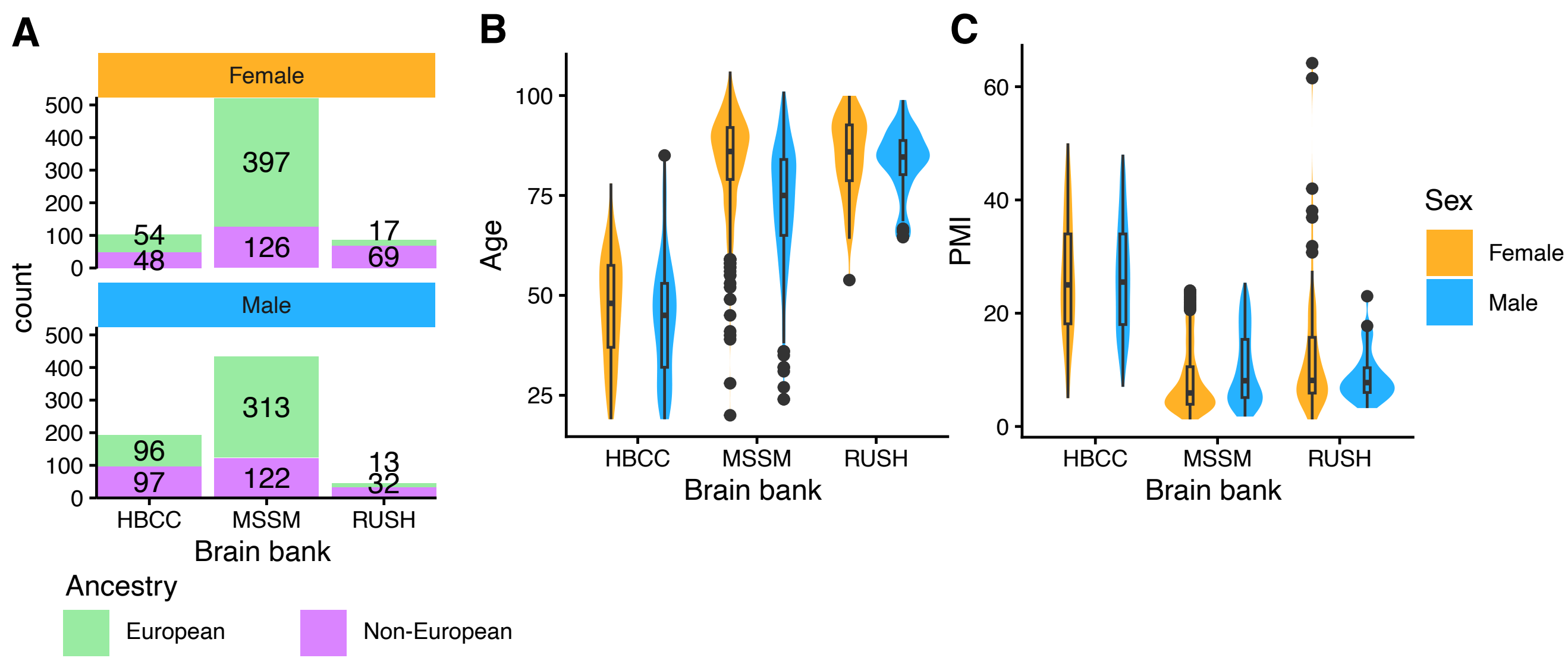


**Figure S1: Demographics of subjects included in analysis. A**) Count of male and female subjects in each cohort and ancestry group. **B**) Age distribution of subjects stratified by sex and cohort. **C**) Postmortem interval (PMI, in hours) distribution of subjects stratified by sex and cohort.

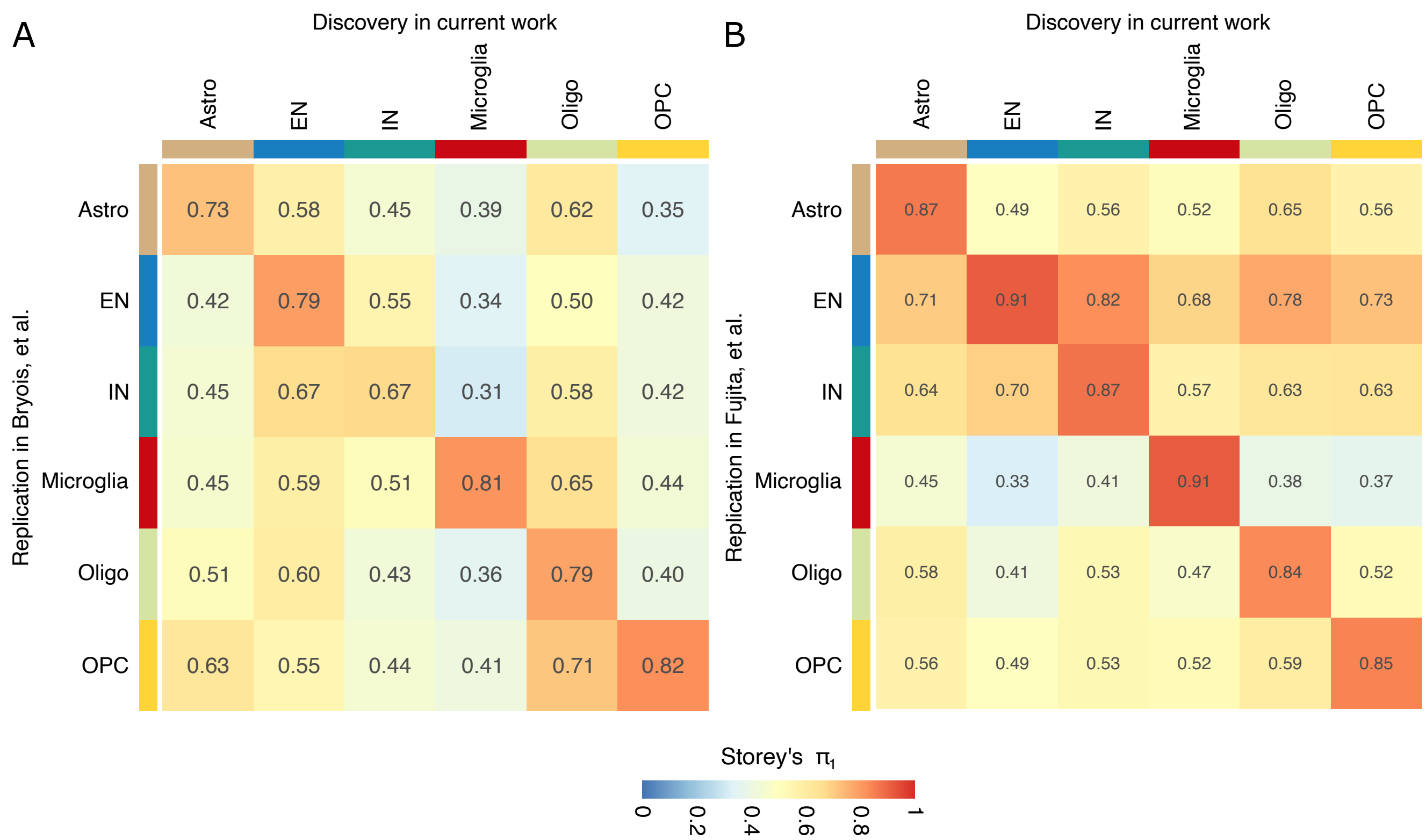


**Figure S2: Replication rate of regulatory variants from each cell class estimated by Storey's $\pi_1$.** The current dataset (columns) is used for eQTL discovery and the replication rate is evaluated along to rows for **A**) Bryois, et al. (2022) and **B**) Fujita, et al. (2024). For example, lead eQTL variants discovered in astrocytes in the current work were replicated in astrocytes at a rate of 73%, but only 42% in EN in Bryois, et al. The fact that the strongest replication rate is seen along the diagonal for the matching cell type indicates replication of the specific regulatory architecture of each cell type.

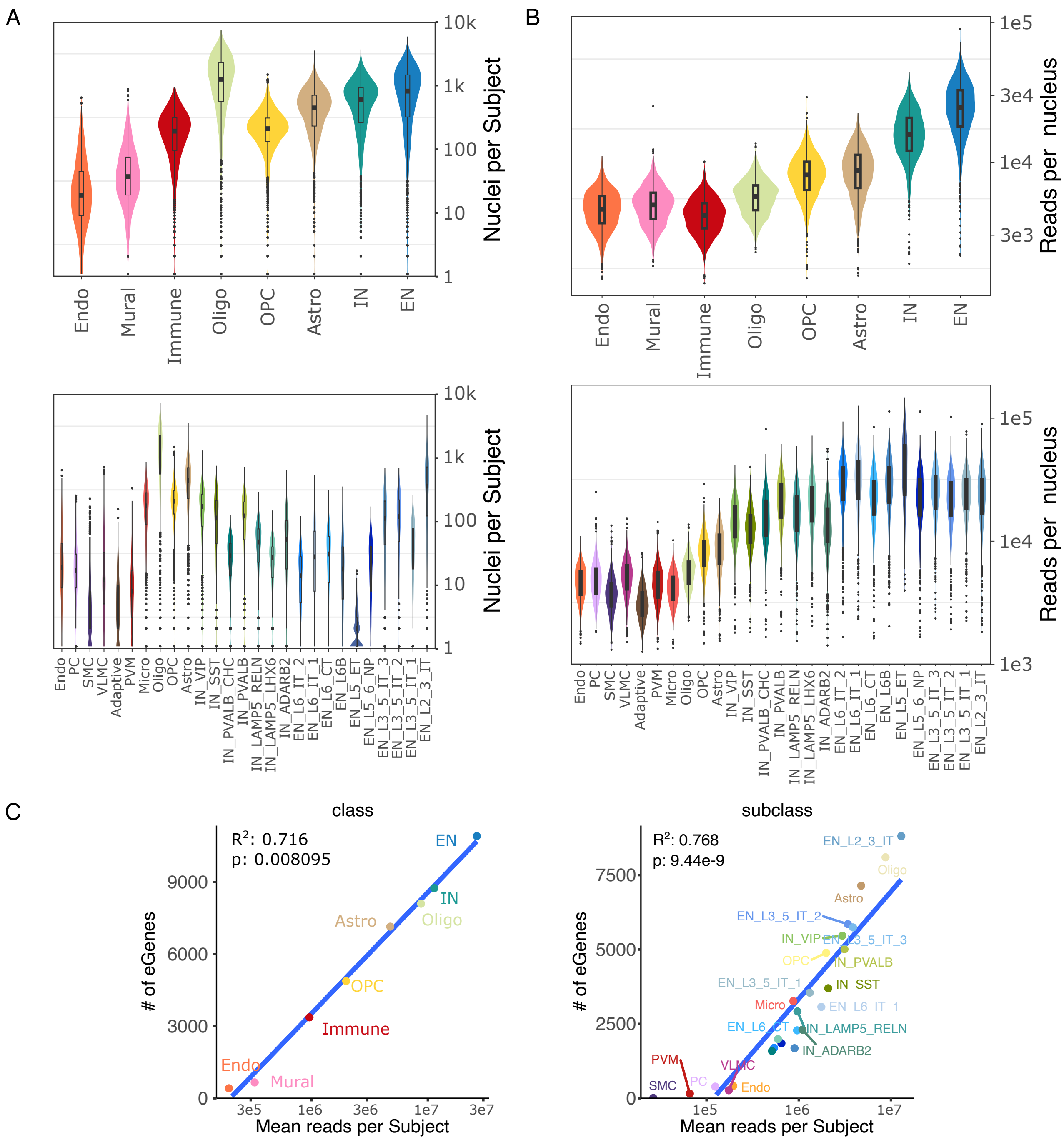


**Figure S3: Relationship between number of cells, reads and eQTL detection A)** Number of nuclei per subject for class (top) and subclass (bottom). **B)** Number of reads per nucleus for class (top) and subclass (bottom). **C)** The number of genes with significant eQTLs (i.e. eGenes) detected in each class (left) or subclass (right) shown as a function of the mean reads per subject for each cell type. Blue line indicates least squares fit. Squared Pearson correlation and p-value from linear regression are shown.

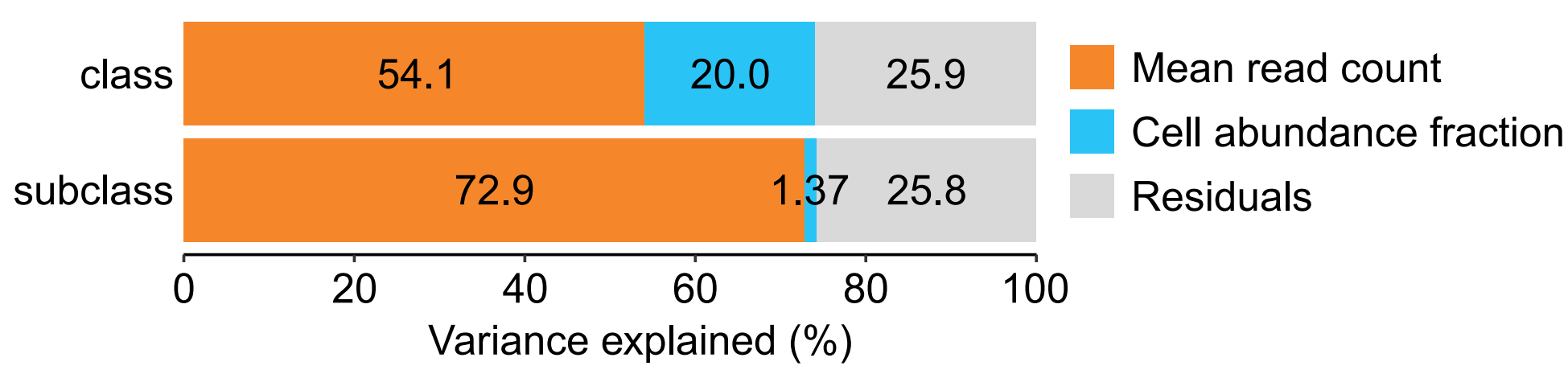


**Figure S4: Number of detected eGenes per cell type explained by mean read count.** A multiple regression was fitted, modelling the number of detected eGenes as the response variable and mean read count and abundance fraction as predictors. Variance partitioning analysis then quantified the fraction of variance explained by each variable. Analysis was performed for cell class and subclass. At each cell type level, the mean read count explained substantially more variance in the number of eGenes discovered than the cell abundance fraction. This is consistent with our intuition that statistical power to identify genetic regulatory variants for a given gene increases with the number of RNA-seq reads observed. Increasing the cell-abundance fraction increases power, but it does so by increasing the number of RNA-seq reads observed.

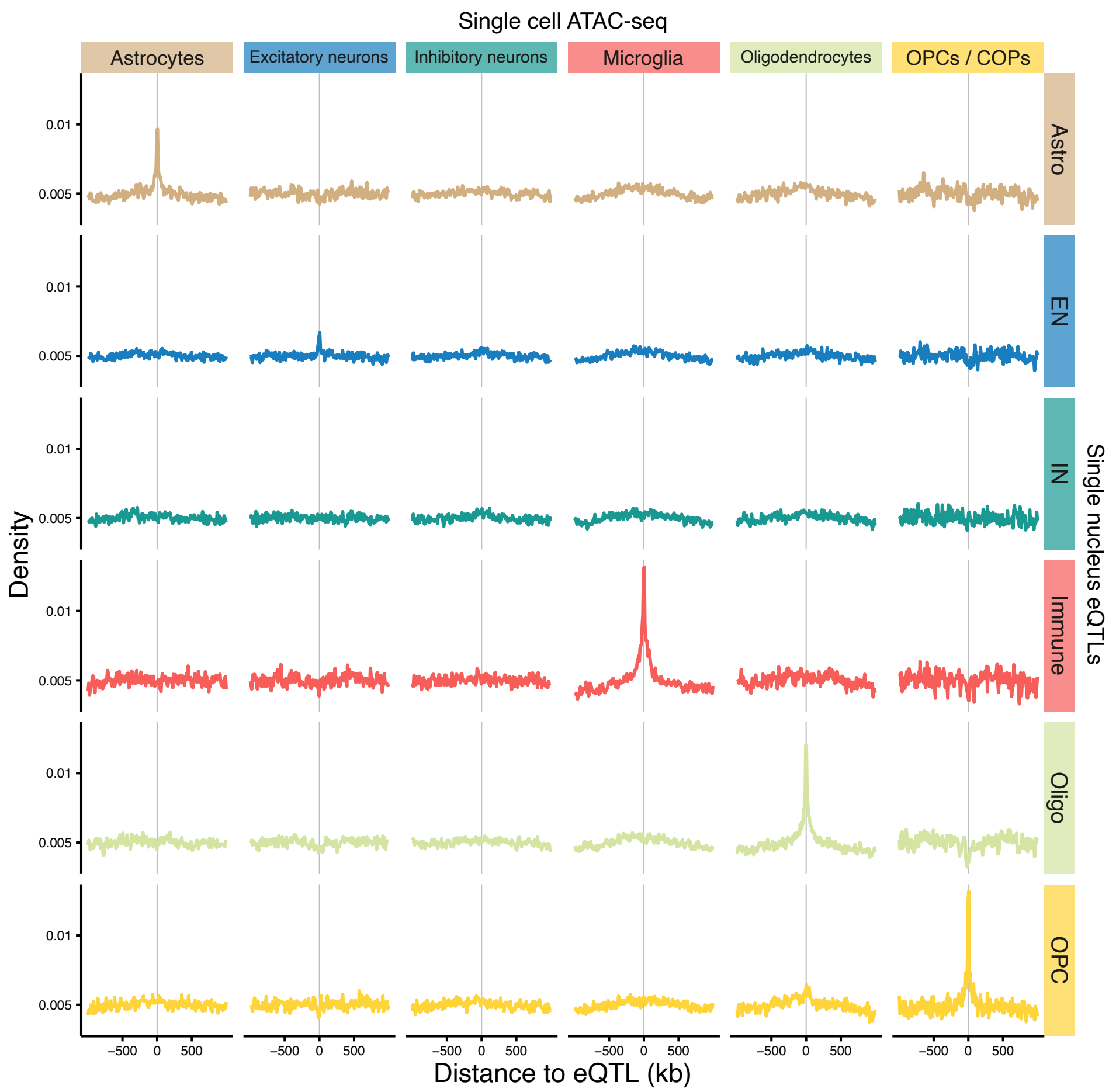


**Figure S5: Enrichment of open chromatin regions near lead eQTL variants.** Enrichment is shown for eQTLs detected in each cell class (rows) for open chromatin regions detected in each cell type from single-cell ATAC-seq (columns). Cell type annotations from Corces et al. (2020) are used for ATAC-seq data. 'COP' indicates committed oligodendrocyte precursor cells.

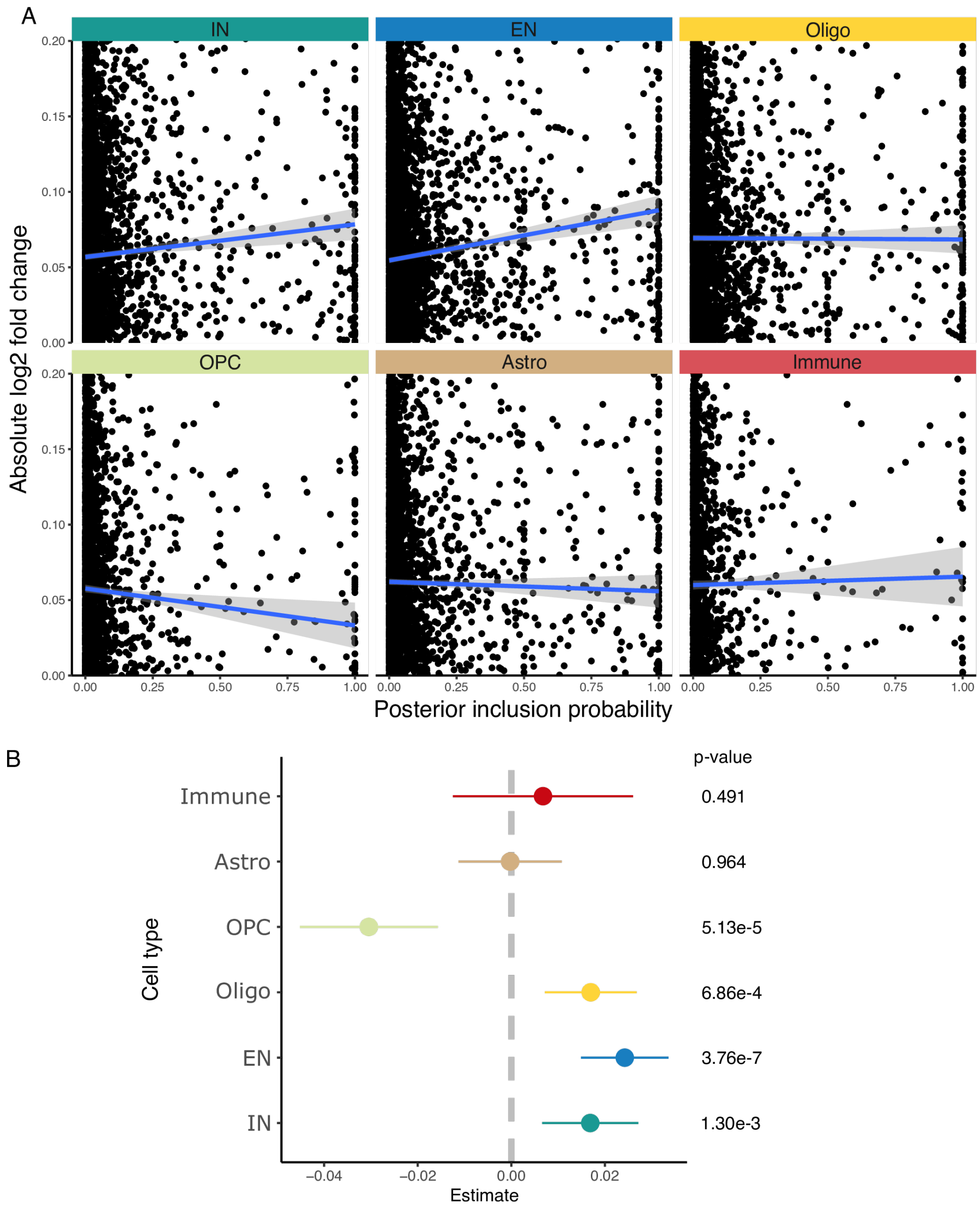


**Figure S6: Integration with allelic effects from multiplexed parallel reporter assay (MPRA). A)** The magnitude of allelic effects from MPRA increases with the fine-mapping posterior inclusion probability (PIP) from eQTL analysis. The best-fit line is shown in blue, and the gray band shows standard error. Although some SNPs are excluded from this plot because of the y-axis range, all SNPs were used to fit the regression model. **B)** Slope of lines from (A) showing the association between MPRA allelic effect size and PIP. Error bars indicate 95% confidence interval. P-value from hypothesis test comparing each estimate to zero is shown on the right.

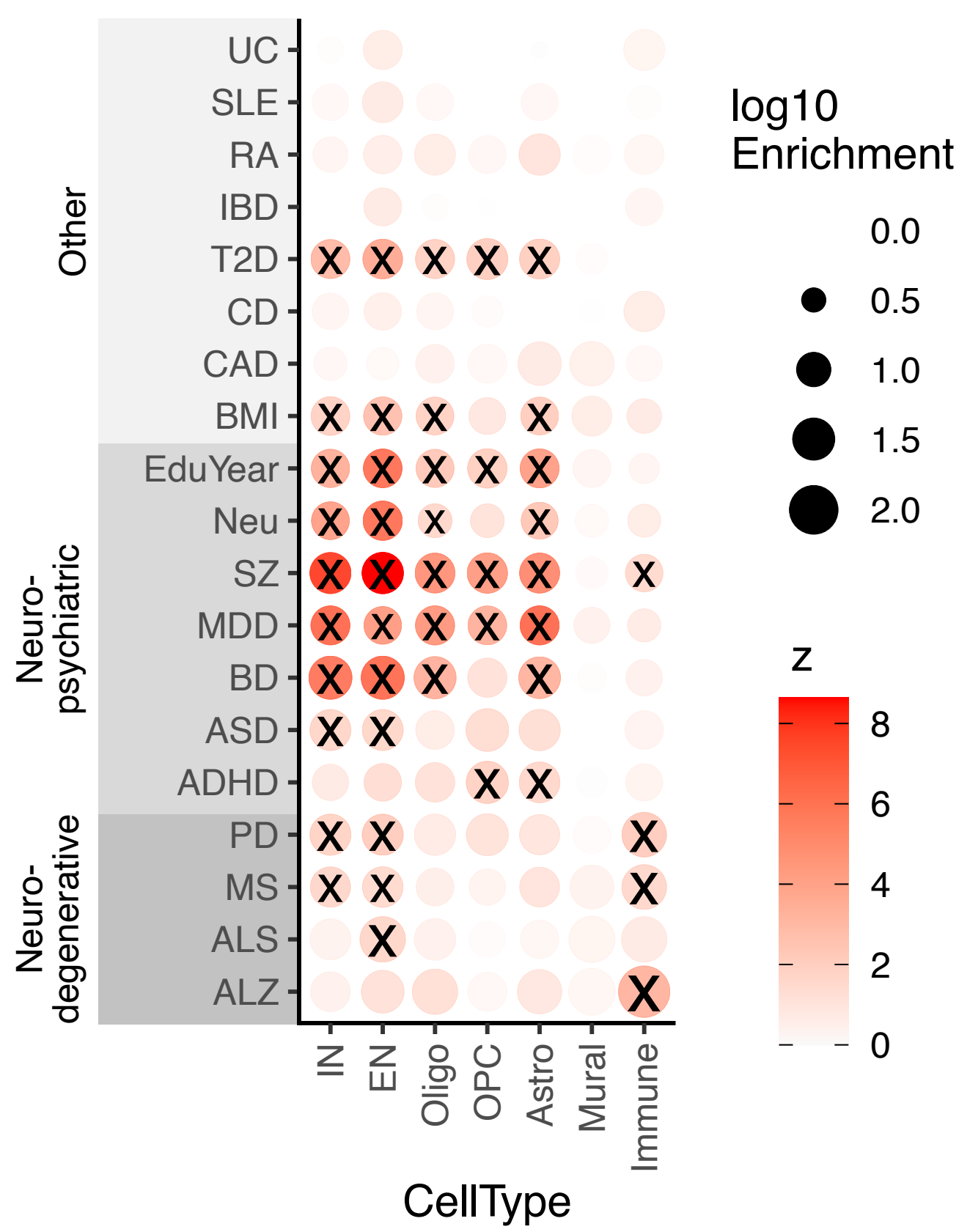


**Figure S 7: Regulatory variants are enriched for heritability of complex traits.** Enrichment of genetic regulatory variants in the 95% credible set from statistical fine-mapping for heritability of genetic traits. Enrichment of genetic regulatory variants in the 95% credible set from statistical fine-mapping for heritability of genetic traits. For example, for inhibitory neurons (IN), a new annotation was constructed with 1's for variants that were in the 95% credible interval for any eQTL detected in IN and 0's for all other variants. The enrichment of trait heritability explained by variants in this new annotation was then estimated by stratified LD-score regression. Color indicates the z-statistic of the null hypothesis of no enrichment, and point size indicates log10 enrichment. Tests with FDR < 5% are indicated by 'X'.

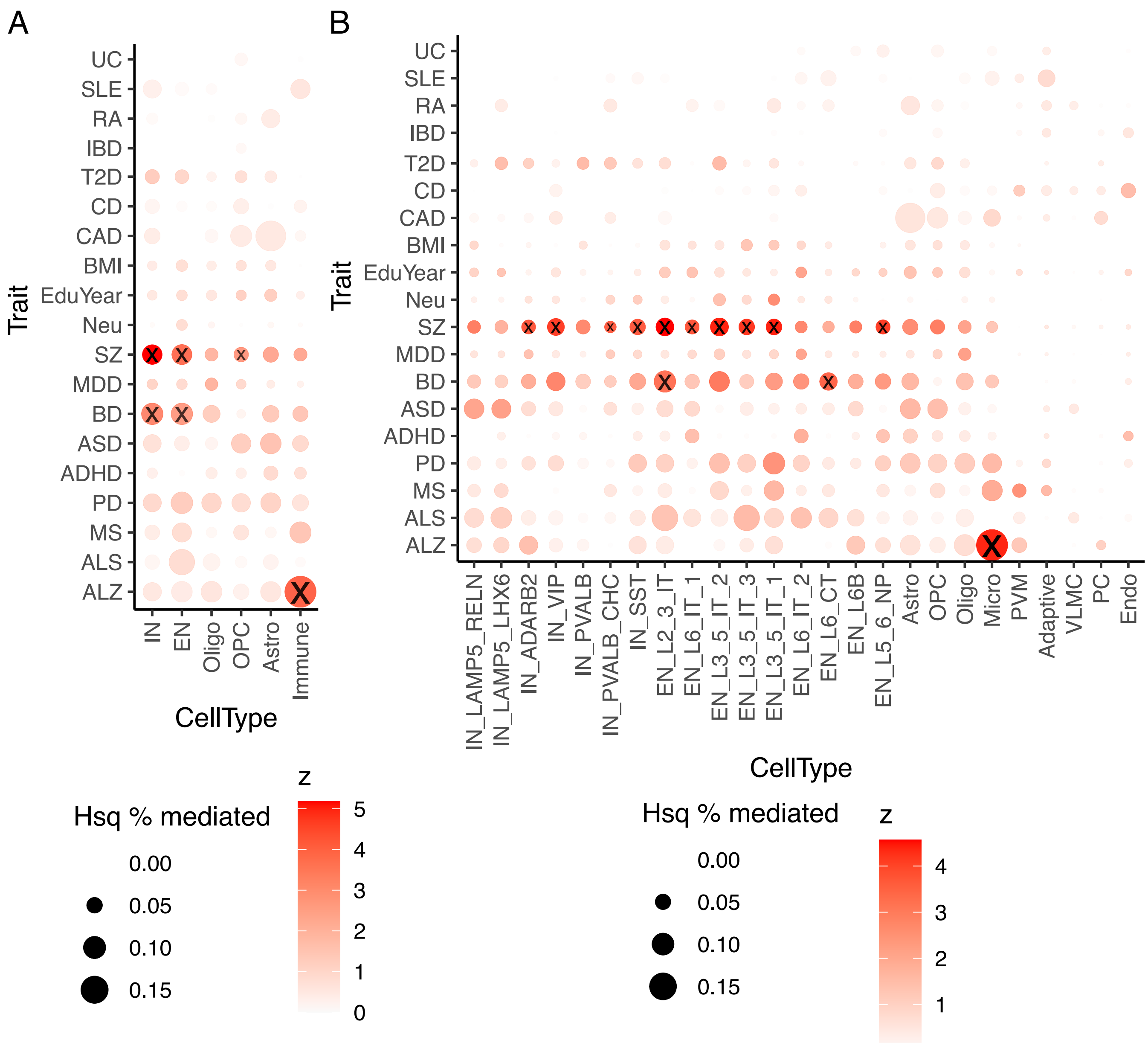


**Figure S 8: Mediation of trait heritability**. Fraction of heritability of complex traits mediated by regulatory variants at the **A)** class and **B)** subclass levels. Tests with FDR < 5% are indicated by 'X'. Analysis was performed on all expressed genes, with no additional filtering based on eQTL or fine-mapping.

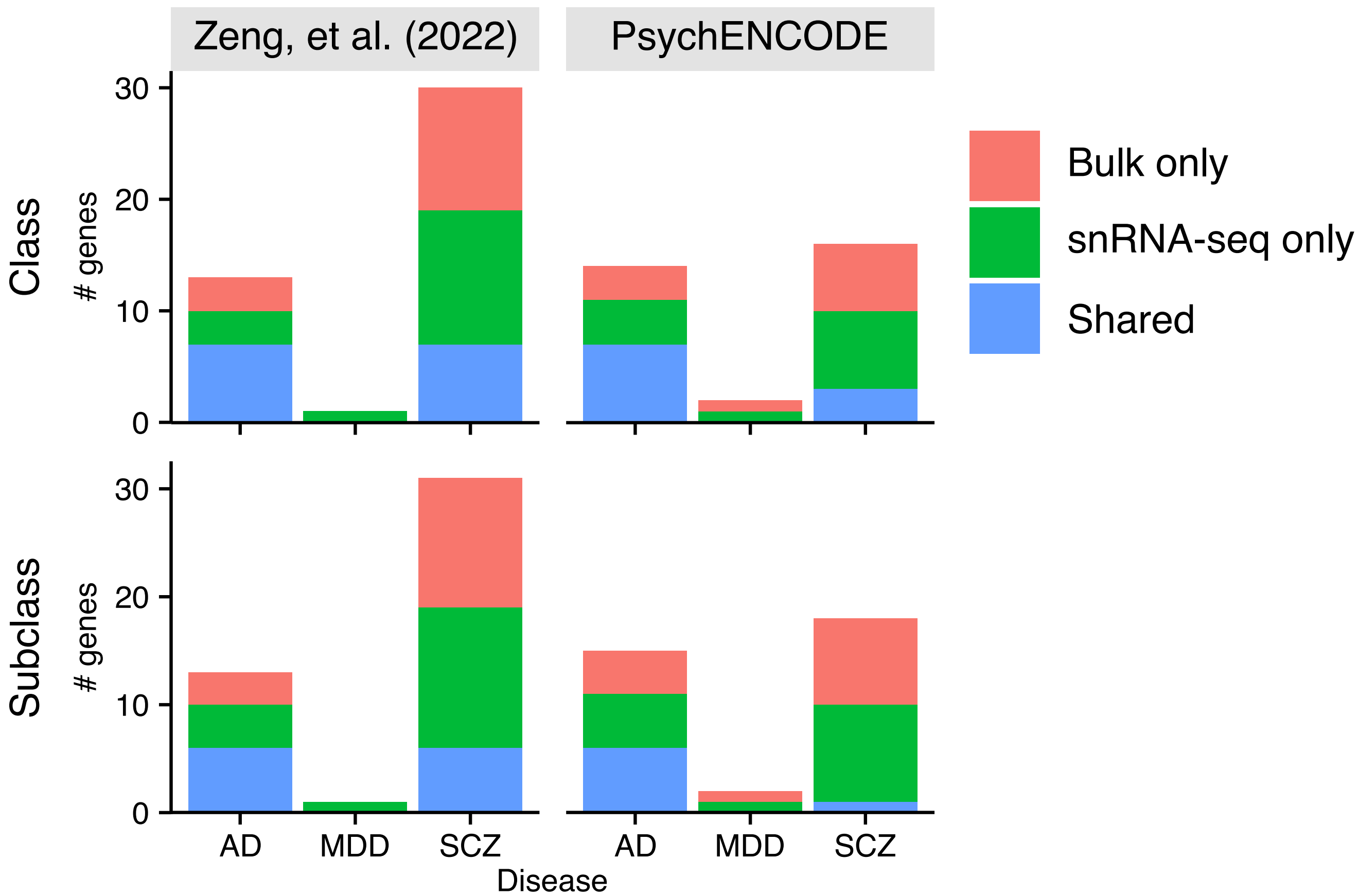


**Figure S9: Colocalization of genetic regulatory and disease risk signals in single-cell and bulk analyses.** Colocalization signals with a posterior probability greater than 0.8 are shown for single-cell data at both the class (top) and subclass (bottom) levels. Bulk tissue QTLs were derived from RNA-seq data in Zeng, et al. (2022) (left) and PsychENCODE (2018) (right). Colors indicate signals detected exclusively in bulk analyses (red), exclusively in single-nucleus RNA-seq (snRNA-seq) analyses (green), or shared between both approaches (blue). In comparison with Zeng, et al. and PsychENCODE, 16 and 12 genes, respectively, were uniquely identified using snRNA-seq at the gene level. At the subclass level, these counts increased to 18 and 15, respectively. Results are shown for Alzheimer's disease (AD), major depressive disorder (MDD), and schizophrenia (SCZ).

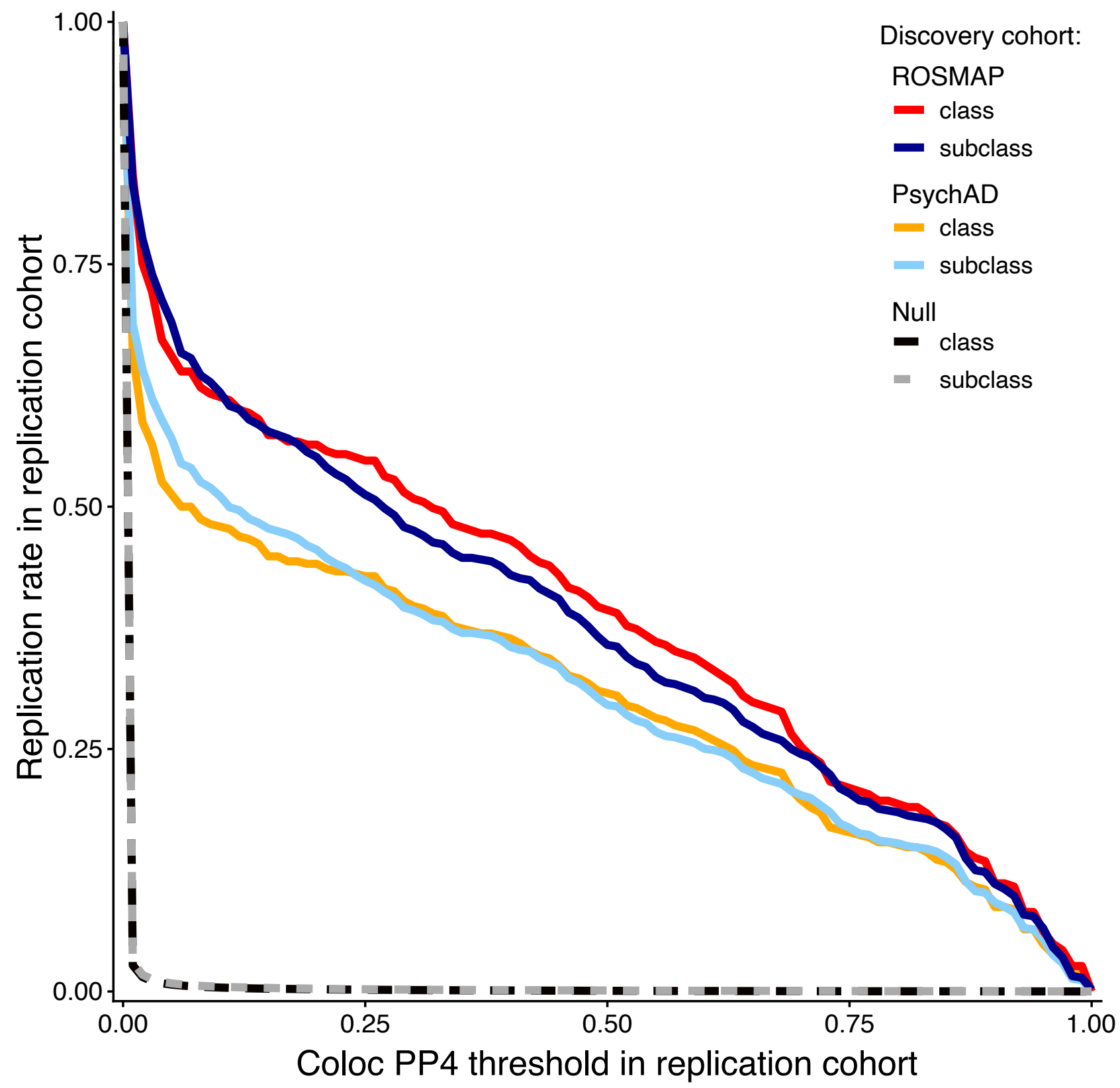


**Figure S 10: Replicating GWAS-eQTL colocalizations.** For the discovery cohort, all GWAS-eQTL colocalization with PP4 $> 0.8$ discovered at the class and subclass levels were examined in the matching cell type in the replication cohort. The replication rate is calculated over a range of PP4 cutoffs from 0 to 1, so that the replication rate decreases as the cutoff becomes stricter. Each of PsychAD and ROSMAP were considered as the replication cohort, and the replication rate was evaluated in the other cohort across a range of PP4 values. Since the ROSMAP cohort has fewer samples, the colocalizations found here had a higher replication rate across all cutoffs than those in the larger PsychAD cohort. By randomly selecting a gene in ROSMAP and comparing its colocalization level with the PP4 threshold, the replication rate under the null hypothesis of no colocalization was assessed.

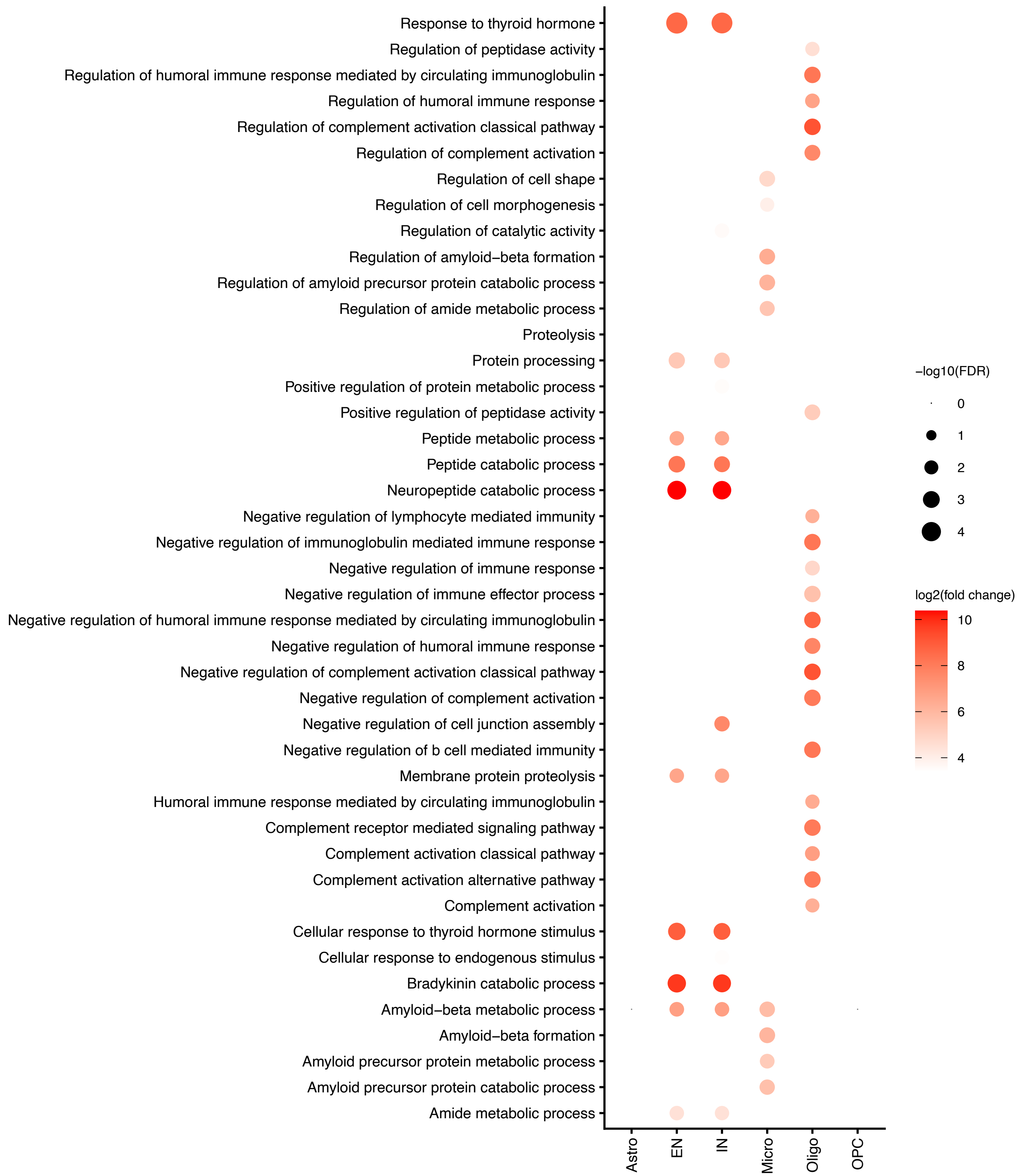


**Figure S11: Gene set enrichment analysis for each cell class**. Enrichment analysis was performed on the genes colocalizing with AD GWAS in each cell class.

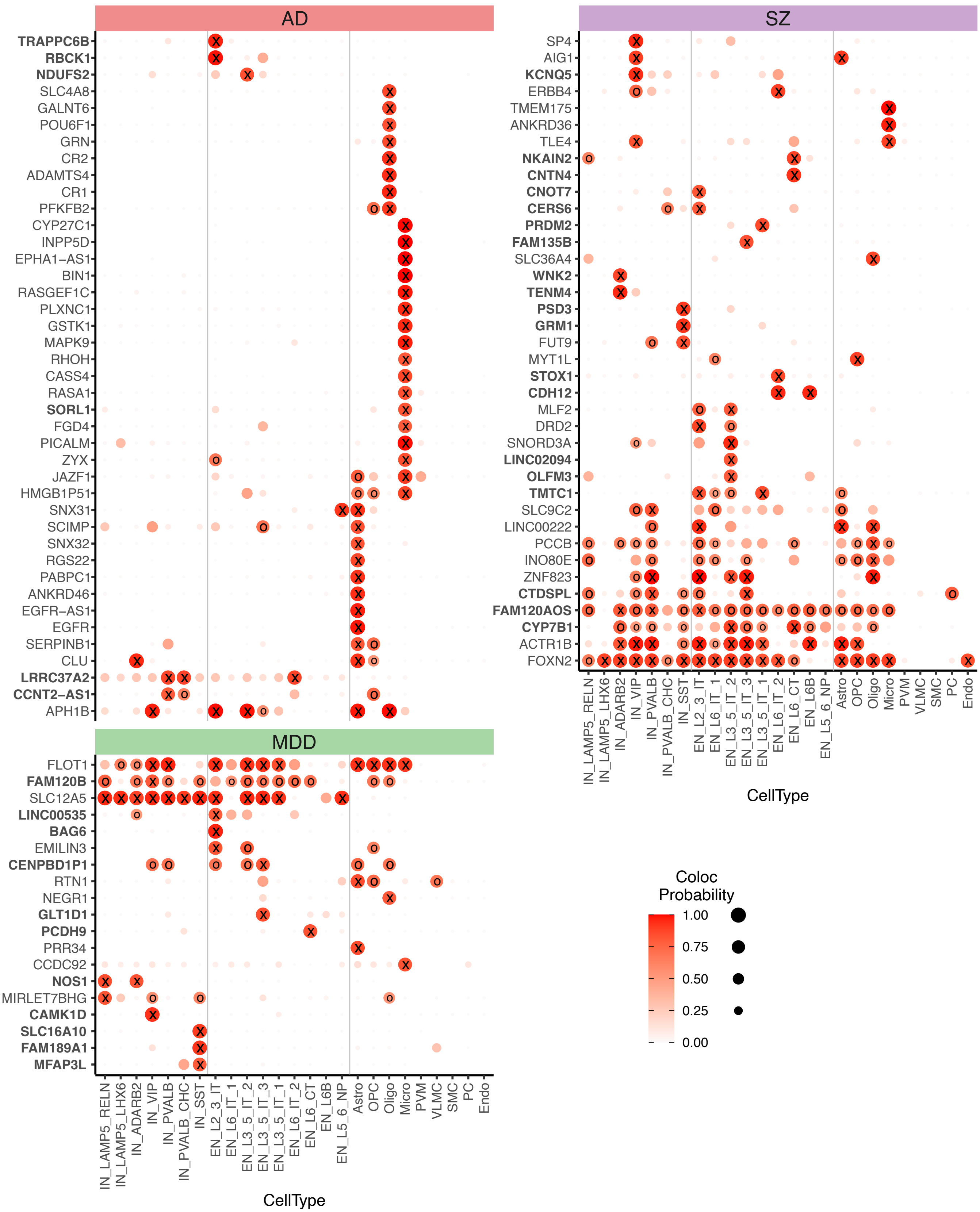


**Figure S12: Colocalization signal at the subclass-level**. Posterior probabilities > 0.8 are indicated by 'X', and > 0.5 are indicated by 'o'. Genes in bold do not have colocalization posterior probability > 0.8 at the class level.

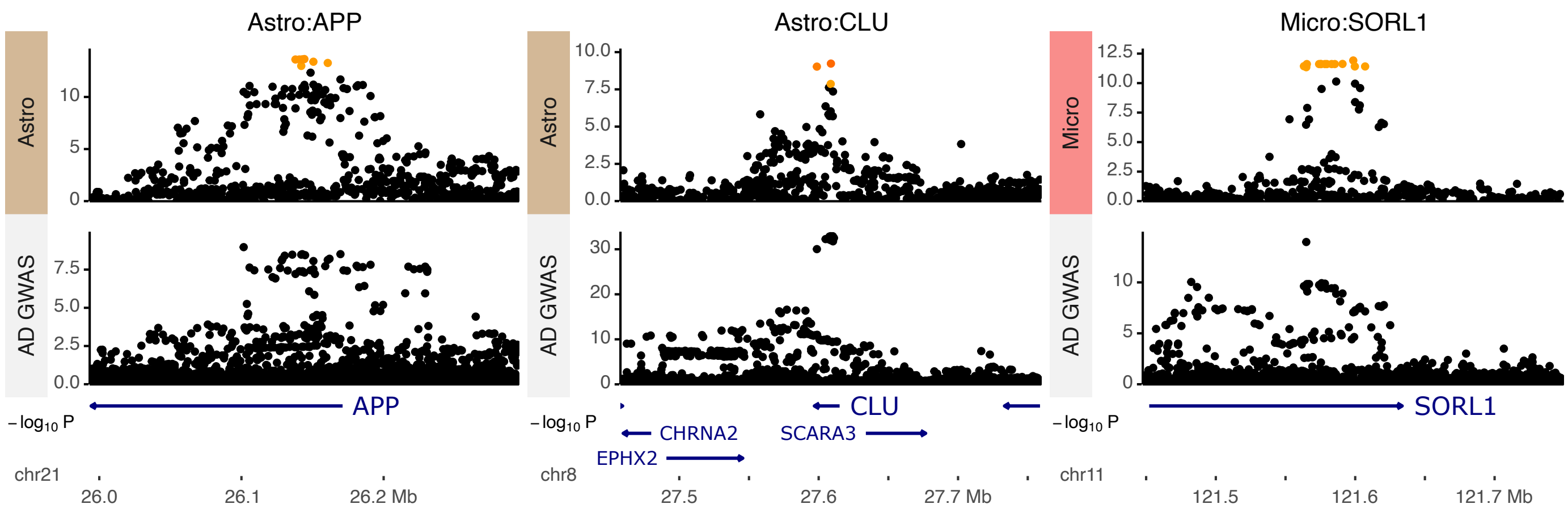


**Figure S13: Manhattan plots for colocalization signals.** Colocalization with AD genetic risk is observed for APP in astrocytes (left), CLU in astrocytes (center), and SORL1 in microglia (right).

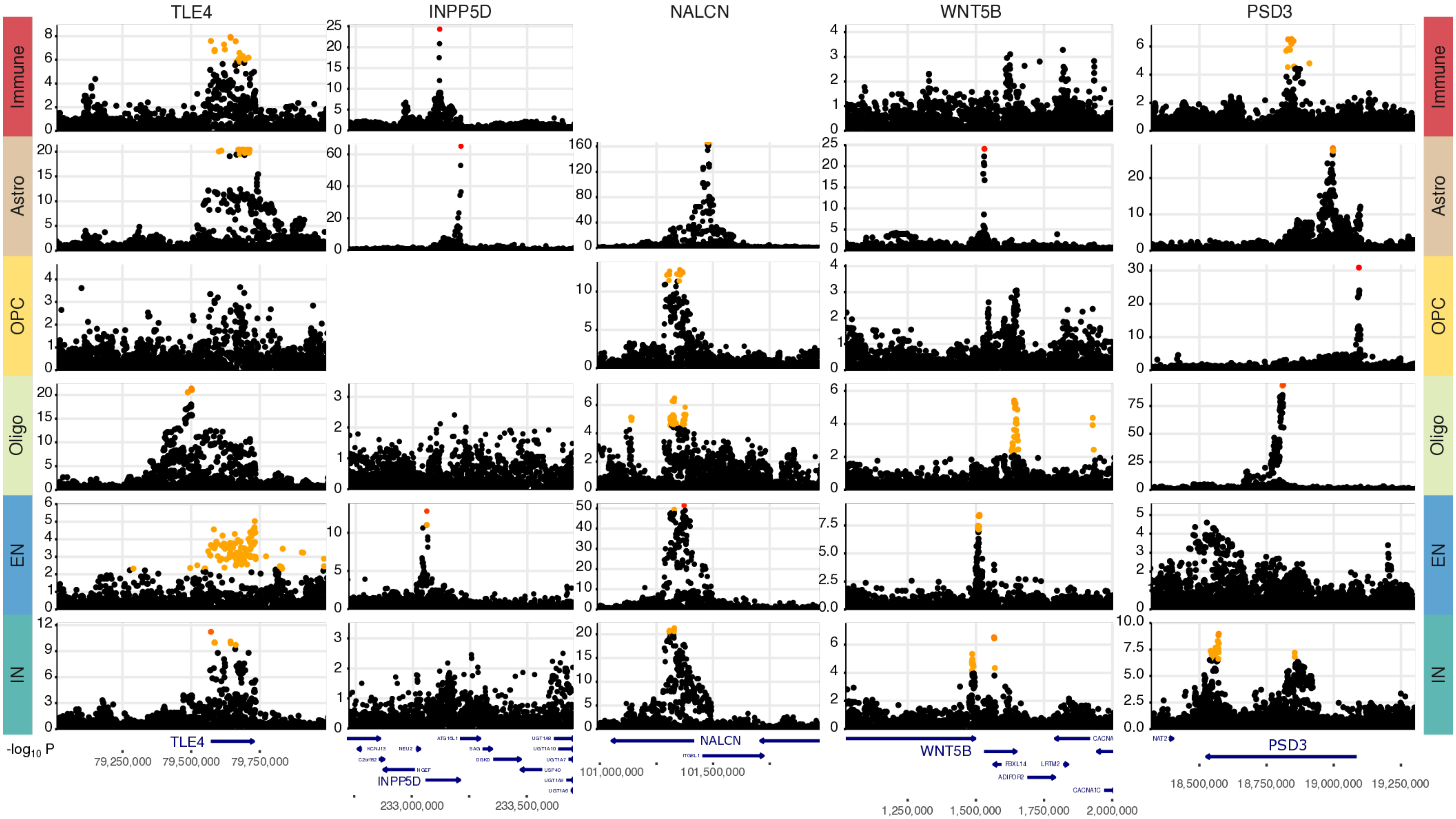


**Figure S14: Manhattan plot of genes with independent eQTL signals in different cell types.** Color indicates posterior inclusion probability from statistical fine-mapping. Blank panels indicates case where the gene is not sufficiently expressed in the given cell type.

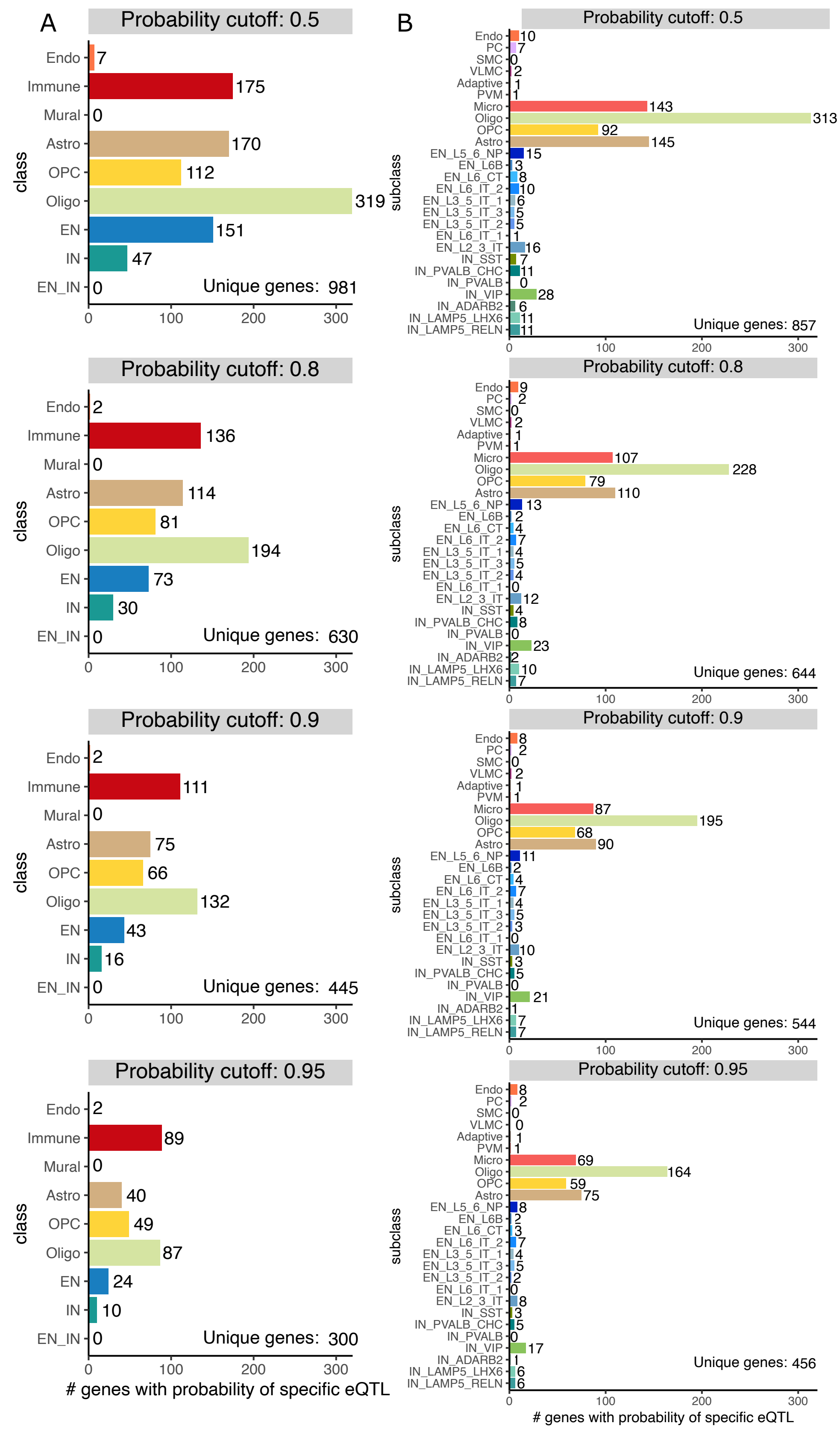


**Figure S15: Cell-type-specific findings using multivariate Bayesian meta-analysis followed by composite test.** Number of genes with cell type specific effects detected in each **A**) class or **B**) subclass across multiple posterior probability thresholds. The number of unique genes is reported for each threshold.

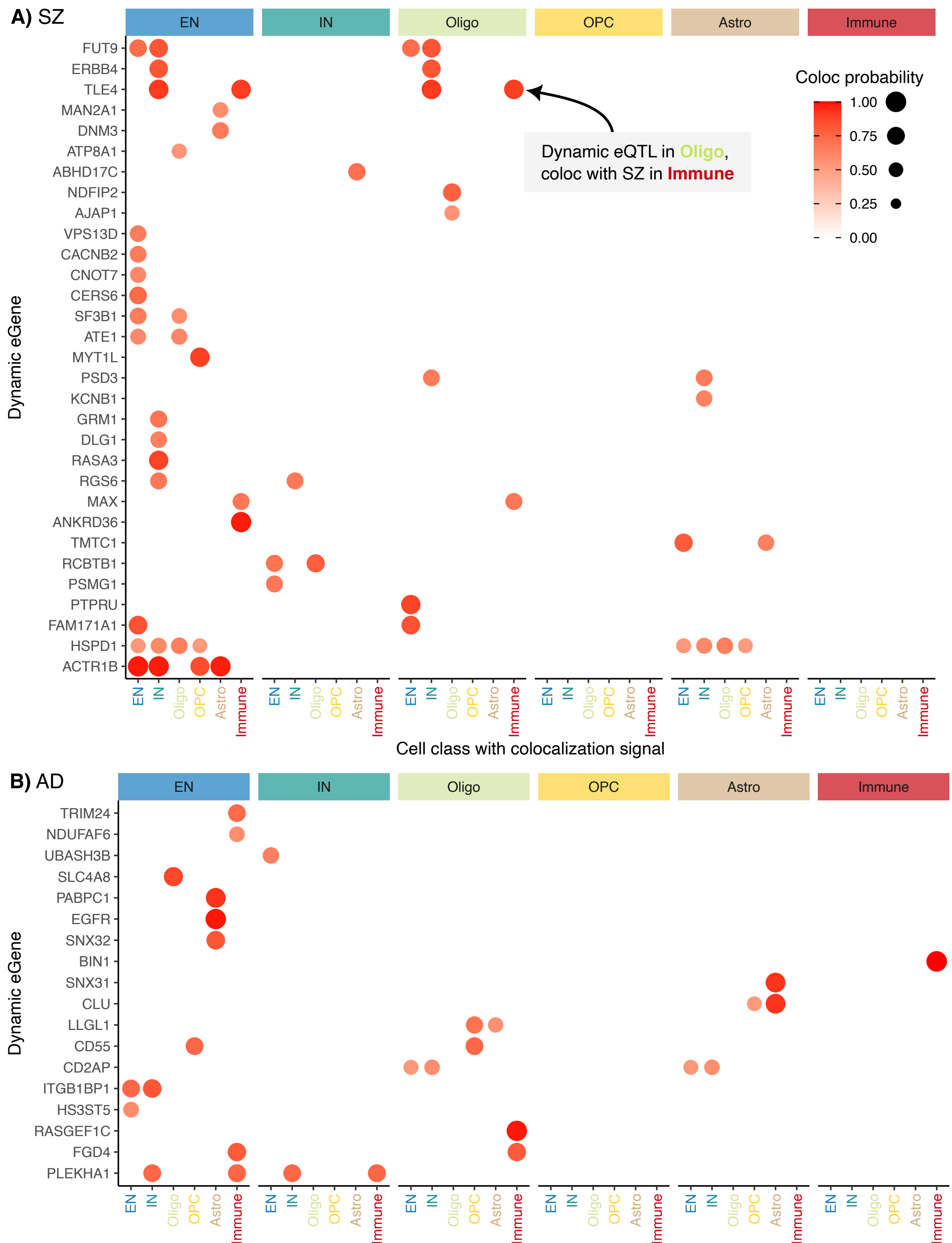


**Figure S16: Genes with a dynamic eQTL and a disease colocalization signal.** Genes with a dynamic eQTL (shown in inset of each cell class) that have a disease colocalization signal (shown on x-axis). For example, the gene TLE4 has a dynamic eQTL detected in oligodendrocytes and regulatory variants for this gene colocalize with AD risk in immune cells. Color and size of circle indicates posterior probability from colocalization analysis. Results are shown for **A**) SZ and **B**) AD.

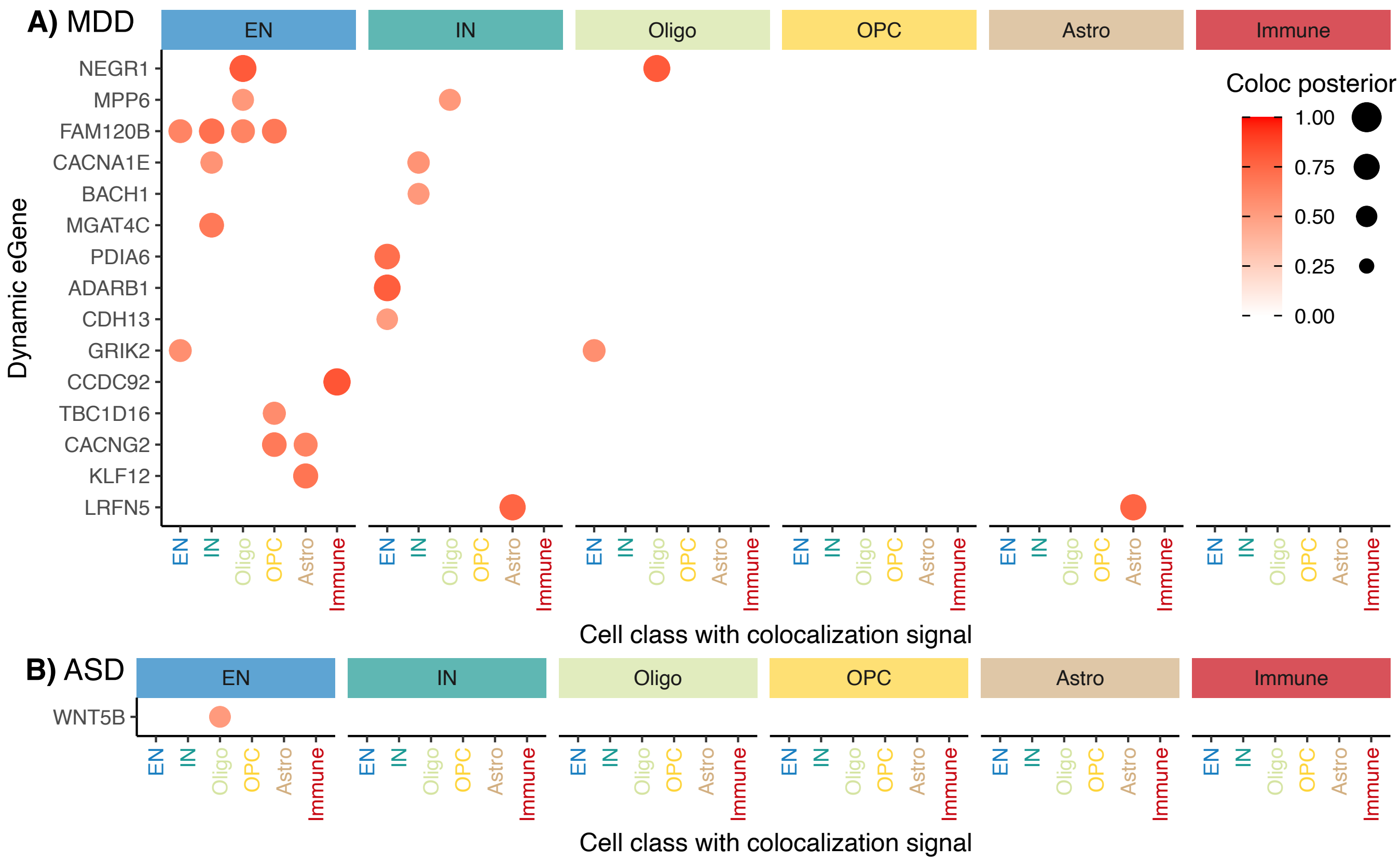


**Figure S17: Genes with a dynamic eQTL and a disease colocalization signal.** Genes with a dynamic eQTL (shown in inset of each cell class) that have a disease colocalization signal (shown on x-axis). For example, the gene TLE4 has a dynamic eQTL detected in oligodendrocytes and regulatory variants for this gene colocalize with AD risk in immune cells. Color and size of circle indicates posterior probability from colocalization analysis. Results are shown for **A**) MDD and **B**) ASD.

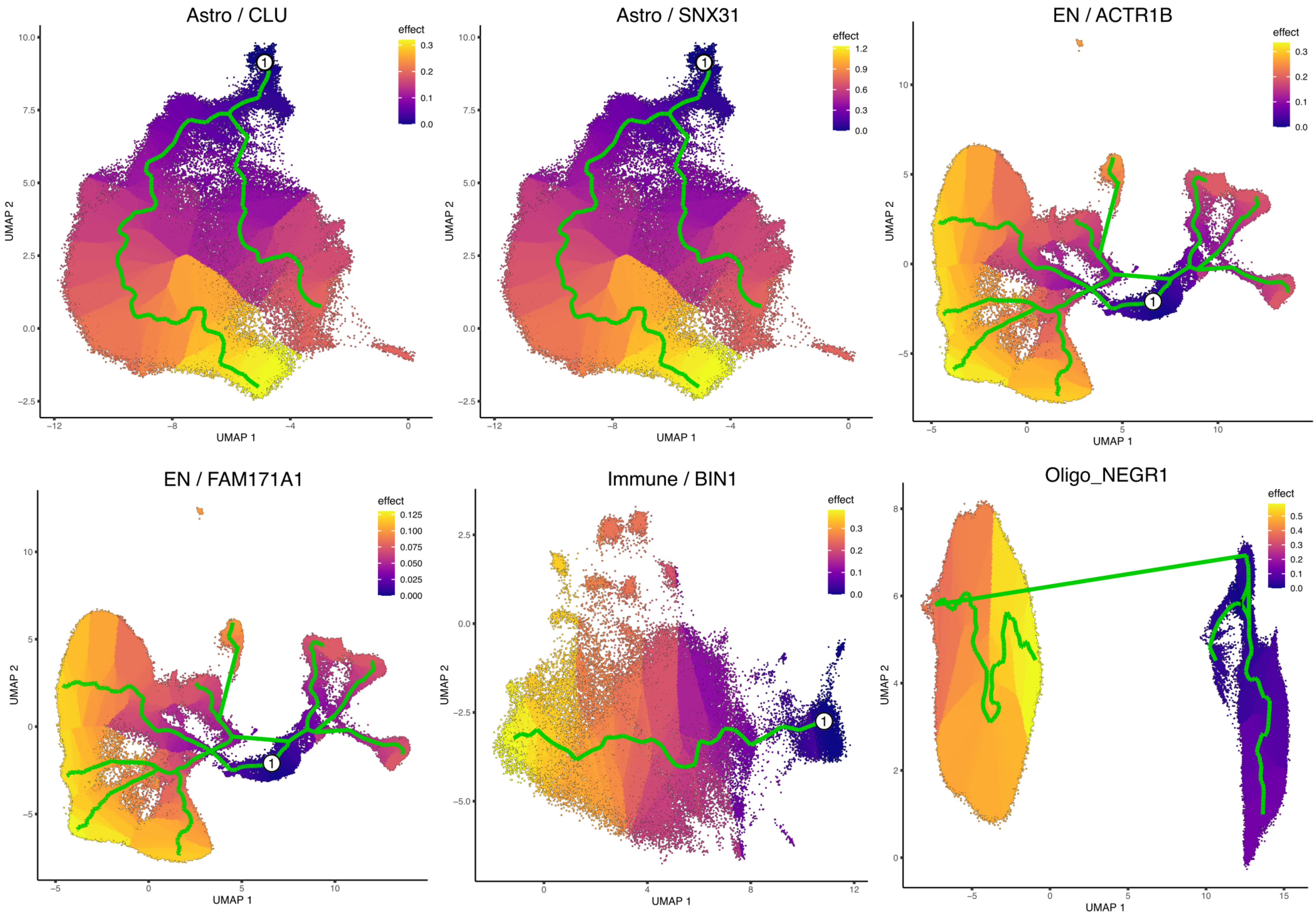


**Figure S18: Dynamic eQTLs at the class-level.** Supervised aging trajectories with cells colored by dynamic genetic effect changing over the pseudotime trajectory.

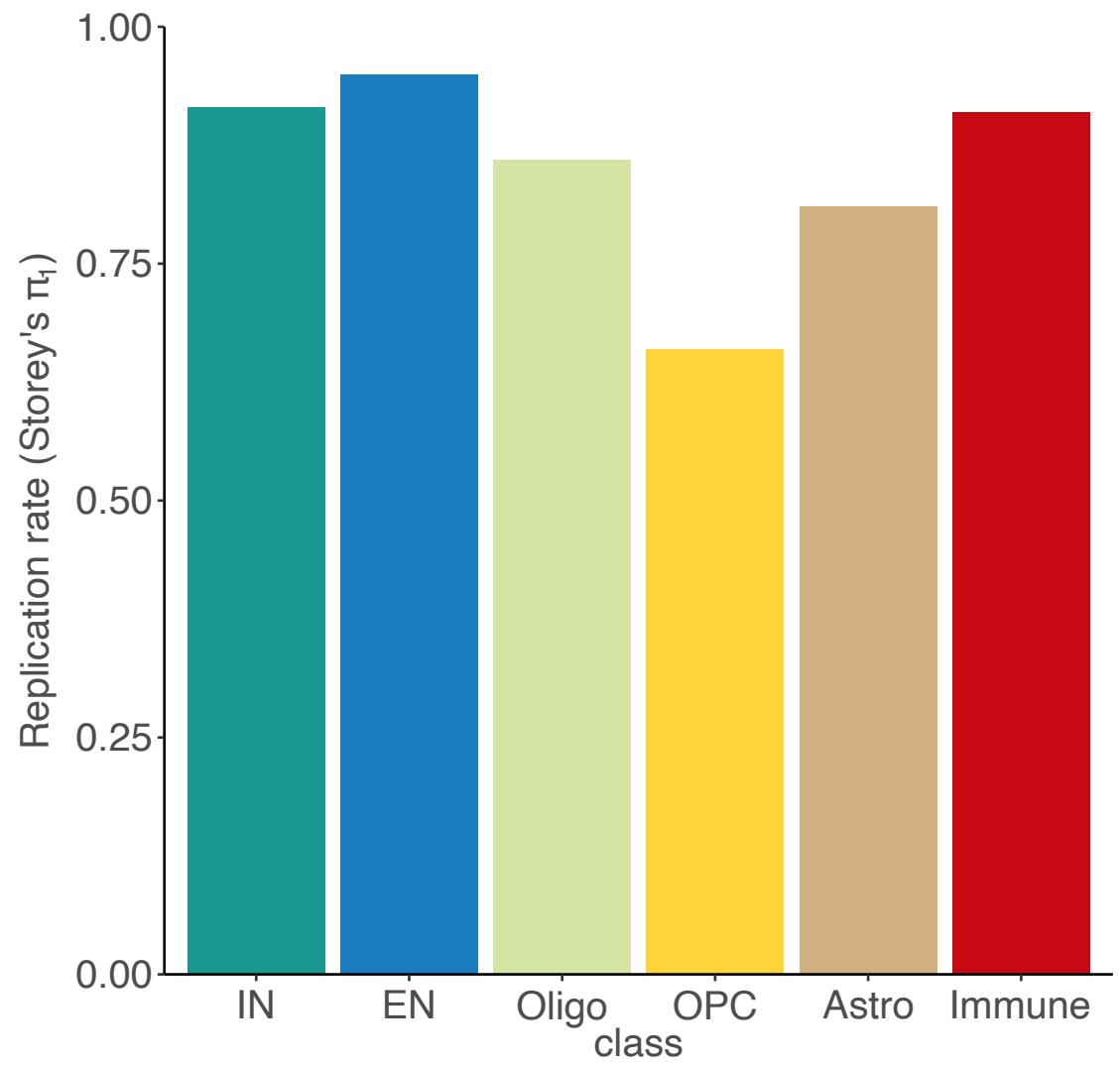


**Figure S19: Replication analysis of trans-QTLs in ROSMAP datasets.** Trans-eQTL analysis was performed in the ROSMAP dataset for significant trans-eQTLs discovered in the PsychAD dataset. The p-values from the ROSMAP dataset were extracted, and Storey's $\pi_1$ was used to estimate the replication rate.

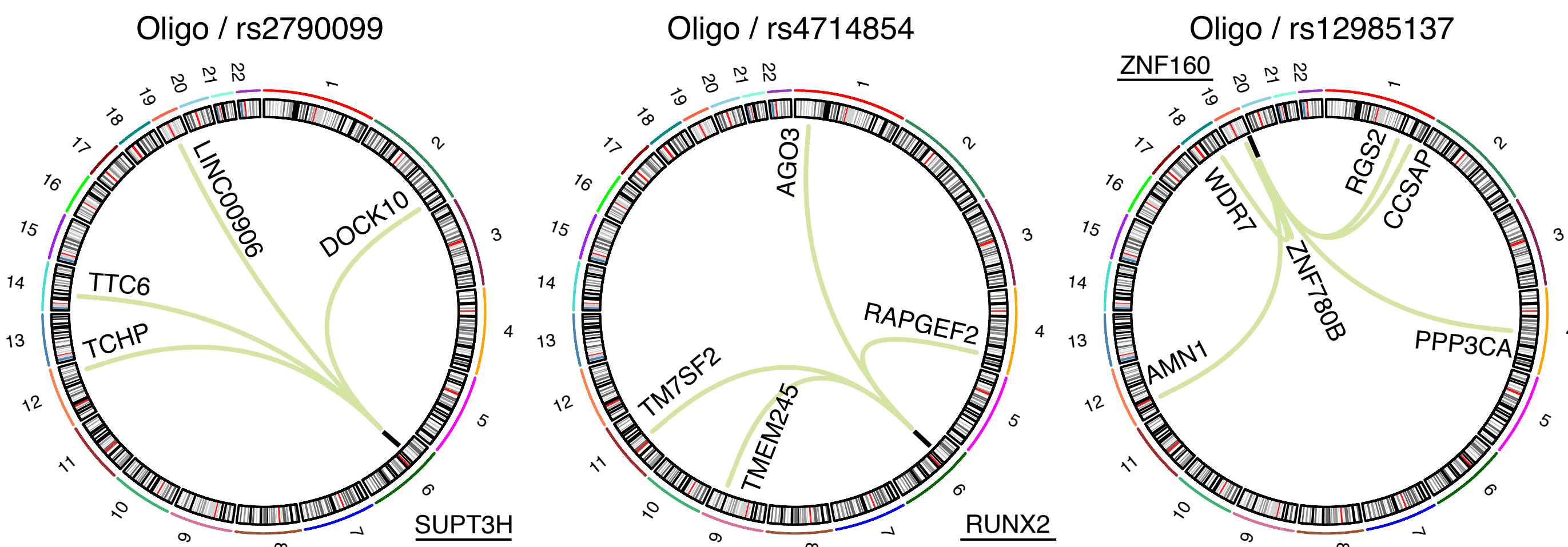


**Figure S20: Trans-regulatory hubs.** Trans-regulatory hubs with more than 2 downstream target genes at a study-wide FDR of 5% were identified only in oligodendrocytes. Underlined genes indicate cis-eQTLs

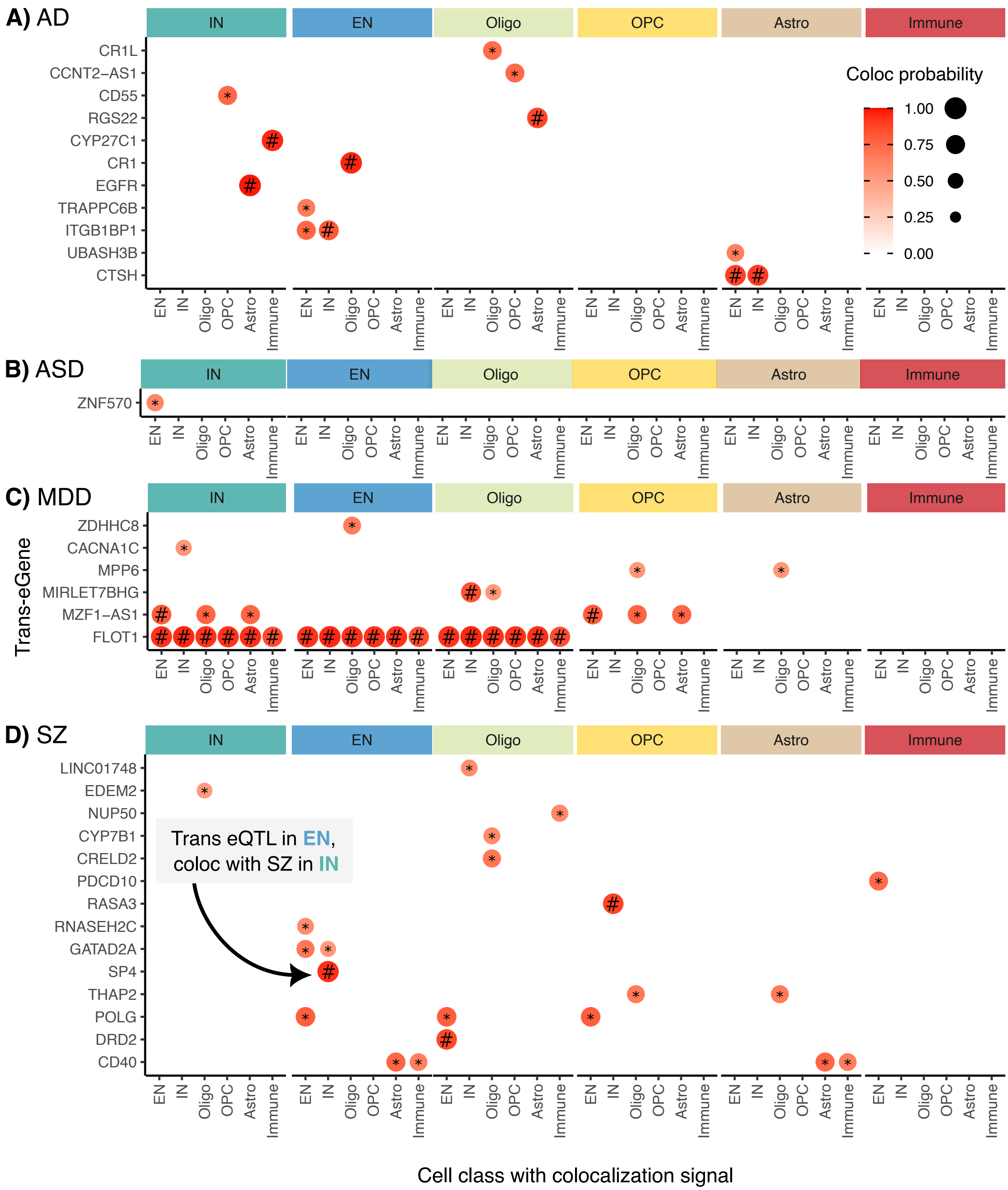


**Figure S21: Genes with a trans eQTL and a disease colocalization signal.** Genes with a trans eQTL (shown in inset of each cell class) that have a disease colocalization signal (shown on x-axis). For example, the gene SP4 has a trans eQTL detected in EN and regulatory variants for this gene colocalize with SZ risk in IN. Color and size of circle indicates posterior probability from colocalization analysis. Results are shown for **A**) AD and **B**) ASD **C**) MDD and **D**) SZ. '#' indicates colocalization probability $> 0.8$, and '*' indicates colocalization probability $> 0.5$.

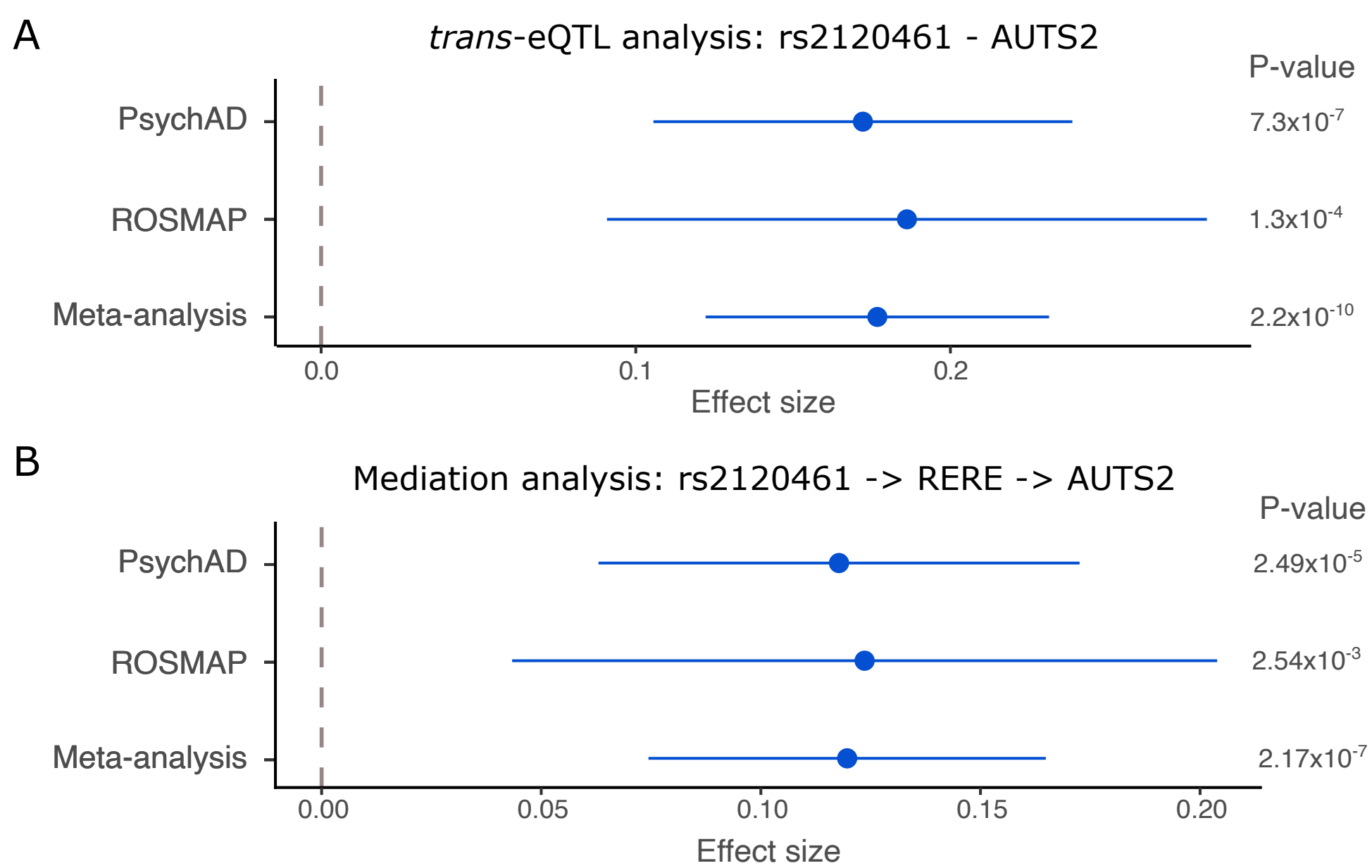


**Figure S22: Replication of trans-eQTL and mediation analysis for AUTS2. A**) Trans-eQTL analysis for rs2120461 and AUTS2 expression. **B**) Mediation analysis testing the model where RERE expression mediates the effect of rs2120461 on AUTS2 expression. Effect sizes, 95% confidence intervals, and p-values are shown for PsychAD and ROSMAP cohorts and fixed effects meta-analysis.

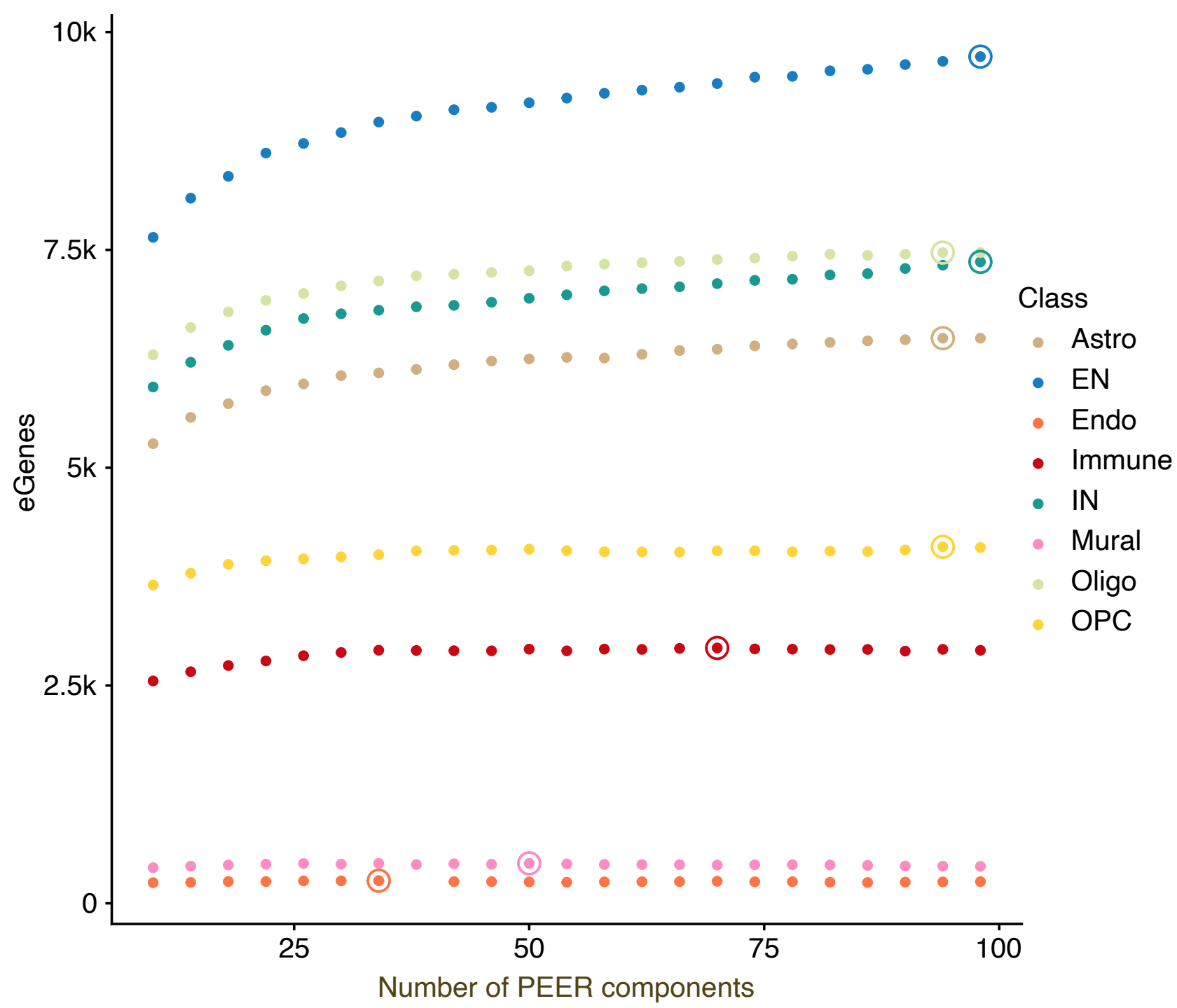


**Figure S23: Optimization of PEER factor inclusion for QTL discovery.** Relationship between the number of PEER factors included (x-axis) and the number of detected eGenes (y-axis) for each cell class. Circles indicate the number of PEER components selected for each cell class.

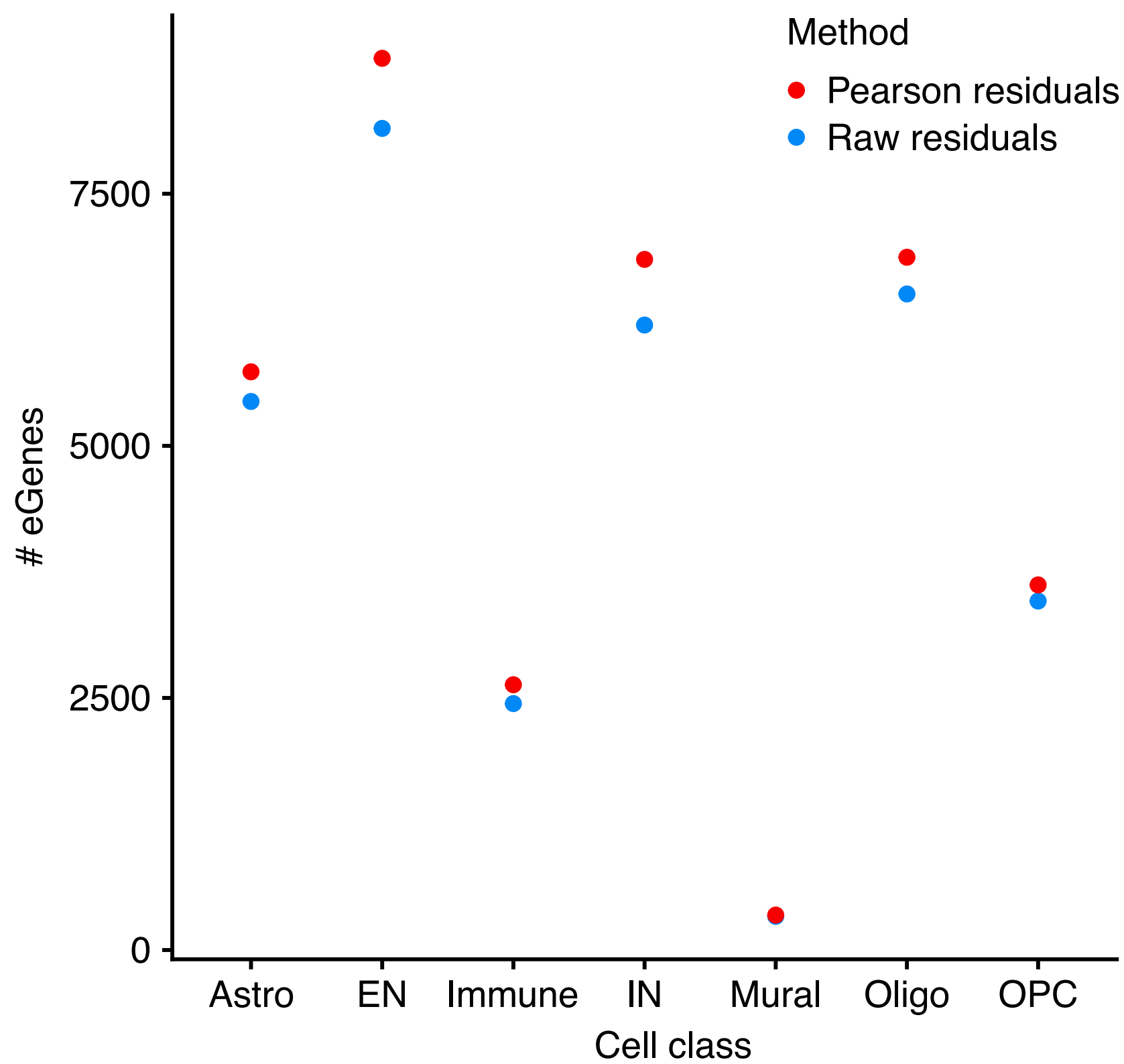


**Figure S 24: Comparison of normalization strategies for QTL detection across cell types.** We compare the number of genome-wide significant eGenes detected using Pearson residuals (as in the main analysis) to those detected using raw residuals after accounting for covariates from a precision-weighted regression model. For each method, QTL analysis is performed independently in eight cell classes using an identical statistical framework and significance threshold. The number of genome-wide significant eGenes detected per cell class is shown for each normalization strategy.

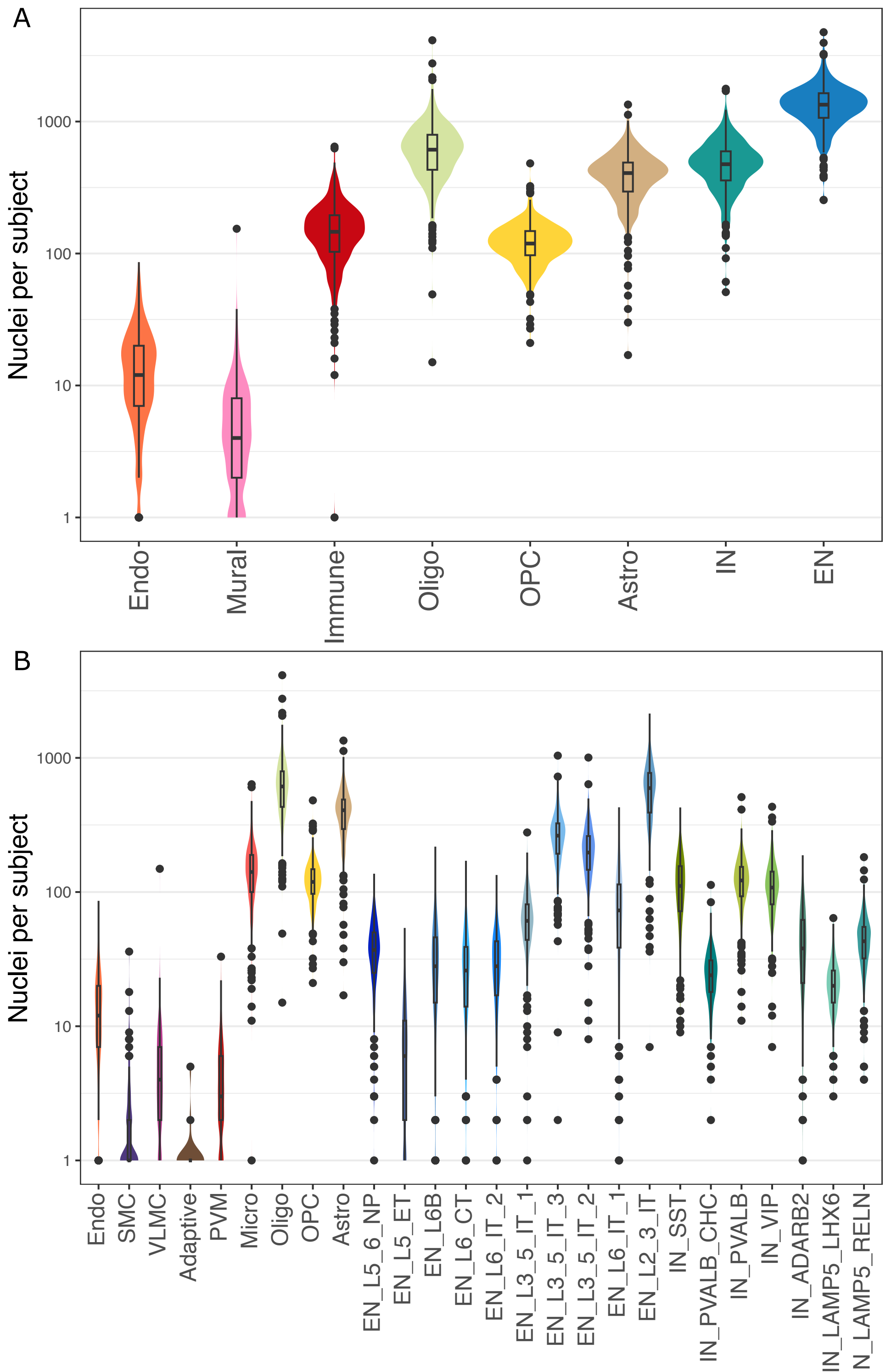


**Figure S25: Number of nuclei per subject for ROSMAP cohort.** Counts shown for **A**) class and **B**) subclass.

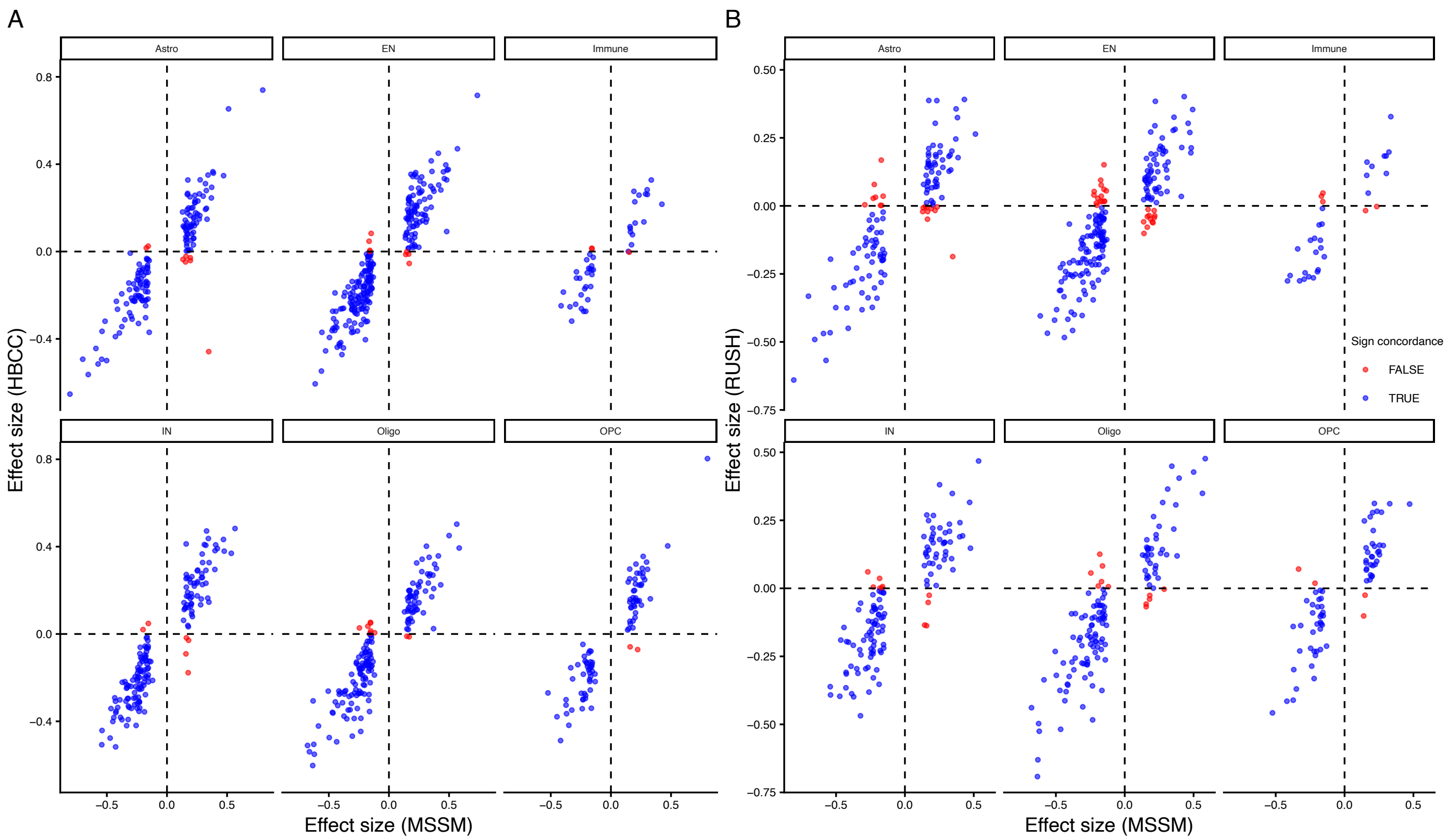


**Figure S26: Effect size concordance across brain banks for X chromosome.** Estimated eQTL effect sizes are shown for 3 brain banks for the lead variant from each gene with a genome-wide significant eQTL detected in the MSSM cohort. Points are colored by sign concordance with blue indicating the same sign in both brain banks and red indicating different signs. **A**) Effect sizes from HBCC shown against eQTLs discovered in MSSM. **B**) Effect sizes from RADC shown against eQTLs discovered in MSSM.

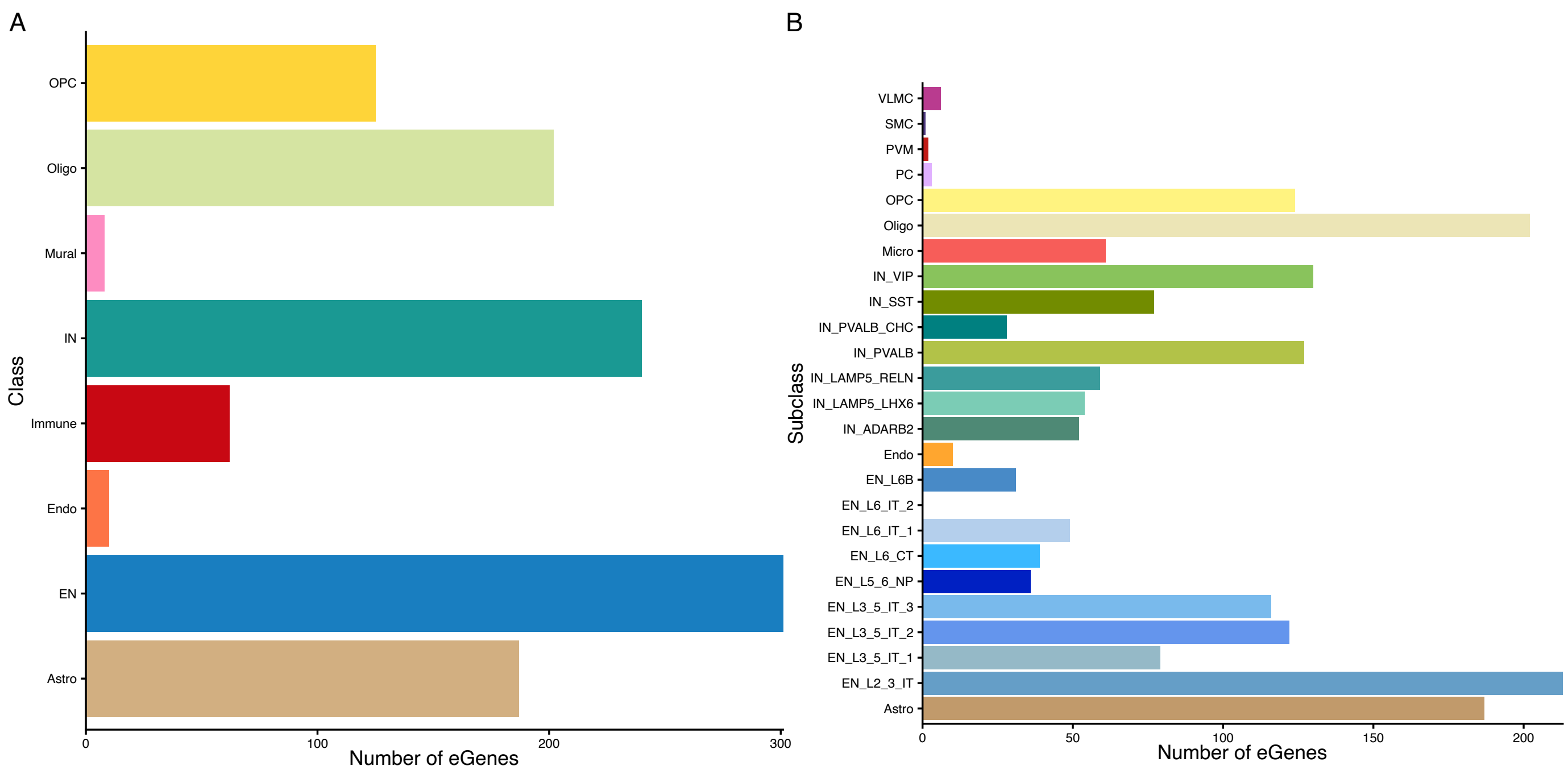


**Figure S27: Number of eGenes discovered in the X chromosome.** Number of genome-wide significant eGenes on chrX at the **A**) class and **B**) subclass levels.
