# Supplementary material for: Single-nucleus atlas of cell-type specific genetic regulation in the human brain
Source: Nat Genet. 2026 Sep 23;58(10):2525–34. doi: 10.1038/s41588-026-02733-5 (PMC13649572; doi:10.1038/s41588-026-02733-5)
Supplement: Supplementary file 3 [file 41588_2026_2733_MOESM3_ESM.pdf]

nature portfolio

Peer Review File

Single-Nucleus Atlas of Cell-Type Specific Genetic Regulation in the Human Brain

Corresponding Author: Dr Gabriel Hoffman

Version 0:

Decision Letter:

7th Jan 2026

Dear Dr Hoffman,

Your Article, "Single-Nucleus Atlas of Cell-Type Specific Genetic Regulation in the Human Brain" has now been seen by 3 referees. I apologize for the delay in getting back to you. I had to replace two of the original reviewers. You will see from their comments copied below that while they find your work of considerable potential interest, they have raised quite substantial concerns that must be addressed. In light of these comments, we cannot accept the manuscript for publication, but would be very interested in considering a revised version that addresses these serious concerns.

We hope you will find the referees' comments useful as you decide how to proceed. If you wish to submit a substantially revised manuscript, please bear in mind that we will be reluctant to approach the referees again in the absence of major revisions.

To guide the scope of the revisions, the editors discuss the referee reports in detail within the team, including with the chief editor, with a view to identifying key priorities that should be addressed in revision and sometimes overruling referee requests that are deemed beyond the scope of the current study. We hope that you will find the prioritised set of referee points to be useful when revising your study. Please do not hesitate to get in touch if you would like to discuss these issues further.

In particular, we ask that you address reviewer 4's requests for clarification of the methods, and concerns with the normalization and peer factors.

If you choose to revise your manuscript taking into account all reviewer and editor comments, please highlight all changes in the manuscript text file. At this stage we will need you to upload a copy of the manuscript in MS Word .docx or similar editable format.

We are committed to providing a fair and constructive peer-review process. Do not hesitate to contact us if there are specific requests from the reviewers that you believe are technically impossible or unlikely to yield a meaningful outcome.

If revising your manuscript:

*1) Include a “Response to referees” document detailing, point-by-point, how you addressed each referee comment. If no action was taken to address a point, you must provide a compelling argument. This response will be sent back to the referees along with the revised manuscript.

*2) If you have not done so already please begin to revise your manuscript so that it conforms to our Article format instructions, available here.
Refer also to any guidelines provided in this letter.

*3) Include a revised version of any required Reporting Summary: https://www.nature.com/documents/nr-reporting-summary.pdf
It will be available to referees (and, potentially, statisticians) to aid in their evaluation if the manuscript goes back for peer review.
A revised checklist is essential for re-review of the paper.

Please be aware of our guidelines on digital image standards.

EXTENDED DATA FIGURES

When re-submitting your manuscript, please ensure that any supplementary figures and tables that are crucial to the manuscript's conclusions are converted into Extended Data figures and tables to increase visibility of these data. Extended Data figures and tables are online-only (present in the online PDF and full-text HTML versions of the paper), peer-reviewed display items that provide essential background to the article but are not included in the main article due to space constraints. A maximum of ten Extended Data display items (figures and tables) is permitted.

You may use the link below to submit your revised manuscript and related files:

Link Redacted

Note: This URL links to your confidential home page and associated information about manuscripts you may have submitted, or that you are reviewing for us. If you wish to forward this email to co-authors, please delete the link to your homepage.

If you wish to submit a suitably revised manuscript we would hope to receive it within 6 months. If you cannot send it within this time, please let us know. We will be happy to consider your revision so long as nothing similar has been accepted for publication at Nature Genetics or published elsewhere. Should your manuscript be substantially delayed without notifying us in advance and your article is eventually published, the received date would be that of the revised, not the original, version.

Please do not hesitate to contact me if you have any questions or would like to discuss the required revisions further.

Nature Genetics is committed to improving transparency in authorship. As part of our efforts in this direction, we are now requesting that all authors identified as 'corresponding author' on published papers create and link their Open Researcher and Contributor Identifier (ORCID) with their account on the Manuscript Tracking System (MTS), prior to acceptance. ORCID helps the scientific community achieve unambiguous attribution of all scholarly contributions. You can create and link your ORCID from the home page of the MTS by clicking on 'Modify my Springer Nature account'. For more information please visit please visit www.springernature.com/orcid.

Thank you for the opportunity to review your work.

Sincerely,

Margot Brandt, PhD
Senior Editor
Nature Genetics
https://orcid.org/0000-0002-9434-794X

Referee expertise:

Referee #1: computational genomics

Referee #4: sc eQTL

Referee #5: statistical genetics and NDD

Reviewers' Comments:

Reviewer #1 (Remarks to the Author):

The authors have adequately address my comments.

Reviewer #4 (Remarks to the Author):

Note that this reviewer was not part of the previous round of revision.

Zeng et al. performed the largest single-cell eQTL study in the human brain to date, utilizing novel data from 1,384 donors across diverse ancestries. Given the scale and scope of this dataset, this study has the potential to be a significant resource for the community and for understanding the genetics of complex brain traits. They report genetic regulation for 14,258 genes, with 857 showing cell-type-specific effects.

While I appreciate the magnitude of the data collection, I have substantial concerns regarding the sensitivity of the analysis,

the transparency of the methods, and the robustness of the results, which currently limit the study's impact.

My major concerns are as follows:

Sensitivity and Replication: The number of detected eGenes is unexpectedly low compared to smaller studies, potentially due to the low number of PEER factors used relative to the sample size, possible non-optimal gene expression normalization, or poor quality control on the single nuclei data. Furthermore, the replication rate of co-localization results in ROSMAP is concerningly low (~20% at PP > 0.8), raising questions about the robustness of the detected signals.

Methodological Opacity: Key analytical steps are not described in sufficient detail to allow for reproducibility or proper interpretation. This includes the fine-mapping approach, colocalization parameters (windows/thresholds), and the S-LDSC analysis.

Single-Cell QC: There is no description of how ambient RNA was handled. Without rigorous ambient RNA removal, claims regarding cell-type-specific regulatory effects (and downstream colocalization) are difficult to validate.

Analytical Inconsistencies: There are contradictions in the text regarding normalization (e.g., Dreamlet residuals vs. Log2 CPM) and instances where the interpretation does not match the data presented (e.g., EGFR colocalization in oligodendrocytes vs. astrocytes).

Reproducibility: Essential demographic tables, as well as key supplementary tables summarising the results of this study are missing. In addition, the provided code repository appears incomplete (lacking scripts for colocalization calculations for example).

Overall, while the dataset is impressive, the manuscript requires significant improvement in methodological description, quality control, and downstream interpretation to fully support its claims.

Major Comments:

1. Data Processing, Quality Control, and Sensitivity
Ambient RNA Removal: The authors state they used a "stringent QC process to eliminate ambient RNA," but there is no description of the specific method or code used. I reviewed the companion paper (Lee et al.) and similarly found no description. Given the focus on cell-type specificity, ambient RNA contamination is a critical confounder. Please detail the methodology used.

Normalization Strategy: The description of gene expression normalization is confusing and potentially contradictory. The text mentions using dreamlet to construct pseudo-bulk values, which are then corrected for covariates, followed by dividing residuals by the standard deviation, but later, the authors also state that Log2(CPM) were used for the eQTL analysis. These are mathematically distinct. Additionally, if Log2(CPM) was used, how were CPM=0 values handled?

Sensitivity of Detection: The number of detected eGenes (e.g., 414 in endothelial cells) is substantially lower than expected for a study of this magnitude (N=1,384), especially when compared to smaller studies like SingleBrain (N=983) which detected >4,000 eGenes in the same cell type.
I suspect the number of PEER factors used may be too low for this large sample size.
Please provide a scatter plot showing the number of eQTLs detected vs. the number of PEER factors to justify the current selection (also with PEER factors well beyond 98, up to 500 may be reasonable).
The normalization approach used (standardised residuals or Log2(CPM)?) may also have an impact on the lower number of discoveries.
The QC metrics used for the eQTL mapping could also reduce power for the eQTL analysis. This should be described in more detail.

Replication Rates (Figure S8): At PP.H4>0.8, only ~20% of signals replicate in ROSMAP. This is concerningly low. Please verify if the high-confidence signals from the Fujita study replicate at a higher rate in your dataset to rule out systematic power or processing issues in the current pipeline.

2. Statistical Methodologies and Fine-Mapping

Fine-Mapping Description: The text mentions that "fine-mapped regulatory variants in excitatory neurons showed the strongest association with allelic size," yet the fine-mapping methodology is barely described. Unclear parameters include:
Which software/algorithm was used?
What priors and settings were applied?
Number of fine-mapped signals per gene and average size of the 95% credible set?
Please provide a caption for Figure S4 and clarify the methods to allow interpretation of Figure 2A and Figure S5.

Stratified LD-Score Regression (S-LDSC): The description is insufficient.
Which baseline model was used (Finucane 2015 vs. Gazal 2017)?
Was significance computed based on heritability enrichment p-values or coefficient z-scores? Authors should use coefficient p-values to account for the baseline model.
Are cell-type-specific fine-mapped variants enriched? It looks like the authors used fine-mapped variants in a cell type

regardless of whether these are also fine-mapped in another cell type. (This may help distinguish true specificity from overlapping regulatory variants).

Heritability Mediation Analysis: This analysis is mentioned with only a citation to the methodology paper. It is currently unclear if this was performed based on all cis-eQTLs, fine-mapped cis-eQTLs, cis+trans eQTL or other criteria. Please expand the description in the methods and Figure S6.

Colocalization Methodology: The methods for colocalization are not reproducible as written.
Was it performed on fine-mapped GWAS (or eQTL) signals, windows centered on the GWAS index SNP, or windows centered on eQTL hits?
What GWAS p-value threshold was used? I noted several loci (e.g., SERPINB1, GALNT6) that are not genome-wide significant in the Bellenguez et al. (2022) AD GWAS. If a lower threshold (e.g., 5e-8) was used for candidate loci, this must be explicitly stated and justified. The specific GWAS studies used for the co-localization analysis are also missing from the text.

3. Interpretation of Results and Specific Loci
Read Depth vs. Abundance: The authors link eGene detection to cell abundance and read count (per nucleus). It would be valuable to disentangle these variables. What is the beta coefficient for reads-per-nucleus vs. cell abundance in a linear regression framework? Would "reads per sample" or “counts per sample” (aggregate of all nuclei in a cell type) be a better predictor?
Co-localization Inconsistencies:

EGFR: The discussion states the EGFR signal co-localizes with AD risk exclusively in astrocytes at one point, then exclusively in oligodendrocytes later on. The data appears to show colocalization in astrocytes, not oligodendrocytes. Please correct or clarify.

Multiple Genes at Loci: The authors claim only 2 genes share a locus, but I identified several others (e.g., EGFR/EGFR-AS1, CR1/CR2, BIN1/CYP27C1). This impacts GWAS-eQTL interpretation and should be visualized/discussed.

Figure 4F: Several highlighted genes (e.g., TRIM24, NDUFAF6) do not appear in Figure 2D (GWAS-eQTL colocalization). If these do not colocalize with AD GWAS, why are they highlighted in this context?
Co-localization follow-ups:
Is there any functional enrichment in cell type specific co-localization results? For example, are the immune AD co-localized genes enriched in different functions compared to the oligodendrocyte AD co-localized genes?

Trans-eQTLs (Figures S16, S18):
Are the trans-eQTLs also cis-eQTLs or GWAS SNPs? Any enrichment for one category vs the other?
Figure S18: Please add a supplementary table clarifying the index SNP ID, cis-gene, trans-gene, cis cell type, trans cell type, and colocalization probabilities. The current display makes it difficult to distinguish if the gene on the left is the cis-gene or the trans-gene and both are needed for proper interpretation of the results.

Statement on SZ Risk: The claim that RERE and AUTS2 colocalize with SZ risk should be toned down, as the probabilities (0.7 and 0.68) are not very high and below what is typically considered significant.

4. Reproducibility and Code
Demographics: Basic demographic tables (age, sex, etc.) for the 1,384 donors are missing. Referring to a companion paper is insufficient for evaluating the specific samples used here. Especially as the companion paper did not need the genotype information and, it’s reasonable to expect that not 100% of the samples have both snRNA-seq data and genotype data.
Code Availability: The provided GitHub repository appears incomplete. I could find plotting scripts but not the scripts for calculating colocalization or the QC steps done on the single cell data for example.
Missing Supplementary Data: Supplementary tables are missing and necessary (e.g., number of samples per cohort, demographics per study, eQTLs detected, co-localization results, SNP ids of best eQTL per gene for each cell type, etc…). There are a number of files on synapse but these are not well described and may not always be available.
The authors need to point out the location of the raw data (even if controlled access). This is necessary both for reproducibility purposes and for enabling future meta-analysis of human brain single cell eQTL data.

Minor Comments:

Structure/Flow: The results section mentions fine-mapping results (association with allelic size) before the fine-mapping methodology or concept is introduced. A brief introduction to the fine-mapping approach and results earlier in the text would improve readability.

Figure 3A Visualization: It would be informative to highlight the cell-type specificity of the colocalization signals directly in the figure. For example, explicitly labeling that EGFR colocalizes in astrocytes but not oligodendrocytes, or clarifying if INPP5D co-localization signals are in microglia, astrocytes, or both.

Figure S16 (Trans-eQTLs): A scatterplot similar to the cis-eQTL plot would be informative here to visualize whether trans-eQTLs are more cell-type specific than cis-eQTLs.

Trans-eQTL Selection: Please clarify how the 56,204 eSNPs and 45,088 GWAS variants were selected for trans-eQTL detection. Was this based solely on a p-value threshold (e.g., $p < 1e-8$)? If so, please discuss how you accounted for LD structure to prevent high false-positive rates from loci with many significant SNPs compared to loci with low LD (and hence fewer SNPs and fewer statistical tests).

Trans-Regulatory Hubs: Regarding the three identified trans-regulatory hubs, do they affect biologically relevant genes in cis (e.g., Transcription Factors)?

Multiple Testing: The multiple testing procedure for eQTL mapping is unclear. Did the authors use Bonferroni correction for the best eQTL per gene followed by Benjamini-Hochberg across genes (as in Zeng et al., 2022)? Please clarify.

Were all genes tested for eQTL analysis? Or only the expressed ones? Or the ones with a variance above a specific threshold?
Is the sample size for the eQTL mapping the same for all cell types? It's quite common that some samples are dropped for rare cell types as they contain few (or no) nuclei from the rare population.

Reviewer #5 (Remarks to the Author):

The inclusion of the replication cohorts adequately addresses many of the concerns raised in the previous reviews.

Minor concerns or points of clarification remain:

Without functional validation, the authors should be careful to not overstate their findings that implicate previously unreported genes. The authors' inclusion of the replication cohort to strengthen confidence in results is a good initial step, but regardless, without other independent lines of evidence, the conclusions claiming novel should not be over-stated. One example of text that should be toned down accordingly is the following statement in the abstract: "novel cell type-specific genes implicated in Alzheimer's disease [...]".

Within the following text that was added within the Results: "Genome-wide, eQTL results were highly concordant with results from two single-nucleus datasets and had the highest concordance for matching cell types (Bryois et al. 2022; Fujita et al. 2024) (Fig S1)." please include a quantitative metric to back up the qualitative descriptor ("highly concordant") for precision and to improve clarity.
Similarly, please quantify the following qualitative statement that was added to the results: "Using stricter posterior probability cutoffs retains a substantial number of cell-type-specific effects (Figure S12)", specifically what is meant by "substantial number".

The authors explain how they restricted the analysis of trans-regulatory effects to the autosomes, but presumably the analysis of cis-effects was also carried out on chrX (it would be helpful to be explicit in the main text). In the partitioning heritability analysis, did the authors extend the tool to include chrX or did they only examine autosomal data?
Also for the partitioning heritability analysis, it would be important for the authors to note what they used as the LD reference, given that the tool requires LD information that matches the inputted summary statistics; what was used is not so straightforward/obvious given the genetic ancestry diversity of the eQTL data.
Finally, note the small typo in this section: the authors interchangeably use S-LDSR and S-LDSC in the main text and in the response to reviewers - best to be consistent to avoid confusion.

The authors' response to the Review 3's comment on investigation of ASE to validate the eQTL results is confusing. They respond that "the eQTL and ASE effects show significant concordance"; in the figure they provide, the p-value for the correlation is significant, but the r2 is close to 0. So the allelic effects and the eQTL effect sizes are significantly not-correlated?

Version 1:

Decision Letter:

9th Mar 2026

Dear Dr Hoffman,

Your Article, "Single-Nucleus Atlas of Cell-Type Specific Genetic Regulation in the Human Brain" has now been seen by 2 referees. You will see from their comments below that while they find your work of interest, some important points are raised. We are interested in the possibility of publishing your study in Nature Genetics, but would like to consider your response to these concerns in the form of a revised manuscript before we make a final decision on publication.

To guide the scope of the revisions, the editors discuss the referee reports in detail within the team, including with the chief editor, with a view to identifying key priorities that should be addressed in revision and sometimes overruling referee requests that are deemed beyond the scope of the current study. We hope that you will find the prioritized set of referee points to be useful when revising your study. Please do not hesitate to get in touch if you would like to discuss these issues

further.

Although reviewer 5 chose not to include any comments to the authors, they did express to the editors that they believe it is not acceptable to exclude the x chromosome from the analysis. Thus, we request that you include the x chromosome and resubmit the revised manuscript.

We therefore invite you to revise your manuscript taking into account all reviewer and editor comments. Please highlight all changes in the manuscript text file. At this stage we will need you to upload a copy of the manuscript in MS Word .docx or similar editable format.

We are committed to providing a fair and constructive peer-review process. Do not hesitate to contact us if there are specific requests from the reviewers that you believe are technically impossible or unlikely to yield a meaningful outcome.

When revising your manuscript:

*1) Include a “Response to referees” document detailing, point-by-point, how you addressed each referee comment. If no action was taken to address a point, you must provide a compelling argument. This response will be sent back to the referees along with the revised manuscript.

*2) If you have not done so already please begin to revise your manuscript so that it conforms to our Article format instructions, available
here.
Refer also to any guidelines provided in this letter.

*3) Include a revised version of any required Reporting Summary: https://www.nature.com/documents/nr-reporting-summary.pdf
It will be available to referees (and, potentially, statisticians) to aid in their evaluation if the manuscript goes back for peer review.
A revised checklist is essential for re-review of the paper.

Please be aware of our guidelines on digital image standards.

EXTENDED DATA FIGURES

When re-submitting your manuscript, please ensure that any supplementary figures and tables that are crucial to the manuscript’s conclusions are converted into Extended Data figures and tables to increase visibility of these data. Extended Data figures and tables are online-only (present in the online PDF and full-text HTML versions of the paper), peer-reviewed display items that provide essential background to the article but are not included in the main article due to space constraints. A maximum of ten Extended Data display items (figures and tables) is permitted.

Please use the link below to submit your revised manuscript and related files:

Link Redacted

Note: This URL links to your confidential home page and associated information about manuscripts you may have submitted, or that you are reviewing for us. If you wish to forward this email to co-authors, please delete the link to your homepage.

We hope to receive your revised manuscript within four to eight weeks. If you cannot send it within this time, please let us know.

Please do not hesitate to contact me if you have any questions or would like to discuss these revisions further.

Nature Genetics is committed to improving transparency in authorship. As part of our efforts in this direction, we are now requesting that all authors identified as ‘corresponding author’ on published papers create and link their Open Researcher and Contributor Identifier (ORCID) with their account on the Manuscript Tracking System (MTS), prior to acceptance. ORCID helps the scientific community achieve unambiguous attribution of all scholarly contributions. You can create and link your ORCID from the home page of the MTS by clicking on ‘Modify my Springer Nature account’. For more information please visit please visit www.springernature.com/orcid.

We look forward to seeing the revised manuscript and thank you for the opportunity to review your work.

Sincerely,

Margot Brandt, PhD
Senior Editor
Nature Genetics

https://orcid.org/0000-0002-9434-794X

Reviewers' Comments:

Reviewer #4 (Remarks to the Author):

The authors have adequately address my comments.

One minor point which has not been corrected. The discussion still states: "We highlight the example of EGFR, which has at least two distinct regulatory programs, with one active in astrocytes and another in oligodendrocytes. Notably, only the regulatory signal in oligodendrocytes colocalizes with the genetic risk of AD, highlighting the importance of cell type-specific regulatory programs in disease biology."

The data shows that it's the astrocyte regulatory signal which co-localize with Alzheimer.

Version 2:

Decision Letter:

Our ref: NG-A69990R1

1st Jun 2026

Dear Dr. Hoffman,

Thank you for submitting your revised manuscript "Single-Nucleus Atlas of Cell-Type Specific Genetic Regulation in the Human Brain" (NG-A69990R1). It has now been seen by the original referees and their comments are below. The reviewers find that the paper has improved in revision, and therefore we'll be happy in principle to publish it in Nature Genetics, pending minor revisions to satisfy the referees' final requests and to comply with our editorial and formatting guidelines.

If the current version of your manuscript is in a PDF format, please email us a copy of the file in an editable format (Microsoft Word or LaTex)-- we can not proceed with PDFs at this stage.

We are now performing detailed checks on your paper and will send you a checklist detailing our editorial and formatting requirements soon. Please do not upload the final materials and make any revisions until you receive this additional information from us.

Thank you again for your interest in Nature Genetics Please do not hesitate to contact me if you have any questions.

Sincerely,

Margot Brandt, PhD
Senior Editor
Nature Genetics
https://orcid.org/0000-0002-9434-794X

Reviewer #5 (Remarks to the Author):

The addition of chromosome X presents a valuable resource for the community, and I thank the reviewers for this addition. I have no further comments.

Version 3:

Decision Letter:

In reply please quote: NG-A69990R2 Hoffman

30th Jul 2026

Dear Dr. Hoffman,

I am delighted to say that your manuscript "Single-Nucleus Atlas of Cell-Type Specific Genetic Regulation in the Human Brain" has been accepted for publication in an upcoming issue of Nature Genetics.

Over the next few weeks, your paper will be copyedited to ensure that it conforms to Nature Genetics style. Once your paper is typeset, you will receive an email with a link to choose the appropriate publishing options for your paper and our Author Services team will be in touch regarding any additional information that may be required.

After the grant of rights is completed, you will receive a link to your electronic proof via email with a request to make any corrections within 48 hours. If, when you receive your proof, you cannot meet this deadline, please inform us at rjsproduction@springernature.com immediately.

You will not receive your proofs until the publishing agreement has been received through our system.

Due to the importance of these deadlines, we ask that you please let us know now whether you will be difficult to contact over the next month. If this is the case, we ask you provide us with the contact information (email, phone and fax) of someone who will be able to check the proofs on your behalf, and who will be available to address any last-minute problems.

Your paper will be published online after we receive your corrections and will appear in print in the next available issue. You can find out your date of online publication by contacting the Nature Press Office (press@nature.com) after sending your e-proof corrections.

You may wish to make your media relations office aware of your accepted publication, in case they consider it appropriate to organize some internal or external publicity. Once your paper has been scheduled you will receive an email confirming the publication details. This is normally 3-4 working days in advance of publication. If you need additional notice of the date and time of publication, please let the production team know when you receive the proof of your article to ensure there is sufficient time to coordinate. Further information on our embargo policies can be found here: https://www.nature.com/authors/policies/embargo.html

Before your paper is published online, we shall be distributing a press release to news organizations worldwide, which may very well include details of your work. We are happy for your institution or funding agency to prepare its own press release, but it must mention the embargo date and Nature Genetics. Our Press Office may contact you closer to the time of publication, but if you or your Press Office have any enquiries in the meantime, please contact press@nature.com.

Acceptance is conditional on the data in the manuscript not being published elsewhere, or announced in the print or electronic media, until the embargo/publication date. These restrictions are not intended to deter you from presenting your data at academic meetings and conferences, but any enquiries from the media about papers not yet scheduled for publication should be referred to us.

Authors may need to take specific actions to achieve compliance with funder and institutional open access mandates. If your research is supported by a funder that requires immediate open access (e.g. according to Plan S principles or the NIH public access policy) then you should select the gold OA route, and we will direct you to the compliant route where possible. Because authors warrant under our subscription licensing terms that they haven't committed to licensing any version of their article under a licence inconsistent with the terms of our agreement – including the applicable embargo period – publication under the subscription model isn't suitable for authors whose funders require no embargo.

If you have any questions about our publishing options, costs, Open Access requirements, or our legal forms, please contact ASJournals@springernature.com

If you have posted a preprint on any preprint server, please ensure that the preprint details are updated with a publication reference, including the DOI and a URL to the published version of the article on the journal website.

To assist our authors in disseminating their research to the broader community, our SharedIt initiative provides you with a unique shareable link that will allow anyone (with or without a subscription) to read the published article. Recipients of the link with a subscription will also be able to download and print the PDF.

As soon as your article is published, you will receive an automated email with your shareable link.

You can now use a single sign-on for all your accounts, view the status of all your manuscript submissions and reviews, access usage statistics for your published articles and download a record of your refereeing activity for the Nature journals.

An online order form for reprints of your paper is available at https://www.nature.com/reprints/author-reprints.html. Please let your coauthors and your institutions' public affairs office know that they are also welcome to order reprints by this method.

If you have not already done so, we strongly recommend that you upload the step-by-step protocols used in this manuscript to protocols.io. protocols.io is an open online resource that allows researchers to share their detailed experimental know-

how. All uploaded protocols are made freely available and are assigned DOIs for ease of citation. Protocols can be linked to any publications in which they are used and will be linked to from your article. You can also establish a dedicated workspace to collect all your lab Protocols. By uploading your Protocols to protocols.io, you are enabling researchers to more readily reproduce or adapt the methodology you use, as well as increasing the visibility of your protocols and papers. Upload your Protocols at https://protocols.io. Further information can be found at https://www.protocols.io/help/publish-articles.

Sincerely,

Margot Brandt, PhD
Senior Editor
Nature Genetics
https://orcid.org/0000-0002-9434-794X

Click here if you would like to recommend Nature Genetics to your librarian
http://www.nature.com/subscriptions/recommend.html#forms

** Visit the Springer Nature Editorial and Publishing website at www.springernature.com/editorial-and-publishing-jobs for more information about our career opportunities. If you have any questions please click here.**

Open Access This Peer Review File is licensed under a Creative Commons Attribution 4.0 International License, which permits use, sharing, adaptation, distribution and reproduction in any medium or format, as long as you give appropriate credit to the original author(s) and the source, provide a link to the Creative Commons license, and indicate if changes were made.
In cases where reviewers are anonymous, credit should be given to 'Anonymous Referee' and the source.
The images or other third party material in this Peer Review File are included in the article's Creative Commons license, unless indicated otherwise in a credit line to the material. If material is not included in the article's Creative Commons license and your intended use is not permitted by statutory regulation or exceeds the permitted use, you will need to obtain permission directly from the copyright holder.
To view a copy of this license, visit https://creativecommons.org/licenses/by/4.0/

Response to Reviewers

We thank the reviewers for providing valuable feedback on our manuscript. Our responses are shown below in black, indented text. Changes to the manuscript are shown in red text.

Reviewer #1 (Remarks to the Author):

The authors have adequately address my comments.

Reviewer #4 (Remarks to the Author):

Note that this reviewer was not part of the previous round of revision.

Zeng et al. performed the largest single-cell eQTL study in the human brain to date, utilizing novel data from 1,384 donors across diverse ancestries. Given the scale and scope of this dataset, this study has the potential to be a significant resource for the community and for understanding the genetics of complex brain traits. They report genetic regulation for 14,258 genes, with 857 showing cell-type-specific effects.

While I appreciate the magnitude of the data collection, I have substantial concerns regarding the sensitivity of the analysis, the transparency of the methods, and the robustness of the results, which currently limit the study's impact.

My major concerns are as follows:

Sensitivity and Replication: The number of detected eGenes is unexpectedly low compared to smaller studies, potentially due to the low number of PEER factors used relative to the sample size, possible non-optimal gene expression normalization, or poor quality control on the single nuclei data. Furthermore, the replication rate of co-localization results in ROSMAP is concerningly low (~20% at PP > 0.8), raising questions about the robustness of the detected signals.

Methodological Opacity: Key analytical steps are not described in sufficient detail to allow for reproducibility or proper interpretation. This includes the fine-mapping approach, colocalization parameters (windows/thresholds), and the S-LDSC analysis.

Single-Cell QC: There is no description of how ambient RNA was handled. Without rigorous ambient RNA removal, claims regarding cell-type-specific regulatory effects (and downstream colocalization) are difficult to validate.

Analytical Inconsistencies: There are contradictions in the text regarding normalization (e.g., Dreamlet residuals vs. Log2 CPM) and instances where the interpretation does not match the data presented (e.g., EGFR colocalization in oligodendrocytes vs. astrocytes).

Reproducibility: Essential demographic tables, as well as key supplementary tables summarising the results of this study are missing. In addition, the provided code repository appears incomplete (lacking scripts for colocalization calculations for example).

Overall, while the dataset is impressive, the manuscript requires significant improvement in methodological description, quality control, and downstream interpretation to fully support its claims.

We thank the Reviewer for their time and valuable feedback. We included additional details and address each of these points below.

Major Comments:

1. Data Processing, Quality Control, and Sensitivity

Ambient RNA Removal: The authors state they used a "stringent QC process to eliminate ambient RNA," but there is no description of the specific method or code used. I reviewed the companion paper (Lee et al.) and similarly found no description. Given the focus on cell-type specificity, ambient RNA contamination is a critical confounder. Please detail the methodology used.

We thank the reviewer for raising this critical point regarding data quality control. We agree that ambient RNA contamination can be a significant confounder in single-cell analysis, and we apologize for the omission of these details in the original manuscript.

To address this, we have expanded our **Methods** section here and in the companion paper (Lee, et al.) to explicitly describe our approach:

We implemented a stringent QC process to eliminate ambient RNA and preserve high-quality nuclei for further analysis. As part of our rigorous quality control pipeline, we initially tested for potential contamination using CellBender[1], a deep generative model specifically designed to remove counts due to ambient RNA molecules and random barcode swapping. In our initial assessment, however, we did not observe significant ambient RNA contamination across our datasets. In addition, to ensure the highest fidelity of the final processed object and to robustly confirm that our cell-type specificity

results were not driven by background noise, we additionally performed an analysis using SoupX[2]. This secondary validation confirmed that our data is free of significant ambient RNA effects.

Normalization Strategy: The description of gene expression normalization is confusing and potentially contradictory. The text mentions using dreamlet to construct pseudo-bulk values, which are then corrected for covariates, followed by dividing residuals by the standard deviation, but later, the authors also state that Log2(CPM) were used for the eQTL analysis. These are mathematically distinct. Additionally, if Log2(CPM) was used, how were CPM=0 values handled?

We thank the reviewer for pointing out this issue. The previous language was ambiguous. We have updated the language to clarify that log2 CPM-transformed data were used to fit regression models, and that Pearson residuals from these models were then used in the QTL analysis.

The regression residuals used in the eQTL analysis were generated as follows. Expression for each cell type and each individual was computed as the $\log_2$ counts per million (CPM) with a pseudocount of 0.25 after aggregating all corresponding nuclei. A precision-weighted regression model was fit for each expressed gene in each cell type using the dreamlet package [3] using covariates for age, sex, postmortem interval, mitochondrial rate, ribosomal rate, and disease status. From these models, Pearson residuals (i.e., residuals divided by their standard errors) were computed for each expressed gene and cell type and used in downstream QTL analysis. Using the Pearson residuals identified 3.9 to 10.5% more genome-wide significant eGenes than using raw residuals (**Fig S24**).

Sensitivity of Detection: The number of detected eGenes (e.g., 414 in endothelial cells) is substantially lower than expected for a study of this magnitude (N=1,384), especially when compared to smaller studies like SingleBrain (N=983) which detected >4,000 eGenes in the same cell type.

I suspect the number of PEER factors used may be too low for this large sample size. Please provide a scatter plot showing the number of eQTLs detected vs. the number of PEER factors to justify the current selection (also with PEER factors well beyond 98, up to 500 may be reasonable).

The normalization approach used (standardised residuals or Log2(CPM)?) may also have an impact on the lower number of discoveries.

The QC metrics used for the eQTL mapping could also reduce power for the eQTL analysis. This should be described in more detail.

We appreciate this important point and have conducted several new analyses to address it.

PEER factor sensitivity

To find an optimal number of PEER factors to remove, we performed eQTL detection called on the input expression matrix normalized by pre-selected biological and technical covariates but differing in the numbers of PEER factors between 10 and 98 with an interval of 4. The setting with the most genome-wide significant eQTL detected was used as the final results for downstream analysis. We now include a new Supplementary Figure showing the number of detected eGenes as a function of PEER factors (ranging from 4 to 98). Consistent with previous large-sample eQTL studies, discovery rates plateaued at ~80–96 PEER factors.

The text now reads:

We applied the PEER package [4] to detect hidden unobserved covariates. To find an optimal number of PEER factors to remove, we performed eQTL detection on the input expression matrix, normalized by pre-selected biological and technical ~~covariates,~~ varying the number of PEER factors from 10 to 98 in increments of 4. The setting with the most genome-wide significant eQTLs detected was used as the final result for downstream analysis (**Fig. S23**).

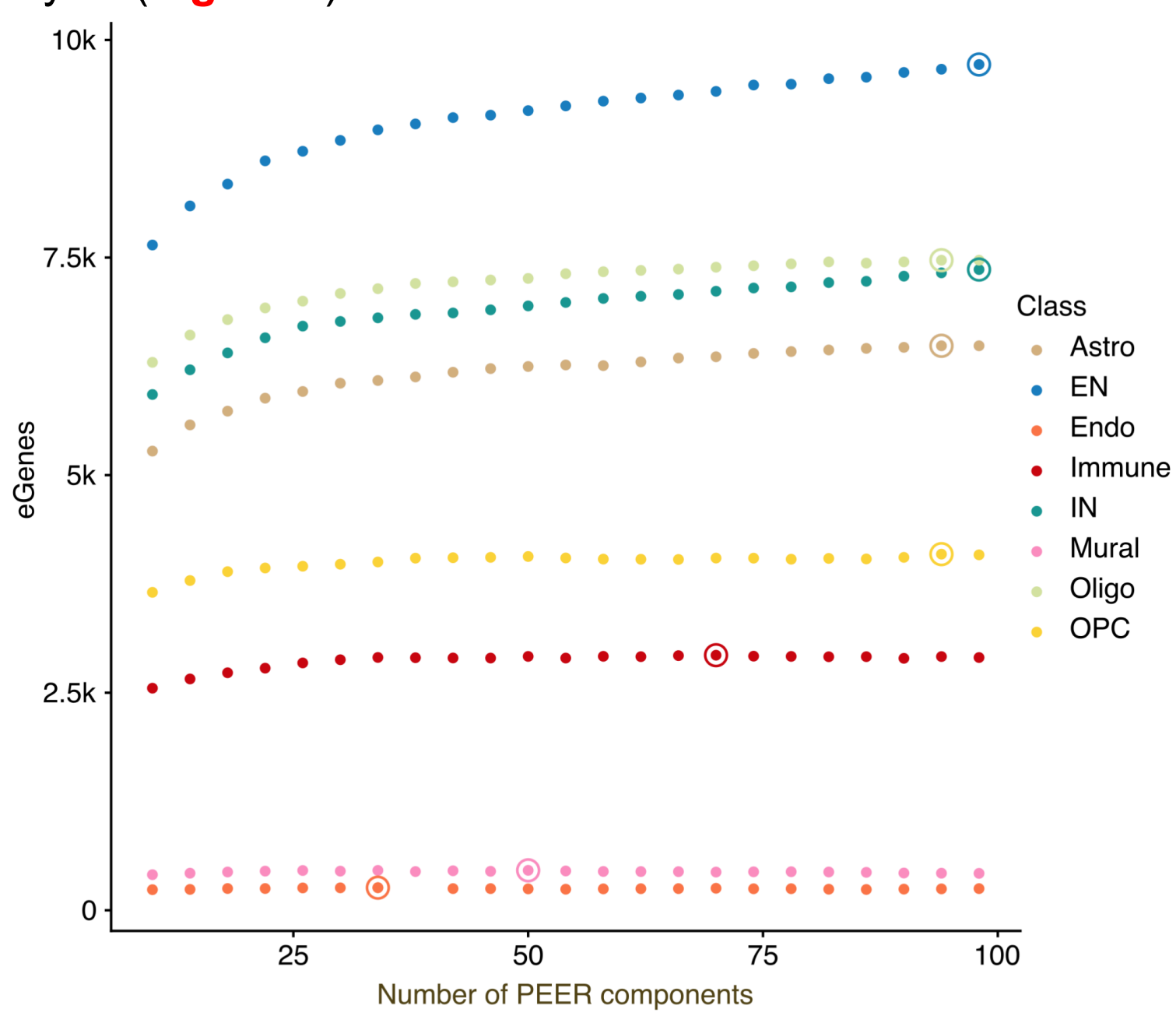

Fig S23. Optimization of PEER factor inclusion for QTL discovery. Relationship between the number of PEER factors included (x-axis) and the number of detected eGenes (y-axis) for each cell class. Circles indicate the number of PEER components selected for each cell class.

Normalization sensitivity:

We performed eQTL analysis using an alternative normalization strategy to compare the performance of Pearson residuals (as in the paper) with that of raw residuals after accounting for covariates. We observed high concordance in effect size estimates and found that using Pearson residuals yielded greater statistical power, identifying 3.9-10.5% more genome-wide significant eGenes.

The new text reads:

The regression residuals used in the eQTL analysis were generated as follows. Expression for each cell type and each individual was computed as the $\log_2$ counts per million (CPM) with a pseudocount of 0.25 after aggregating all corresponding nuclei. A precision-weighted regression model was fit for each expressed gene in each cell type using the dreamlet package [3] using covariates for age, sex, postmortem interval, mitochondrial rate, ribosomal rate, and disease status. From these models, Pearson residuals (i.e., residuals divided by their standard errors) were computed for each expressed gene and cell type and used in downstream QTL analysis. Using the Pearson residuals identified 3.9 to 10.5% more genome-wide significant eGenes than using raw residuals (**Fig S24**).

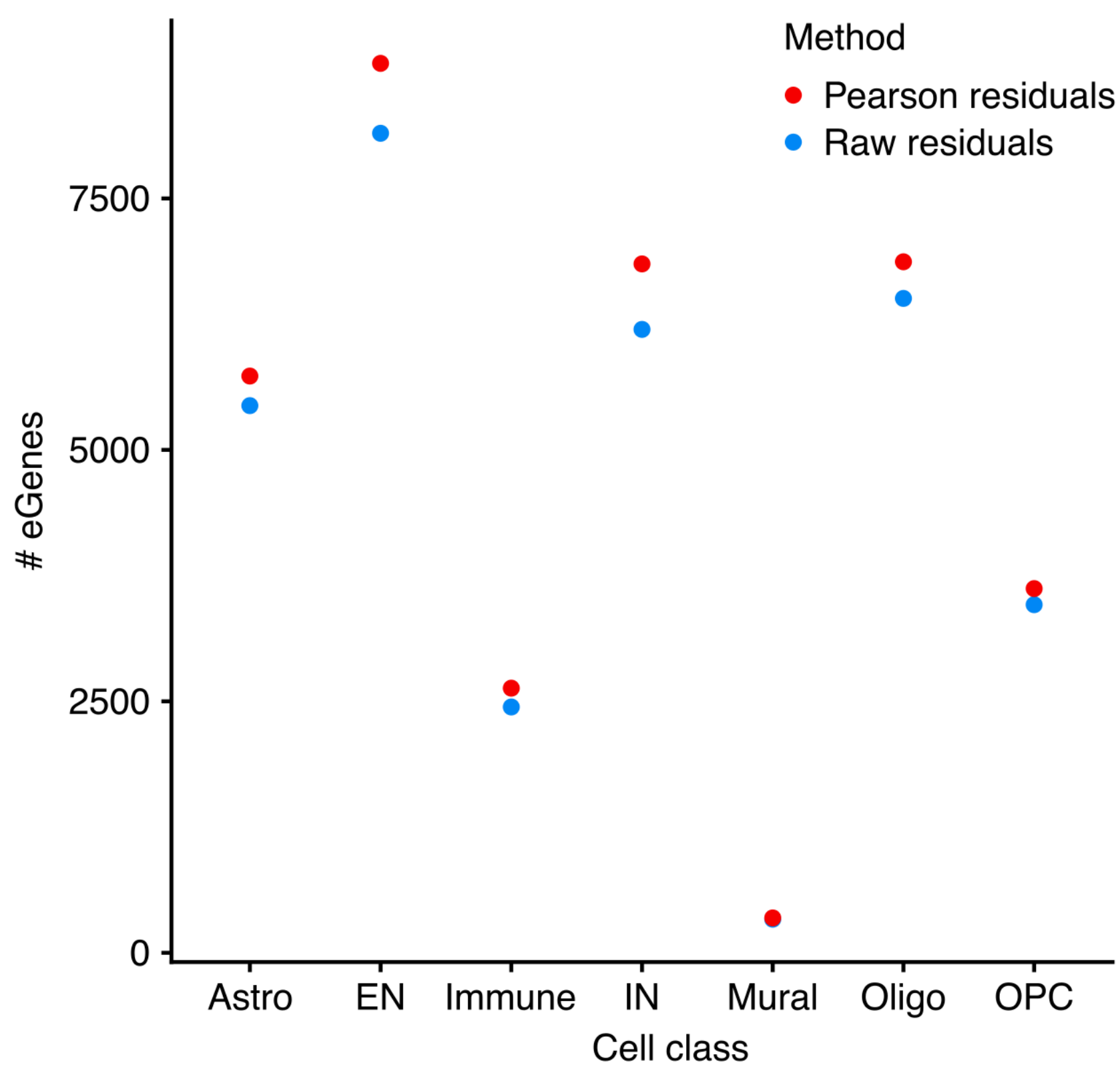

Fig S24. Comparison of normalization strategies for QTL detection across cell types. We compare the number of genome-wide significant eGenes

detected using Pearson residuals (as in the main analysis) to those detected using raw residuals after accounting for covariates from a precision-weighted regression model. For each method, QTL analysis is performed independently in eight cell classes using an identical statistical framework and significance threshold. The number of genome-wide significant eGenes detected per cell class is shown for each normalization strategy.

QC metrics:

We thank the reviewer for pointing out this issue. We have added more detail about the filtering of the gene expression and genotype data.

Analysis was performed at the pseudobulk level. For each cell type, donors with at least 5 nuclei were retained in order to ensure stable donor-level expression estimates and to reduce noise from sparse sampling. Genes were filtered within each cell type to retain only those that were robustly expressed. This was implemented using edgeR::filterByExpr() and required that at least 40% of donors have a minimum count of 5 reads per gene. After QC and filtering, the number of donors retained for each cohort varied (**Table S4**).

Imputed variants were filtered based on genotype completeness (95%), minor allele frequency (1%), and standard quality metrics (imputation INFO >0.3) to ensure high-confidence association testing. Donors were required to pass QC in both genotype and expression datasets.

Overall, the difference in eQTL discovery between this work and SingleBrain in endothelial cells is not attributable to PEER factors or normalization method. Instead, we suspect that the difference is due to subtle differences in gene expression filtering that have a large impact on rare cell types.

Endothelial cells contribute less than 1% of the cells in this study, and we observed that the eGenes detected are closely related to cell abundance (**Fig 1D**,**E**). Similarly, other rare cell types, like mural cells, have a similar number of eGenes detected. We note that in another single-cell eQTL study of human postmortem brains [5], only ~100 eGenes were detected, with sample sizes of 144 to 192. Compared to the SingleBrain study, our analysis applies more stringent donor-level QC and covariate correction, which likely explains part of the difference in raw eGene counts. For instance, in SingleBrain, 15,158 genes were determined to be expressed and explored for eQTL detection in Endothelial cells, while in our study, only 3,379 genes were detected as expressed.

We used a conservative expression cutoff in this work because we were concerned about an elevated false positive rate in genes with few counts. We expect that future work on QTL analysis of rare cell types using negative binomial

count models instead of precision weighted linear models can resolve this issue and control the false positive rate across a broader range of count magnitudes.

Replication Rates (Figure S8): At PP.H4>0.8, only ~20% of signals replicate in ROSMAP. This is concerningly low. Please verify if the high-confidence signals from the Fujita study replicate at a higher rate in your dataset to rule out systematic power or processing issues in the current pipeline.

We thank the reviewer for this suggestion and agree that replication rates provide an important diagnostic for potential power or processing issues. While replication analysis is common for eQTLs, there is far less work on the replication of colocalization signals. Therefore, we and the field have much less intuition about the expected replication rate.

To address this concern, we repeated the replication analysis using the ROSMAP cohort for discovery. Since the ROSMAP cohort has fewer samples, the colocalizations found here had a higher replication rate across all cutoffs than those in the larger PsychAD cohort. Indeed, we observed replication rates 21–29% higher when using ROSMAP as the discovery cohort.

These results indicate that the comparatively lower replication rate observed using PsychAD for discovery reflects asymmetries in statistical power and discovery context between datasets, rather than systematic issues with the current analysis pipeline.

We have updated **Fig S10** (previously, **Fig S8**) to include this new analysis:

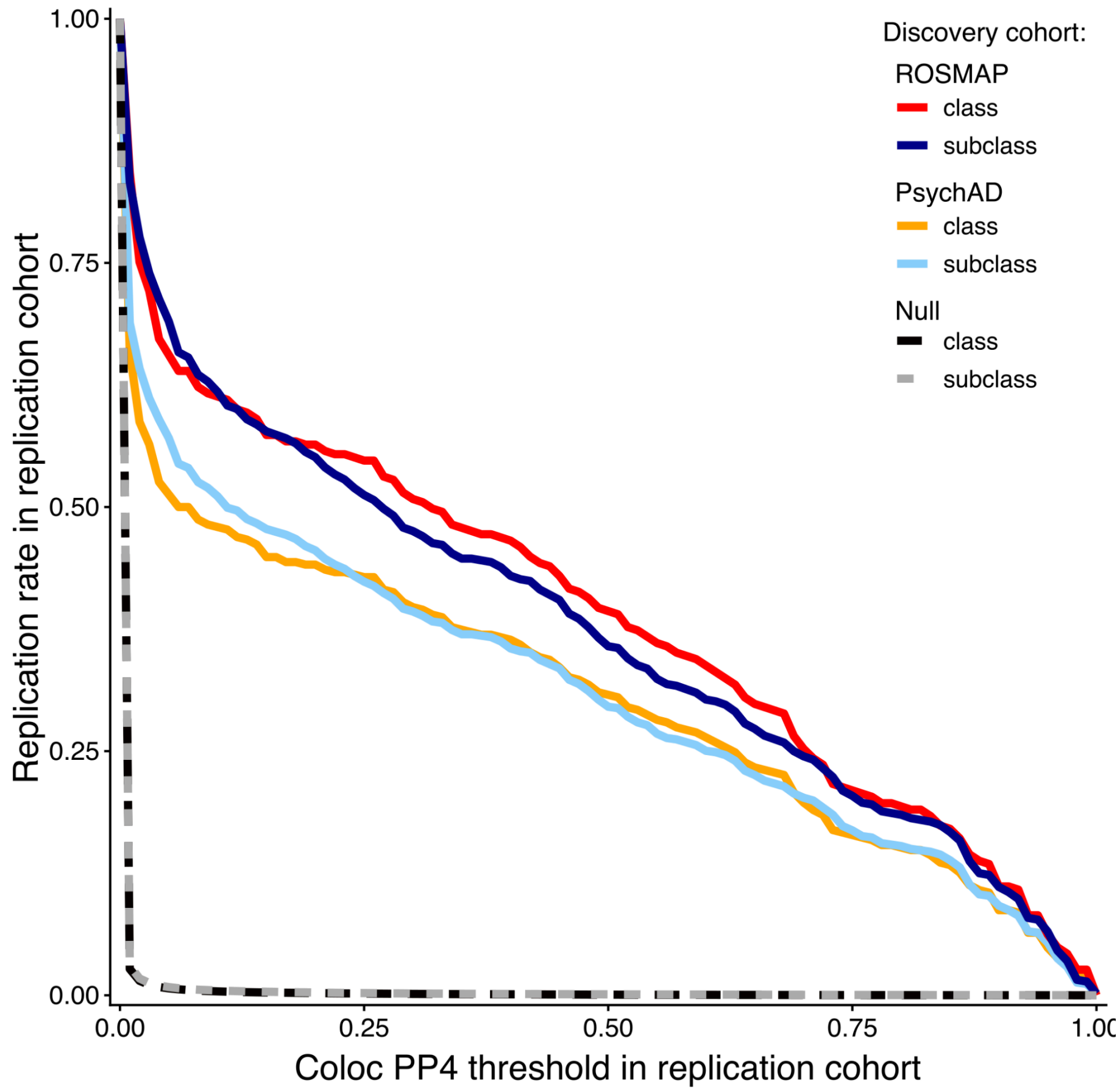

Figure S10. Replicating GWAS-eQTL colocalizations. For the discovery cohort, all GWAS-eQTL colocalization with PP4 > 0.8 discovered at the class and subclass levels were examined in the matching cell type in the replication cohort. The replication rate is calculated over a range of PP4 cutoffs from 0 to 1, so that the replication rate decreases as the cutoff becomes stricter. Each of PsychAD and ROSMAP were considered as the replication cohort, and the replication rate was evaluated in the other cohort across a range of PP4 values. Since the ROSMAP cohort has fewer samples, the colocalizations found here had a higher replication rate across all cutoffs than those in the larger PsychAD cohort. By randomly selecting a gene in ROSMAP and comparing its colocalization level with the PP4 threshold, the replication rate under the null hypothesis of no colocalization was assessed.

2. Statistical Methodologies and Fine-Mapping

Fine-Mapping Description: The text mentions that "fine-mapped regulatory variants in excitatory neurons showed the strongest association with allelic size," yet the fine-mapping methodology is barely described. Unclear parameters include:

Which software/algorithm was used?

What priors and settings were applied?

Number of fine-mapped signals per gene and average size of the 95% credible set?

Please provide a caption for Figure S4 and clarify the methods to allow interpretation of Figure 2A and Figure S5.

We thank the Review for raising this issue. We have added more detail about the statistical fine-mapping analysis in the Methods:

Fine-mapping of cis-eQTLs

We conducted fine-mapping with CAVIAR [6] (v2.0.0), which implements a probabilistic model to estimate posterior inclusion probabilities (PIPs) while accounting for local LD structure derived from study genotypes and assuming a single causal variant. We set the other parameters to their default values and output variants from 95% credible sets by ranking them by PIP until the cumulative posterior probability reaches 0.95. We reported the distribution of credible set sizes across loci, providing a quantitative summary of fine-mapping resolution.

We have added the following in the Results:

We then conducted fine-mapping to refine the set of candidate causal variants, focusing on cis-eQTL loci. Across all significant loci, the median 95% credible set size ranged from 10 to 22 variants at the class level, and 10 to 30 variants at the subclass level (**Table S1)**.

Table S1: Size of credible sets from statistical fine-mapping

Table_S1_Fine_mapping.xlsx

We thank the Review for pointing out the missing caption for **Fig S6** (previously **S4**). The caption now reads:

Integration with allelic effects from multiplexed parallel reporter assay (MPRA). A) The magnitude of allelic effects from MPRA increases with the fine-mapping posterior inclusion probability (PIP) from eQTL analysis. The best-fit line is shown in blue, and the gray band shows standard error. Although some SNPs are excluded from this plot because of the y-axis range, all SNPs were used to fit the regression model. **B**) Slope of lines from (A) showing the association between MPRA allelic effect size and PIP. Error bars indicate 95% confidence interval. P-value from hypothesis test comparing each estimate to zero is shown on the right.

Stratified LD-Score Regression (S-LDSC): The description is insufficient.
Which baseline model was used (Finucane 2015 vs. Gazal 2017)?
Was significance computed based on heritability enrichment p-values or coefficient z-scores? Authors should use coefficient p-values to account for the baseline model.
Are cell-type-specific fine-mapped variants enriched? It looks like the authors used fine-mapped variants in a cell type regardless of whether these are also fine-mapped in another cell type. (This may help distinguish true specificity from overlapping regulatory variants).

> We thank the Reviewer for raising these issues. We have included additional detail here:
>
> Stratified LD score regression (S-LDSC) was used to test if custom variant annotations from statistical fine-mapping of eQTL signals were enriched for heritability for genetic risk for complex traits [7,8]. Partitioned heritability analyses were conducted using only autosomal variants, consistent with standard S-LDSC analyses. For each eGene, statistical fine-mapping was used to compute a posterior inclusion probability for each *cis*-variant, retaining variants in the 95% credible set for each gene. Each variant in the genome is annotated with a probability value from this analysis. Variants not in a 95% credible set receive a value of 0, and variants evaluated for multiple genes receive the maximum probability value for the variant across these genes. This approach is termed 'MaxCPP' in Hormozdiari, et al. [8]. S-LDSC was then used to partition trait heritability using the constructed functional annotations, using the 1000 Genomes Phase 3 European ancestry reference panel. The estimated enrichment was used to measure the importance of each eQTL category to human complex traits or diseases. To rule out potential influences of correlation among eQTL categories, we aggregated the baselineLD model, which includes a set of 75 functional annotations from Gazal et al.[9], to create functional annotations for the eQTL category, then ran S-LDSC jointly and assessed significance using the enrichment p-value.
>
> Using the enrichment p-value is standard practice in our group and others [7,8,10–12], including the original developers, although others use the coefficient p-value.

Heritability Mediation Analysis: This analysis is mentioned with only a citation to the methodology paper. It is currently unclear if this was performed based on all cis-eQTLs, fine-mapped cis-eQTLs, cis+trans eQTL or other criteria. Please expand the description in the methods and Figure S6.

> We thank the reviewer for pointing this out. Per the package's instructions, we applied MeSC using all expressed genes.
>
> We have expanded the Methods to include:

Following the package's instructions, analysis was performed on all expressed genes. No additional filtering was performed based on cis-, trans-eQTLs results, fine-mapping, or other criteria.

The caption for the corresponding figure now reads:

Figure S8. Mediation of trait heritability. Fraction of heritability of complex traits mediated by regulatory variants at the **A**) class and **B**) subclass levels. Tests with FDR < 5% are indicated by 'X'. Analysis was performed on all expressed genes, with no additional filtering based on eQTL or fine-mapping.

Colocalization Methodology: The methods for colocalization are not reproducible as written.
Was it performed on fine-mapped GWAS (or eQTL) signals, windows centered on the GWAS index SNP, or windows centered on eQTL hits?
What GWAS p-value threshold was used? I noted several loci (e.g., SERPINB1, GALNT6) that are not genome-wide significant in the Bellenguez et al. (2022) AD GWAS. If a lower threshold (e.g., 5e-8) was used for candidate loci, this must be explicitly stated and justified. The specific GWAS studies used for the co-localization analysis are also missing from the text.

We thank the reviewer for raising this issue. We clarify that, consistent with previous work by our group [11], and others [13,14], no p-value thresholds were used, so it is possible to identify colocalization with a locus that does not reach genome-wide significance. Coloc is a Bayesian method to evaluate evidence supporting five different scenarios (**H0**, no causal variant in either trait; **H1**, only one causal variant in trait 1; **H2,** only one causal variant in trait 2; **H3**, two different causal variants in 2 traits; **H4**, one same causal variant in both traits). The final posterior probability is determined by QTL signals in both traits, so even including sub-genome-wide significant loci should not increase the false positive rate [15].

Although genes like *SERPINB1* and *GALNT6* don't reach genome-wide significance in [16], they were reported to be associated with AD in previous studies [17,18] and can be considered good candidates for future functional validation experiments.

We have revised the text to read:
Results:

Colocalization analysis of genetic signals shared between regulatory and risk variants identified genes involved in the molecular etiology of disease, including 46 genes in AD [16], 22 in MDD [19] and 46 in SZ [20] (**Fig 2B**).

<u>Methods:</u>
To evaluate the relationship between molecular QTL, we used the coloc R package to conduct colocalization analysis [15]. The summary results from meta-analysis were used as input for coloc. Colocalization was performed within overlapped regions between eQTL and GWAS summary statistics centered around the gene body. Consistent with previous work by our group [11], and others [13,14], no p-value thresholds were used, so it is possible to identify colocalization with a locus that does not reach genome-wide significance. The phenotypic variance was set to 1 because we had normalized the summary results before meta-analysis; otherwise, parameters were set to their default values. We also applied an extension, moloc [21], that applies this framework to identify the colocalization of 3 signals. For coloc analyses, we considered signals between two traits to be colocalized at posterior probability ≥ 0.8 (i.e PP4 $\geq$ 0.8).

3. Interpretation of Results and Specific Loci
Read Depth vs. Abundance: The authors link eGene detection to cell abundance and read count (per nucleus). It would be valuable to disentangle these variables. What is the beta coefficient for reads-per-nucleus vs. cell abundance in a linear regression framework? Would "reads per sample" or “counts per sample” (aggregate of all nuclei in a cell type) be a better predictor?

We thank the reviewer for this question. In the original analysis, we examined the relationship of the number of eGenes detected for a given cell type with 1) the mean count of RNA-seq reads observed (i.e., the average number of RNA-seq counts in a given cell type across all donors), and 2) the frequency of that cell type. Indeed, both variables are associated with the number of detected eGenes. Yet, because these two variables are correlated, it was not clear which variable was more important. Comparing the regression coefficients is challenging since the read count and abundance fractions are on different scales. Instead, we performed a multiple regression analysis, modeling the number of detected eGenes as the response variable and mean read count and abundance fraction as predictors. We then used a variance partitioning analysis to quantify the variance explained by each predictor. At each cell type level (i.e., class, subclass, and subtype), the mean read count explained substantially more variance in the number of eGenes discovered than cell abundance fraction. This is consistent with our intuition that statistical power to identify genetic regulatory variants for a given gene increases with the number of RNA-seq reads observed. For a given gene, observed counts can increase by 1) increasing gene expression, 2) increasing overall sequencing depth per cell, or 3) increasing the

number of cells sequenced. Therefore mean read count affects power more directly, while increasing the cell-abundance fraction increases power indirectly by boosting the number of RNA-seq reads for that cell type.

The variable “mean read count” is the average across all donors for a given cell type. This is equivalent to the suggested "reads per sample". We also examined abundance as counts per sample, but it was very strongly correlated with the cell abundance fraction and did not explain any variance in the number of detected eGenes.

In the main text, we have added:

Indeed, the number of detected eGenes increases with cell type abundance (**Fig 1D**) and the average read count per cell type (**Fig S3,S4**).

In the Supplement, we have added:

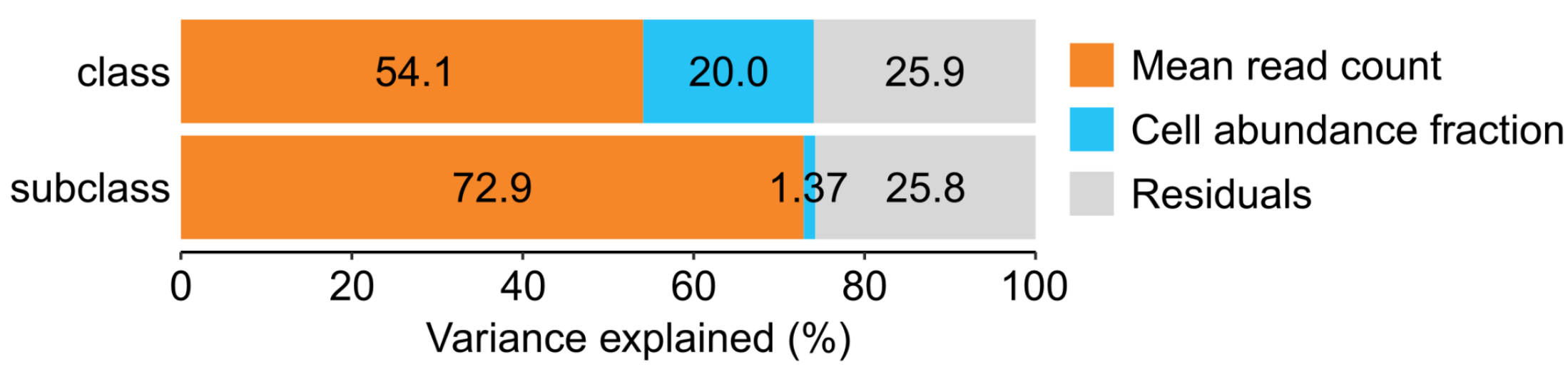

Fig S4. Number of detected eGenes per cell type explained by mean read count. A multiple regression was fitted, modelling the number of detected eGenes as the response variable and mean read count and abundance fraction as predictors. Variance partitioning analysis then quantified the fraction of variance explained by each variable. Analysis was performed for cell class and subclass. At each cell type level, the mean read count explained substantially more variance in the number of eGenes discovered than the cell abundance fraction. This is consistent with our intuition that statistical power to identify genetic regulatory variants for a given gene increases with the number of RNA-seq reads observed. Increasing the cell-abundance fraction increases power, but it does so by increasing the number of RNA-seq reads observed.

Co-localization Inconsistencies:

<u>EGFR:</u> The discussion states the EGFR signal co-localizes with AD risk exclusively in astrocytes at one point, then exclusively in oligodendrocytes later on. The data appears to show colocalization in astrocytes, not oligodendrocytes. Please correct or clarify.

> The text clarifies:
>
> The lead variant for the astrocyte signal is rs74504435, which has a composite posterior probability of 0.946 that it is associated only with EGFR expression in astrocytes (**Fig 3C**). This genetic regulatory signal in astrocytes colocalizes with risk for AD, whereas the regulatory signal in oligodendrocytes is not associated with AD risk, highlighting the cell-type-specific role of disease biology (**Fig 3D**).

<u>Multiple Genes at Loci:</u> The authors claim only 2 genes share a locus, but I identified several others (e.g., EGFR/EGFR-AS1, CR1/CR2, BIN1/CYP27C1). This impacts GWAS-eQTL interpretation and should be visualized/discussed.

> We thank the Reviewer for raising this issue. The discrepancy was that we previously used a p-value filter on the GWAS results when counting loci with multiple genes with colocalization signals. We agree that this was incorrect and have now resolved the issue.
>
> We have updated the text and added a Supplementary Table:
>
> We also note 8 loci on the class level and 11 on the subclass level that contain multiple genes with colocalization signals within 1 Mb (**Table S2**).
>
> **Table S2: Loci with eQTL-GWAS colocalization for multiple genes.**
> Table_S2_coloc_multiple.xlsx

<u>Figure 4F</u>: Several highlighted genes (e.g., TRIM24, NDUFAF6) do not appear in Figure 2D (GWAS-eQTL colocalization). If these do not colocalize with AD GWAS, why are they highlighted in this context?

> We thank the Reviewer for raising this issue. In order to examine the dynamic eQTLs in more detail, the cutoff for the eQTL-GWAS colocalization was relaxed to PP4 > 0.5. TRIM24 and NDUFAF6 were included due to this looser criterion.
>
> We have revised the captions for **Fig 4F** to clarify:

F) Genes with a dynamic eQTL (shown in the inset of each cell class) that have a colocalization signal for AD genetic risk (shown on the x-axis). For example, the gene EGFR has a dynamic eQTL detected in EN, and regulatory variants for this gene colocalize with AD risk in astrocytes. The color and size of the circle indicate posterior probability from colocalization analysis. A colocalization cutoff of PP4 > 0.5 is used, so some genes filtered out above were included here.

Co-localization follow-ups:

Is there any functional enrichment in cell type specific co-localization results? For example, are the immune AD co-localized genes enriched in different functions compared to the oligodendrocyte AD co-localized genes?

We thank the Reviewer for this suggestion. We have performed this enrichment analysis and added it to the Results:

Microglia genes were enriched for pathways involved in amyloid-beta formation and APP processing, consistent with the established role of microglia in plaque biology. Excitatory and inhibitory neurons were enriched for thyroid hormone response and peptide catabolic processes, suggesting neuron-intrinsic regulatory mechanisms influencing amyloid metabolism. Oligodendrocytes were enriched for complement activation and humoral immune response pathways, highlighting a potential immune-regulatory role for oligodendrocytes in AD pathogenesis (**Fig S11**).

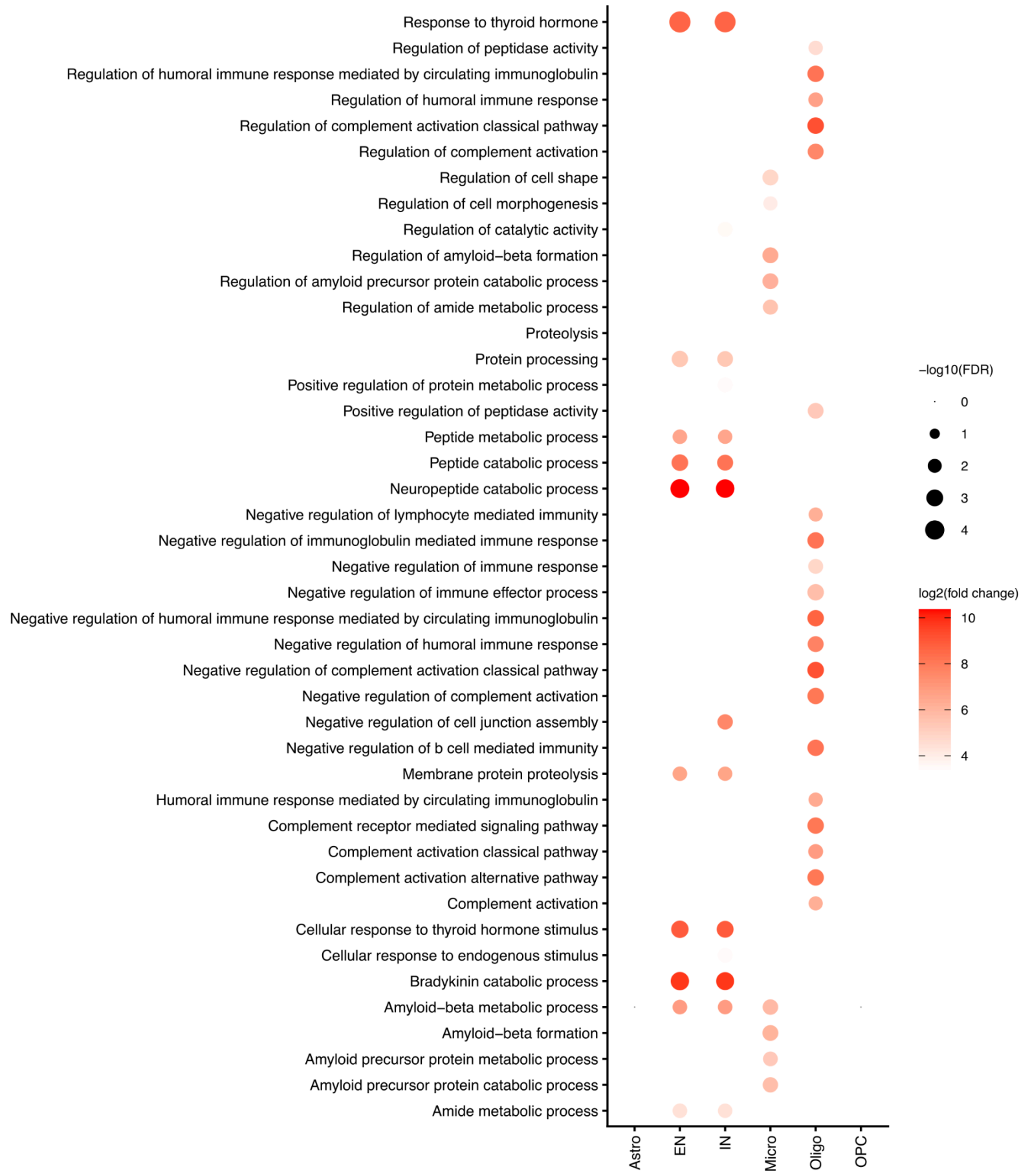

Figure S11: Gene set enrichment analysis for each cell class. Enrichment analysis was performed on the genes colocalizing with AD GWAS in each cell class.

Trans-eQTLs (Figures S16, S18):

Are the trans-eQTLs also cis-eQTLs or GWAS SNPs? Any enrichment for one category vs the other?

> We thank the Reviewer for this question. The trans-eQTL analysis was performed using 56,204 eSNPs from the *cis*-eQTL analysis, and 45,088 genome-wide significant variants from 8 brain disease GWAS studies. Of the genome-wide significant trans-eQTLs, 1460 were cis-eQTLs, and 238 were selected from GWAS variants. Using a Fisher's exact test, these are enriched for cis-eQTLs with a log odds ratio 1.61 (OR=5.02), and a p-value 1.0e-162. However, we are very cautious about overinterpreting this result and have not included it in the text.

Figure S18: Please add a supplementary table clarifying the index SNP ID, cis-gene, trans-gene, cis cell type, trans cell type, and colocalization probabilities. The current display makes it difficult to distinguish if the gene on the left is the cis-gene or the trans-gene and both are needed for proper interpretation of the results.

We have updated the text and included this information in **Table S3**:

Intersecting *trans*-eGenes with genes exhibiting cis-regulatory signals that colocalize with disease risk, we identify 12 genes with colocalization probabilities > 0.8 and 32 with colocalization probabilities > 0.5 (**Fig S21, Table S3**).

Table S3: Intersection of trans-eGenes and cis-eGenes with disease colocalization
Table_S3_cis_trans_coloc.xlsx

Statement on SZ Risk: The claim that RERE and AUTS2 colocalize with SZ risk should be toned down, as the probabilities (0.7 and 0.68) are not very high and below what is typically considered significant.

We thank the Reviewer for this feedback. We have revised the text:

Moreover, colocalization analysis revealed that the *cis*-regulation of *RERE* and the *trans*-regulation of *AUTS2* colocalize, albeit weakly, with the genetic risk for SZ (**Fig 5E**).

4. Reproducibility and Code

Demographics: Basic demographic tables (age, sex, etc.) for the 1,384 donors are missing. Referring to a companion paper is insufficient for evaluating the specific samples used here. Especially as the companion paper did not need the genotype information and, it's reasonable to expect that not 100% of the samples have both snRNA-seq data and genotype data.

We have included the following new plot of the demographics of the subjects included in this study:

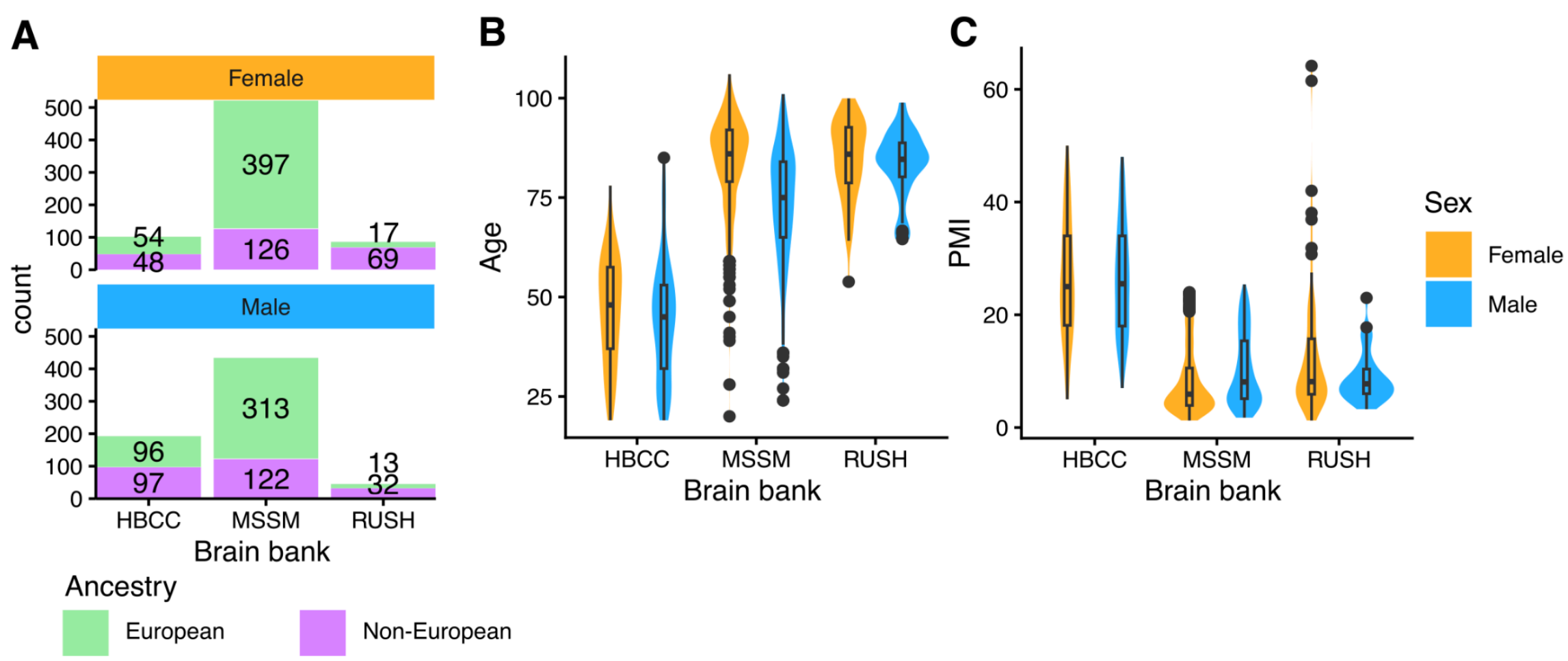

Fig S1. Demographics of subjects included in analysis. A) Count of male and female subjects in each cohort and ancestry group. **B**) Age distribution of subjects stratified by sex and cohort. **C**) Postmortem interval (PMI, in hours) distribution of subjects stratified by sex and cohort.

Code Availability: The provided GitHub repository appears incomplete. I could find plotting scripts but not the scripts for calculating colocalization or the QC steps done on the single cell data for example.

We have included additional detail to the GitHub page: https://github.com/DiseaseNeuroGenomics/nps_ad/

- Results available on Synapse

Analysis code folder

- preprocessing code
- analysis of eQTL summary statistics code
- coloc analysis code
- fine mapping code
- analysis with dreamlet code
- analysis with crumblr code

Missing Supplementary Data: Supplementary tables are missing and necessary (e.g., number of samples per cohort, demographics per study, eQTLs detected, co-localization results, SNP ids of best eQTL per gene for each cell type, etc…). There are a number of files on synapse but these are not well described and may not always be available.

We have improved the description in the Synapse repository:
https://www.synapse.org/Synapse:syn61929918/wiki/629719

Given the scale of the eQTL results, it is impractical to store them as Excel files for Supplementary Tables.

File descriptions

Cis-eQTL results in each cell type from meta-analysis across cohorts: **syn61930218**
Coloc analysis for class (**syn61930113**) and subclass (**syn61930114**)
Dynamic eQTL analysis for all (**syn61930091**) and significant genes (**syn61930104**)
Bayesian multivariate meta-analysis (i.e. mashr) local false positive rates for class (**syn61930162**) and subclass (**syn61930164**)
Cell type specific eQTLs for class (**syn61930119**) and subclass (**syn61930125**)
trans-eQTL results for all genes (**syn63854990**)
Merge of cell type specific and color results: **syn61930118**
Merge of mashr and coloc results (**syn62090962**)
Color palette: **syn62090906**

The authors need to point out the location of the raw data (even if controlled access). This is necessary both for reproducibility purposes and for enabling future meta-analysis of human brain single cell eQTL data.

We have added more detail as requested:

Results from this analysis are available from https://www.synapse.org/Synapse:syn61929918/wiki/629719. Raw and processed data are described by Fullard, et al. [22] and are available at https://www.synapse.org/Synapse:syn60084804/wiki/628473.

Minor Comments:

Structure/Flow: The results section mentions fine-mapping results (association with allelic size) before the fine-mapping methodology or concept is introduced. A brief introduction to the fine-mapping approach and results earlier in the text would improve readability.

We thank the Reviewer for this suggestion. The first paragraph of the Results now ends with:

We then conducted fine-mapping to refine the set of candidate causal variants as regulatory ones at cis-eQTL loci. Across all significant loci, the median 95% credible set size ranged from 10 to 22 variants at the class level, and 10 to 30 variants at the subclass level (**Table S1**).

Figure 3A Visualization: It would be informative to highlight the cell-type specificity of the colocalization signals directly in the figure. For example, explicitly labeling that EGFR colocalizes in astrocytes but not oligodendrocytes, or clarifying if INPP5D co-localization signals are in microglia, astrocytes, or both.

We thank the Reviewer for raising this issue. Zooming in on the panel if **Fig 3A** in question, the two rows for EGFR indicate that one regulatory signal is from astrocytes while the other is from oligodendrocytes, as further detailed in **Fig 3D**.

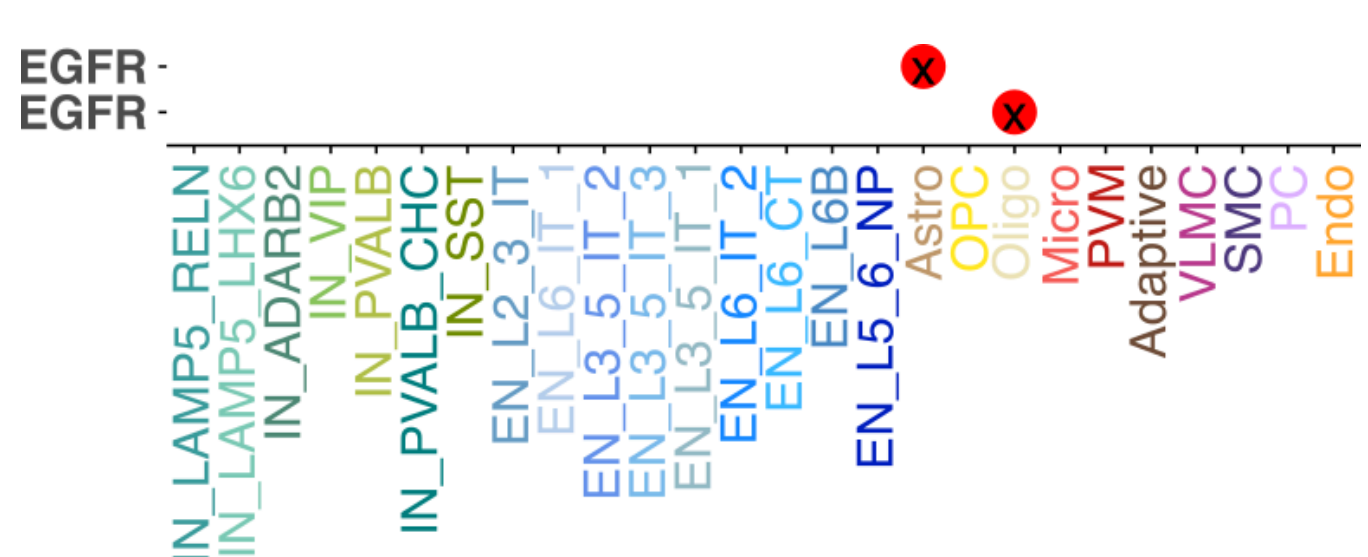

We have revised the caption to clarify:

Figure 3: Cell type specificity of genetic regulatory effects.
A) Cell-type-specific and shared genetic effects for a set of genes across each subclass. The posterior probability that the effect of the lead genetic variant is non-zero for each gene is indicated by the $-\log_{10}$ local false sign rate. Genes shown twice have independent regulatory signals in different cell types. Genes were selected based on cell type specificity, except that the bottom 6 genes were selected based on biological interest. The right column indicates genetic colocalization between eQTL and GWAS for the specified trait. For example, EGFR has independent signals in astrocytes and oligodendrocytes, while INPP5D has independent signals in astrocytes and microglia.

Figure S16 (Trans-eQTLs): A scatterplot similar to the cis-eQTL plot would be informative here to visualize whether trans-eQTLs are more cell-type specific than cis-eQTLs.

We thank the Reviewer for this suggestion. Currently, Figure 5A (reproduced here) shows that trans-eQTLs tend to be genome-wide significant in just one cell type. Given the limited power to detect trans-eQTLs, we have very little power to detect if a trans-eQTL is due to cell-type-specific biological effects. The scatterplot suggested does not visualize this well, and we have not found another way to show this visually, given the very limited power to detect cell-type-specific biological effects.

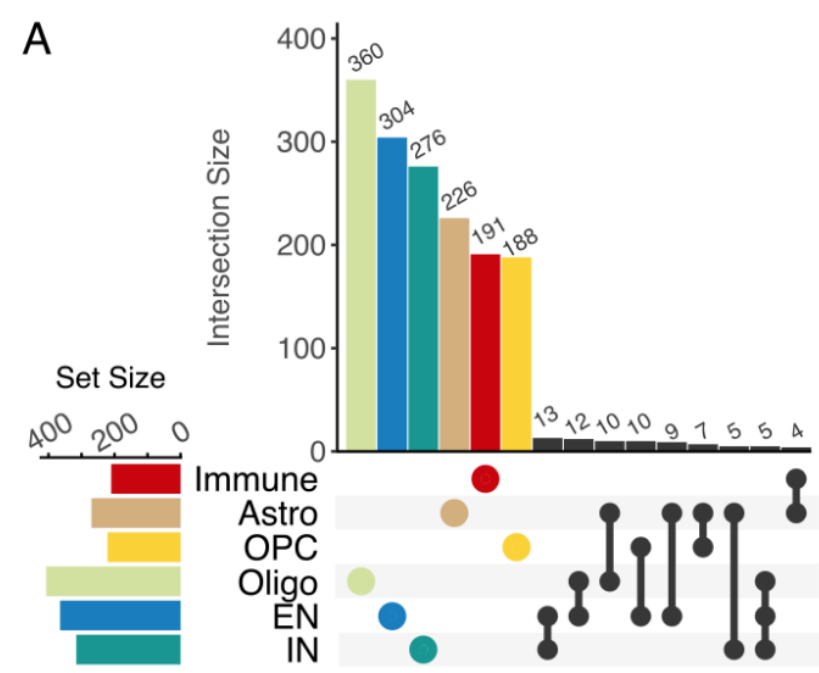

Trans-eQTL Selection: Please clarify how the 56,204 eSNPs and 45,088 GWAS variants were selected for trans-eQTL detection. Was this based solely on a p-value threshold (e.g., $p < 1e-8$)? If so, please discuss how you accounted for LD structure to prevent high false-positive rates from loci with many significant SNPs compared to loci with low LD (and hence fewer SNPs and fewer statistical tests).

We thank the Reviewer for raising this issue, and we have included additional detail. We used only lead variants from eQTL and GWAS analyses and did not include additional variants based on LD. This was done in order to limit computational requirements and the multiple testing burden.

We have revised the text:

Following previous work [23], we addressed both of these issues by selecting lead variants, including 56,204 eSNPs from the *cis*-eQTL analysis and 45,088 genome-wide significant variants from 8 brain disease GWAS studies (i.e., variants with $p<5e-8$ in AD, binge eating disorder, bipolar disorder, MDD, PD, SCZ, ADHD, ASD).

<u>Trans-Regulatory Hubs:</u> Regarding the three identified trans-regulatory hubs, do they affect biologically relevant genes in cis (e.g., Transcription Factors)?

We thank the Reviewer for this question. We identified regulatory hubs with cis-eQTLs for the transcription factors SUPT3H, RUNX2, and ZNF160.

- SUPT3H functions as a SAGA complex co-activator involved in chromatin remodeling,
- RUNX2 is a lineage-defining transcription factor essential for osteoblast differentiation and skeletal development,
- ZNF160 is a KRAB zinc-finger protein likely acting as a transcriptional repressor.

We have updated the Results to read:

Analysis of genetic variants associated with multiple *trans*-eGenes identified 4 *trans*-regulatory hubs centered at 3 transcriptional regulators (SUPT3H, RUNX2, and ZNF160), each influencing at least three target genes, with the largest hub regulating nine downstream targets in oligodendrocytes (**Fig 5B, Fig S20**).

<u>Multiple Testing:</u> The multiple testing procedure for eQTL mapping is unclear. Did the authors use Bonferroni correction for the best eQTL per gene followed by Benjamini-Hochberg across genes (as in Zeng et al., 2022)? Please clarify.

We thank the Reviewer for raising this issue. We have included additional detail:

Multiple testing correction was performed using a two-step Benjamini–Hochberg (BH) false discovery rate (FDR) control framework, as done previously [24]. First, for each gene, all tested variants within the cis-window were adjusted using the BH procedure to control the FDR across variants tested for that gene. Second, we applied an across-gene correction by applying BH across genes to control the genome-wide FDR.

Were all genes tested for eQTL analysis? Or only the expressed ones? Or the ones with a variance above a specific threshold?

Only expressed genes were used for the eQTL analysis. We have included additional detail in the Methods:

Analysis was performed at the pseudobulk level. For each cell type, donors with at least 5 nuclei were retained in order to ensure stable donor-level expression estimates and to reduce noise from sparse sampling. Genes were filtered within each cell type to retain only those that were robustly expressed. This was implemented using edgeR::filterByExpr() and required that at least 40% of donors have a minimum count of 5 reads per gene.

Is the sample size for the eQTL mapping the same for all cell types? It's quite common that some samples are dropped for rare cell types as they contain few (or no) nuclei from the rare population.

We have included a Supplementary table of the sample size for each class and subclass after filtering.

Methods:
After QC and filtering, the number of donors retained for each cohort varied (**Table S4).**

Table S4. Sample size for each cell class and subclass for eQTL analysis after filtering.
Table_S4_SampleSize.xlsx

Reviewer #5 (Remarks to the Author):

The inclusion of the replication cohorts adequately addresses many of the concerns raised in the previous reviews.

We thank the reviewer for these thoughtful and constructive comments.

Minor concerns or points of clarification remain:

Without functional validation, the authors should be careful to not overstate their findings that implicate previously unreported genes. The authors' inclusion of the replication cohort to strengthen confidence in results is a good initial step, but regardless, without other independent lines of evidence, the conclusions claiming novel should not be over-stated. One example of text that should be toned down accordingly is the following statement in the abstract: "novel cell type-specific genes implicated in Alzheimer's disease [...]".

We agree and thank the reviewer for highlighting this point. We have revised the abstract and relevant sections of the manuscript to **avoid overstatement of novelty**. Specifically, we have replaced language implying definitive implication with phrasing that emphasizes **prioritization and association**, for example:

"previously unreported candidate genes with cell-type-specific regulatory associations relevant to Alzheimer's disease"

Similar wording changes have been made throughout the Results and Discussion to clearly distinguish statistical association from functional validation.

Within the following text that was added within the Results: "Genome-wide, eQTL results were highly concordant with results from two single-nucleus datasets and had the highest concordance for matching cell types (Bryois et al. 2022; Fujita et al. 2024) (Fig S1)." please include a quantitative metric to back up the qualitative descriptor ("highly concordant") for precision and to improve clarity.

We have revised the sentence to read:
Genome-wide, eQTL results were highly concordant with results from two single-nucleus datasets and had the highest concordance for matching cell types with π_1 ranging from 0.73 to 0.82 for Bryois, et al. [5], and π_1 ranging from 0.84 to 0.91 for Fujita, et al. [25] (**Fig S2**).

Similarly, please quantify the following qualitative statement that was added to the results: "Using stricter posterior probability cutoffs retains a substantial number of cell-type-specific effects (Figure S12)", specifically what is meant by "substantial number".

> The previous text was ambiguous; we have revised the text to read:
> Using stricter posterior probability cutoffs at 0.8 retains cell-type-specific effects: 630 at the class level, and 644 at the subclass level (**Figure S15**)

The authors explain how they restricted the analysis of trans-regulatory effects to the autosomes, but presumably the analysis of cis-effects was also carried out on chrX (it would be helpful to be explicit in the main text). In the partitioning heritability analysis, did the authors extend the tool to include chrX or did they only examine autosomal data?

> We thank the reviewer for noting this ambiguity. We have revised the text to clarify that the analysis included only autosomes due to the increased statistical and biological complexity associated with modeling long-range regulatory
>
> Cis-eQTLs: Cis-eQTL analyses were performed exclusively on autosomal chromosomes. Variants and genes located on chromosome X were excluded.
>
> Trans-eQTLs: Analysis was performed across all expressed genes in each cell class, and restricted to autosomes, and excluded chromosome X due to the increased statistical and biological complexity associated with modeling long-range regulatory effects involving sex chromosomes, yielding 8.74 billion tests. SNP genotype was included as the dependent variable in all gene-variant pairings in the linear regression model we tested.
>
> Partitioned heritability: Partitioned heritability analyses were conducted using autosomal variants only, consistent with standard S-LDSC analysis.

Also for the partitioning heritability analysis, it would be important for the authors to note what they used as the LD reference, given that the tool requires LD information that matches the inputted summary statistics; what was used is not so straightforward/obvious given the genetic ancestry diversity of the eQTL data. Finally, note the small typo in this section: the authors interchangeably use S-LDSR and S-LDSC in the main text and in the response to reviewers - best to be consistent to avoid confusion.

> We have now explicitly stated that partitioned heritability analyses used the 1000 Genomes Phase 3 European ancestry reference panel. We acknowledge the genetic diversity of the eQTL cohort and now discuss this limitation explicitly in

the Methods and Discussion. We have standardized terminology throughout the manuscript and response letter to use **S-LDSC** consistently.

The authors' response to the Review 3's comment on investigation of ASE to validate the eQTL results is confusing. They respond that "the eQTL and ASE effects show significant concordance"; in the figure they provide, the p-value for the correlation is significant, but the r2 is close to 0. So the allelic effects and the eQTL effect sizes are significantly not-correlated?

We thank the reviewer for this important point and agree that our earlier wording did not sufficiently clarify how to interpret the ASE–eQTL concordance analysis.

We clarify that we do not interpret the ASE analysis as demonstrating strong proportionality in effect sizes between ASE and eQTL estimates. Indeed, as the reviewer notes, the coefficient of determination from the linear regression is modest ($r^2 = 0.027$), indicating that ASE explains only a fraction of the variance in eQTL effect sizes. This is expected given substantial differences between the two measurements, including scale differences, technical noise in ASE estimation, and the fact that ASE is measured in a subset of heterozygous individuals with variable read depth.

However, despite the small r^2, the relationship is highly statistically significant ($p = 6.8\times10^{-11}$), indicating a reproducible positive association between ASE and eQTL effect estimates across a large number of loci. To further demonstrate that this association is not driven by outliers or distributional assumptions, we additionally assessed concordance using Spearman's rank correlation, which yielded a positive correlation ($\rho = 0.14$, $p = 1.4\times10^{-8}$).

Taken together, these results indicate that <u>ASE and eQTL effect estimates are weakly but consistently correlated</u>, supporting the conclusion that ASE provides independent, albeit noisy, evidence in support of the population-level eQTL signals. We have revised the text to explicitly acknowledge the modest effect size while emphasizing the robustness and reproducibility of the association, and have updated the figure legend accordingly to avoid over-interpretation.

Concordance between eQTL and ASE is limited by fine-mapping resolution. We expected that SNPs with higher PIP show higher concordance. Variants are stratified by fine-mapping posterior inclusion probability (PIP > 0.25 vs. ≤ 0.25). Linear regression lines are fitted separately for each group. Variants with higher PIP exhibit stronger concordance between eQTL and ASE effects, supporting the consistency of genetically driven regulatory effects. Despite modest variance

explained at the single-variant level, the significant linear association indicates reproducible directional agreement between population-level eQTL effects and within-individual allelic imbalance.

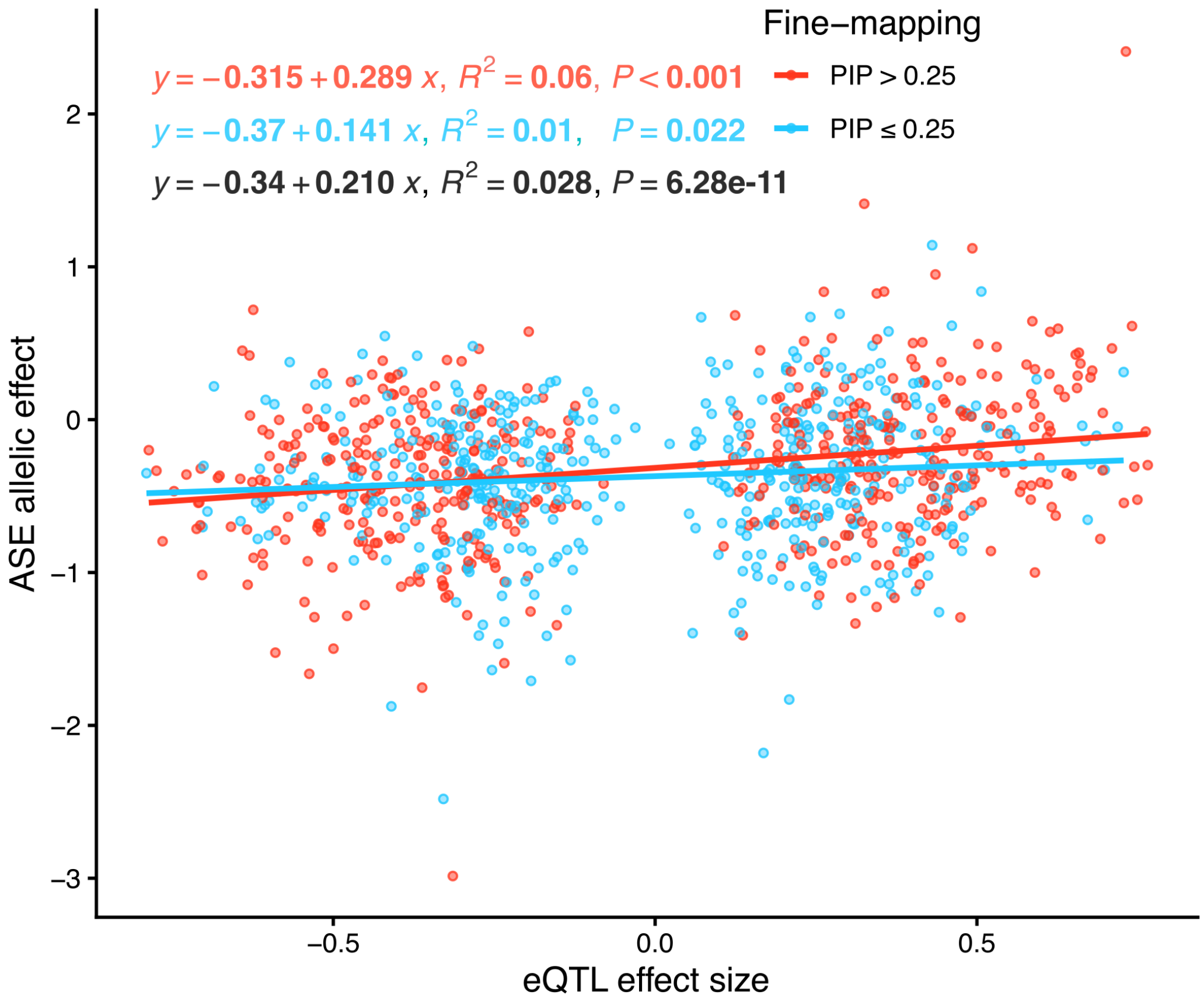

Concordance between eQTL effect sizes and ASE allelic effects.
Scatterplot of eQTL effect sizes versus allele-specific expression (ASE) allelic effects across tested variants. Variants are stratified by fine-mapping posterior inclusion probability (PIP > 0.25 vs. ≤ 0.25). Linear regression lines are fitted separately for each group. Regression equations, coefficient of determination (R^2), and corresponding two-sided P values are shown on the plot. Black equation indicates best fit line without stratifying by PIP.

References

1. Fleming, S. J. *et al.* Unsupervised removal of systematic background noise from droplet-based single-cell experiments using CellBender. *Nat. Methods* **20**, 1323–1335 (2023).
2. Young, M. D. & Behjati, S. SoupX removes ambient RNA contamination from droplet-based single-cell RNA sequencing data. *Gigascience* **9**, (2020).
3. Hoffman, G. E. *et al.* Efficient differential expression analysis of large-scale single cell transcriptomics data using dreamlet. *bioRxiv* (2023) doi:10.1101/2023.03.17.533005.
4. Stegle, O., Parts, L., Durbin, R. & Winn, J. A Bayesian framework to account for complex non-genetic factors in gene expression levels greatly increases power in eQTL studies. *PLoS Comput. Biol.* **6**, e1000770 (2010).
5. Bryois, J. *et al.* Cell-type-specific cis-eQTLs in eight human brain cell types identify novel risk genes for psychiatric and neurological disorders. *Nat. Neurosci.* **25**, 1104–1112 (2022).
6. Hormozdiari, F., Kostem, E., Kang, E. Y., Pasaniuc, B. & Eskin, E. Identifying causal variants at loci with multiple signals of association. *Genetics* **198**, 497–508 (2014).
7. Finucane, H. K. *et al.* Heritability enrichment of specifically expressed genes identifies disease-relevant tissues and cell types. *Nat. Genet.* **50**, 621–629 (2018).
8. Hormozdiari, F. *et al.* Leveraging molecular quantitative trait loci to understand the genetic architecture of diseases and complex traits. *Nat. Genet.* **50**, 1041–1047 (2018).
9. Gazal, S., Marquez-Luna, C., Finucane, H. K. & Price, A. L. Reconciling S-LDSC and LDAK functional enrichment estimates. *Nat. Genet.* **51**, 1202–1204 (2019).
10. Finucane, H. K. *et al.* Partitioning heritability by functional annotation using genome-wide association summary statistics. *Nat. Genet.* **47**, 1228–1235 (2015).
11. Kosoy, R. *et al.* Genetics of the human microglia regulome refines Alzheimer's disease risk loci. *Nat. Genet.* **54**, 1145–1154 (2022).

12. Zeng, B. *et al.* Genetic regulation of cell type–specific chromatin accessibility shapes brain disease etiology. *Science* **384**, eadh4265 (2024).

13. Wingo, A. P. *et al.* Integrating human brain proteomes with genome-wide association data implicates new proteins in Alzheimer's disease pathogenesis. *Nat. Genet.* **53**, 143–146 (2021).

14. Western, D. *et al.* Proteogenomic analysis of human cerebrospinal fluid identifies neurologically relevant regulation and implicates causal proteins for Alzheimer's disease. *Nat. Genet.* **56**, 2672–2684 (2024).

15. Giambartolomei, C. *et al.* Bayesian test for colocalisation between pairs of genetic association studies using summary statistics. *PLoS Genet.* **10**, e1004383 (2014).

16. Bellenguez, C. *et al.* New insights into the genetic etiology of Alzheimer's disease and related dementias. *Nat. Genet.* **54**, 412–436 (2022).

17. Zattoni, M. *et al.* Serpin signatures in prion and Alzheimer's diseases. *Mol. Neurobiol.* **59**, 3778–3799 (2022).

18. Akasaka-Manya, K. *et al.* Excess APP O-glycosylation by GalNAc-T6 decreases Aβ production. *J. Biochem.* **161**, 99–111 (2017).

19. Howard, D. M. *et al.* Genome-wide meta-analysis of depression identifies 102 independent variants and highlights the importance of the prefrontal brain regions. *Nat. Neurosci.* **22**, 343–352 (2019).

20. Trubetskoy, V. *et al.* Mapping genomic loci implicates genes and synaptic biology in schizophrenia. *Nature* **604**, 502–508 (2022).

21. Giambartolomei, C. *et al.* A Bayesian framework for multiple trait colocalization from summary association statistics. *Bioinformatics* **34**, 2538–2545 (2018).

22. Fullard, J. F. *et al.* Population-scale cross-disorder atlas of the human prefrontal cortex at single-cell resolution. *Sci. Data* **12**, 954 (2025).

23. Yazar, S. *et al.* Single-cell eQTL mapping identifies cell type-specific genetic control of

autoimmune disease. *Science* **376**, eabf3041 (2022).

24. Zeng, B. *et al.* Multi-ancestry eQTL meta-analysis of human brain identifies candidate causal variants for brain-related traits. *Nat. Genet.* **54**, 161–169 (2022).

25. Fujita, M. *et al.* Cell subtype-specific effects of genetic variation in the Alzheimer's disease brain. *Nat. Genet.* **56**, 605–614 (2024).

Response to Reviewers

Reviewer #4 (Remarks to the Author):

The authors have adequately address my comments.

One minor point which has not been corrected. The discussion still states: "We highlight the example of EGFR, which has at least two distinct regulatory programs, with one active in astrocytes and another in oligodendrocytes. Notably, only the regulatory signal in oligodendrocytes colocalizes with the genetic risk of AD, highlighting the importance of cell type-specific regulatory programs in disease biology."

The data shows that it's the astrocyte regulatory signal which co-localize with Alzheimer.

> We have revised the text to read:
>
> Notably, only the regulatory signal in astrocytes colocalizes with the genetic risk of AD, highlighting the importance of cell type-specific regulatory programs in disease biology.

Analysis of X chromosome

Based on the feedback from Reviewer 5, we have included analysis of the X chromosome. The main text reports results from autosomes, but analysis of chrX is included as described below.

We did not include analysis of chrX before since previous single cell brain QTL analysis by Bryois, et al. (2022) and Jang, et al. (2026), did not included chrX, and Fujita, et al. (2024) only included a small subset of SNPs on chrX. In addition, downstream QTL analysis pipelines (i.e. LD-score regression) do not support chrX. Finally, we could not include GWAS colocalization analysis with chrX since the GWAS analyzed here for Alzheimer's disease and major depressive disorder did not include chrX, and the schizophrenia GWAS did not find any genome-wide significant hits on chrX.

We have now included QTL analysis on chrX at the class and subclass levels using the same approach as used by GTEx (Oliva et al., 2020). We have added the following text to the Methods:

> ## Analysis of X chromosome
>
> We performed analysis of chrX at the class and subclass levels using the same approach as used by GTEx (Oliva et al., 2020). For variants located in the non-pseudoautosomal (non-PAR) regions of the X chromosome, we accounted for sex-specific genotype dosage. Because males are hemizygous for the X chromosome, their genotypes were coded as 0

(hemizygous reference) or 2 (hemizygous alternative), effectively doubling the allele dosage to match the diploid scale used for females. Genotypes in females were treated the same as autosomal variants (0, 1, or 2). For variants located in the pseudoautosomal regions (PARs) of the X chromosome, genotypes were treated as autosomal because these regions are present on both the X and Y chromosomes and behave as diploid in both sexes. We then applied the same analysis pipeline as for autosomal chromosomes to run eQTL detection. Analysis was performed on each brain bank separately and then results were combined using a fixed effect meta-analysis. Effect size estimates showed high concordance across cell types with sign concordance rate ranging from 84.8 to 97.8 % (**Fig S26**). Analysis discovered between 8 and 301 eGenes at the class level and between 1 and 213 eGenes at the subclass level (**Fig S27**).

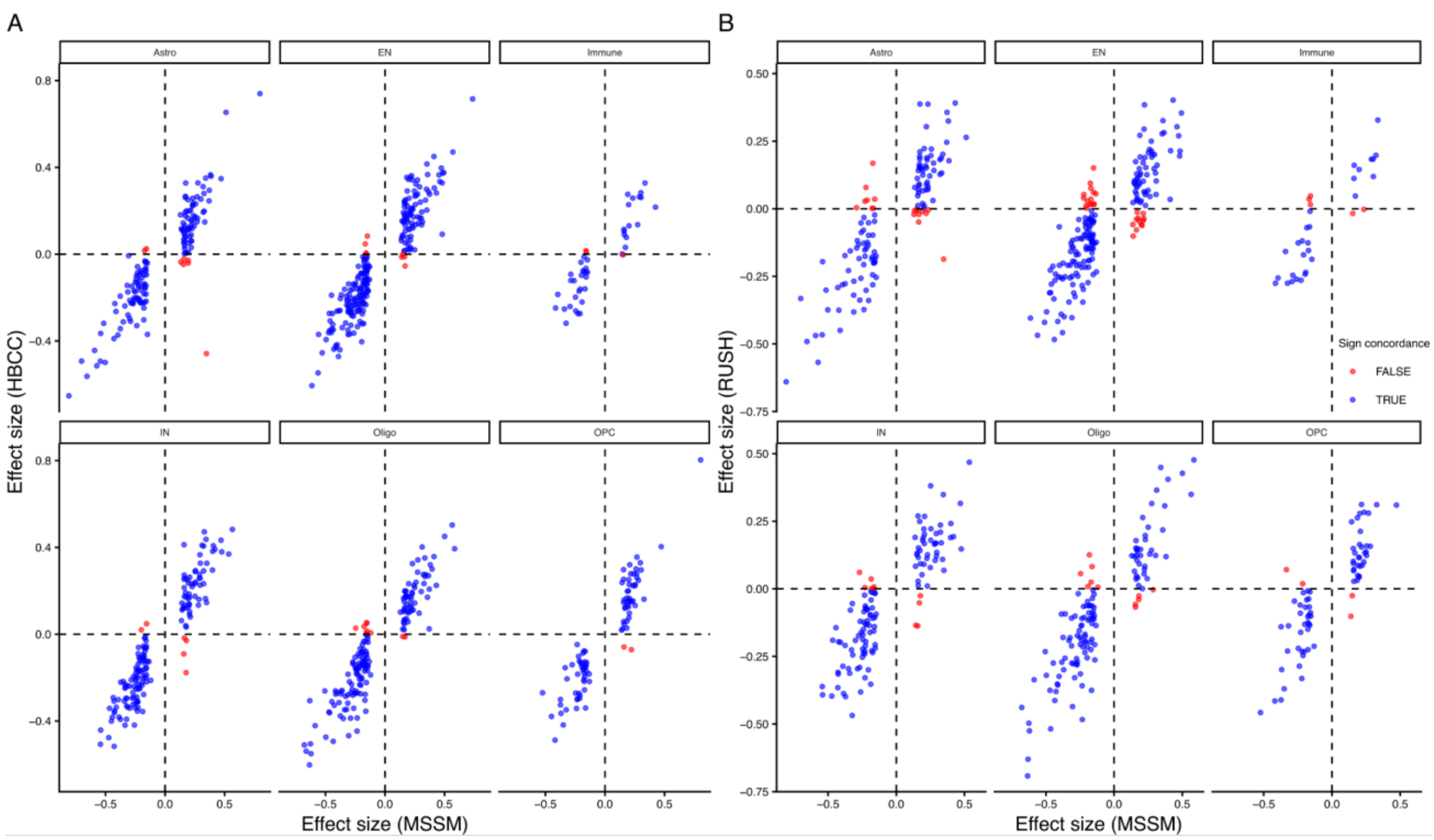

Figure S26. Effect size concordance across brain banks for X chromosome. Estimated eQTL effect sizes are shown for 3 brain banks for the lead variant from each gene with a genome-wide significant eQTL detected in the MSSM cohort. Points are colored by sign concordance with blue indicating the same sign in both brain banks and red indicating different signs. **A**) Effect sizes from HBCC shown against eQTLs discovered in MSSM. **B**) Effect sizes from RADC shown against eQTLs discovered in MSSM.

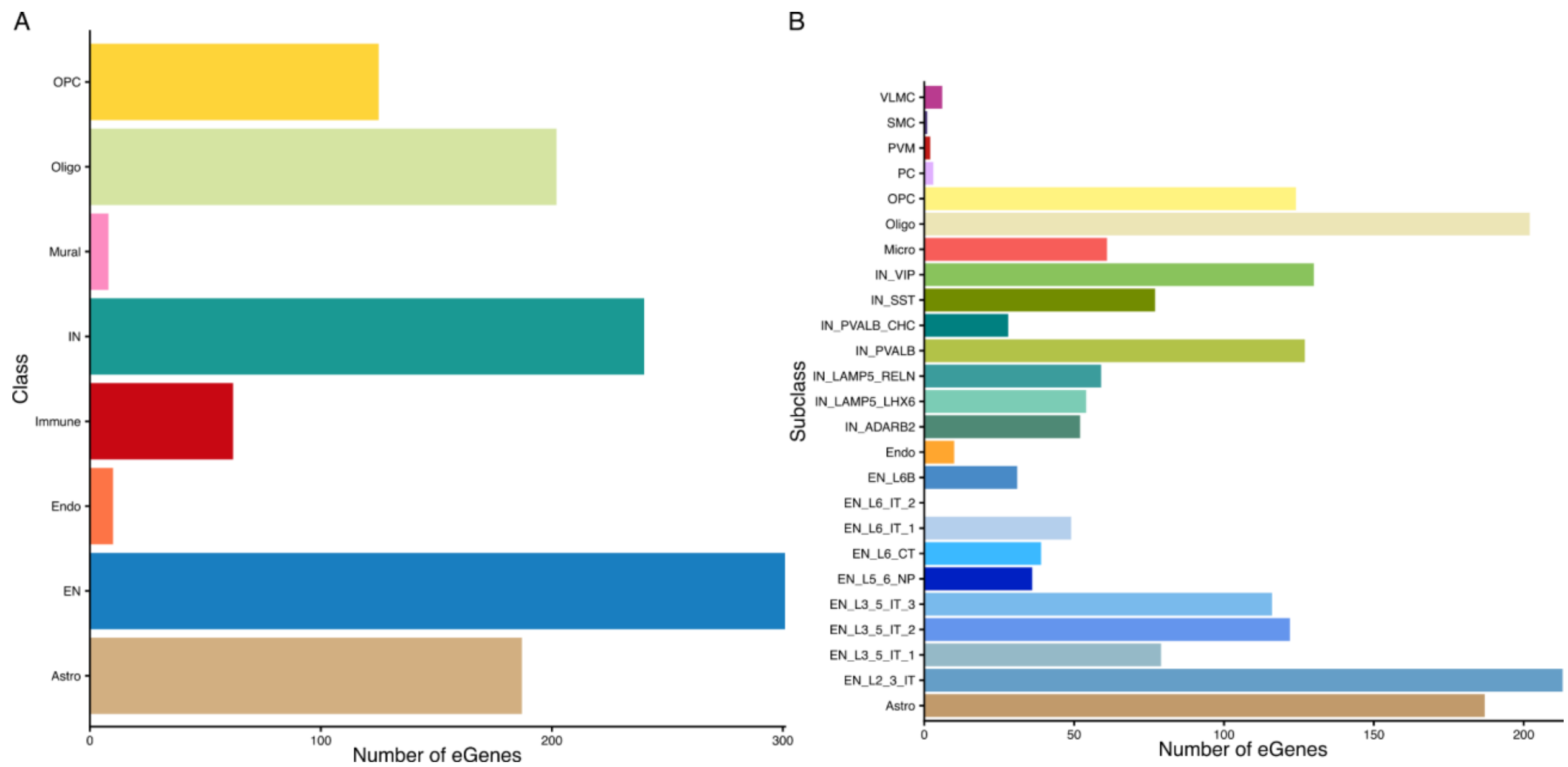

Figure S27. Number of eGenes discovered in the X chromosome. Number of genome-wide significant eGenes on chrX at the **A**) class and **B**) subclass level.

Summary statistics for the X chromosome analysis are available at https://www.synapse.org/Synapse:syn74415475

References

Bryois, J. et al. (2022). Cell-type-specific cis-eQTLs in eight human brain cell types identify novel risk genes for psychiatric and neurological disorders. *Nature Neuroscience*, 25 (8), pp.1104–1112.

Fujita, M. et al. (2024). Cell subtype-specific effects of genetic variation in the Alzheimer's disease brain. *Nature Genetics*, 56 (4), pp.605–614.

Jang, B. et al. (2026). A meta-analysis of single-nucleus expression quantitative trait loci linking genetic risk to brain disorders. *Nature Genetics*. [Online]. Available at: doi:10.1038/s41588-026-02541-x.

Oliva, M. et al. (2020). The impact of sex on gene expression across human tissues. *Science*, 369 (6509). [Online]. Available at: doi:10.1126/science.aba3066.
